# Supplementary material for: Clinicians’ prescribing pattern, rate of patients’ medication adherence and its determinants among adult hypertensive patients at Jimma University Medical Center: Prospective cohort study
Source: PLoS One. 2021 Nov 15;16(11):e0259421. doi: 10.1371/journal.pone.0259421 (PMC8592482; doi:10.1371/journal.pone.0259421)
Supplement: S1 File — (DOCX) [file pone.0259421.s002.docx]

**Questionnaire**

**Part one:**

I. Participants’ Scio demographic Characteristics and current life style conditions

Card number_______________ Date_______________

Date of Appointment in the next visit (V1__________ V2___________ V3 ____________)

| No- | Questions | Responses |
| --- | --- | --- |
| 1 | Age | ______years |
| 2 | Sex | Male  Female |
| 3 | Height | ______ cm |
| 4 | Body weight | _______kg |
| 5 | Marital status | 1. Single  2. Married  3. Divorced  4. Widowed  |
| 6 | Residence | 1. Urban  2. Rural  |
| 7 | Education level | 1. No formal education  2. Primary education (1-8 grade)   3. Secondary education (9 -12 grade)   4.Tertiary education (diploma and above) |
| 8 | Current occupation | Civil servant Merchant  Farmer  House wife  Retired Jobless  Other _______________ |
| 9 | Family monthly income (Birr) | 1) < 1000 2) 1001- 2000  3) 2001-3000  4) > 3000  5) without a well-defined monthly wage |
| 10 | Living status | I live alone  Other _______________  I live with family |
| 11 | Reduce salt intake | Yes  No |
| 12 | Alcohol use | Yes  No |
| 13 | Chewing chat | yes  No |
| 14 | Smoking | Never smoked  Ex-smoker Current smoker |
| 15 | Physical activity | Physically active  Physically inactive |
| 16 | Drink coffee | Yes  No |
| 17 | Use traditional medicine | Yes  No |

**II. Eight Item Morisky Medication Adherence Predictor Scale, (MMAPS)**

| S.N | MMAPS questions | Yes | No |
| --- | --- | --- | --- |
| 1 | Do you sometimes forget to take your pills? |  |  |
| 2 | People sometimes miss taking their medications for reasons other than forgetting. Thinking over the past two weeks, were there any days when you did not take your medicine? |  |  |
| 3 | Have you ever cut back or stopped taking your medicine without telling your doctor because you felt worse when you took it? |  |  |
| 4 | When you travel or leave home, do you sometimes forget to bring along your medicine? |  |  |
| 5 | Did not you take all your medicine yesterday? |  |  |
| 6 | When you feel like your symptoms are under control, do you sometimes stop taking your medicine |  |  |
| 7 | Taking medicine every day is a real inconvenience for some people. Do you ever feel hassled about sticking to your treatment plan? |  |  |
| 8 | How often do you have difficulty remembering to take all your medicine?  A)  Never/rarely B)  Once in a while C)  Sometimes D)  Usually E)   All the time |  |  |

9. In addition to the above, do the following may be problems to adhere to your medication?

The drug is expensive  Drugs not available in the market I feel bad when I take it

(Side effect)

**III. Clinician’s characteristics and perception related to guideline adherence**

| S.N | Questions | Responses |
| --- | --- | --- |
| 1 | Age | ______years |
| 2 | Sex |  Male Female |
| 3 | Position of physician | General practitioner (GP)   Resident:  R_1_ R_2_R_3_   Internist  Cardiologist   Others______________ |
| 4 | Years of experience  (years) |  <1  1-5  >5 |
| 5 | Common perception  of physician to JNC -8 guideline (possible to select more than one) | Evidence based Too complicated  Restrict physicians’ autonomy Too many guidelines Theoretical, not practice oriented  Not applicable at the individual level  Financial constrains by the health insurance  Helpful in managing patients  Decrease the treatment cost |
| 6 | Work load (approximate number of patients seen per clinic day) | <10 10-20 >20 |
| 7 | Other possible factors affecting guideline adherence (possible to select more than one) | The drug is expensive Drugs not available   presence of co morbidity   stage of hypertension ( un control BP) |

**Part Two: Data Abstraction format from Patients’ Chart**

1. Patient related

A. Duration of treatment  <1  1-5  >5-10  > 10

B.Documented/suspected ADR/Allergy if any__________________________

**2. Co- morbid condition**

2.1. Hypertension alone Yes  No

2.2. Diabetes Mellitus Yes  No

2.3. Cardio vascular diseases Yes (specify): ________________  No

2.4. Renal diseases Yes  No

2.5. Liver Diseases Yes  No

2.6. Lungs Yes  No

2.7. Obesity/overweight Yes  No

2.8. Dyslipidemia Yes  No

2.9. Thyroid disease Yes  No

2.10. Others  yes (specify: ________________  No

3. **Blood pressure records (both SBP and DBP)**

3.1 Base line (2 records before data collection period)

**A.**________________ mmHg B. ________________ mmHg

3.2. Visit 1 ________________ mmHg

3.3. Visit 2(2^nd^ month) ________________ mmHg

3.4. visit 3(3^rd^ month^)^ ________________ mmHg

**4. Antihypertensive prescribed (Drug type, dose, frequency and duration) (write V1, V2, V3 in bracket to represent visiting time)**

4.1. ACE inhibitor  Yes  No

 Enalapril ________________  captopril ________________

4.2. ARB  Yes  No

 Losartan ________________ valsartan ________________ other ________________

4.3. Diuretics Yes  No

 Hydrochlorothiazide________________  Furosemide________________

 Spironolactone ________________  Hydrochlorothiazide +furosemide

 Furosemide +spironolactone

4.4. CCB Yes  No

Nifedipine________________Amlodipine________________others________________

4.5. BB Yes No

Atenolol________________Metoprolol ________________ Propranolol________________

 Others ________________

4.6. Others  Yes  No  Methyldopa _______________ Hydralazine________________

4.7 Drugs other than antihypertensive  Yes (number of co- medication) ________________

 No

**5. Pattern of therapy (write V1, V2, V3 in bracket to represent visiting time)**

5.1. Non-pharmacologic therapy Yes  No

5.2. Mono-therapy Yes (Drug name) ________________ No

5.3. Two drug therapy Yes No

ACEI + diuretics Diuretics + BB ACEI + CCB ACE + BB Diuretics +ARB

CCB + ARB CCB + Diuretics CCB + BB  ARB +CCB  Other_________

5.4. Three drugs therapy Yes No

 BB + Diuretic + ACEI  BB + Diuretic + ARB  CCB + BB + Diuretic

CCB +ACEI + Diuretic  CCB + BB + ACEI  CCB + ARB + Diuretic

CCB + BB +ARB  others ________________

5.5. Four drugs therapy  Yes  No

ACEI + Diuretics + CCB + BB ARB + BB + CCB + diuretic other____________
