# Supplementary material for: Clinicians’ prescribing pattern, rate of patients’ medication adherence and its determinants among adult hypertensive patients at Jimma University Medical Center: Prospective cohort study
Source: PLoS One. 2021 Nov 15;16(11):e0259421. doi: 10.1371/journal.pone.0259421 (PMC8592482; doi:10.1371/journal.pone.0259421)
Supplement: Attachment — Submitted filename: PONE-D-20-38133_reviewed.pdf [file pone.0259421.s003.pdf]

PLOS ONE

# Clinician’s Prescribing Pattern, Rate of Patients’ Medication Adherence and Its Determinants Among Adult Hypertensive Patients in Ethiopia: Prospective Cohort Study

--Manuscript Draft--

| | |
|---|---|
| Manuscript Number: | PONE-D-20-38133 |
| Article Type: | Research Article |
| Full Title: | Clinician’s Prescribing Pattern, Rate of Patients’ Medication Adherence and Its Determinants Among Adult Hypertensive Patients in Ethiopia: Prospective Cohort Study |
| Short Title: | Clinician’s Prescribing Pattern, Rate of Patients’ Medication Adherence and Its Determinants |
| Corresponding Author: | Bekalu Kebede simegn, MSc<br>Debremarkos Uiversity<br>Debre Markos, Amhara ETHIOPIA |
| Keywords: | Prescribing Pattern, Hypertension, Blood Pressure Control, Guidelines, Adherence |
| Abstract: | Background: Despite the goals for management of hypertension are well defined, effective therapies are widely available, control of hypertension is poor and hypertension related cardiovascular diseases are still high in most health care setting. Therefore, this study aimed to determine clinician’s prescribing pattern, Patients’ Medication Adherence and Its Determinants among ambulatory hypertensive patients at Jimma University Medical Center.<br>Method: General prospective cohort study was conducted among hypertensive patients who had regular follow up at Jimma university ambulatory cardiac clinic from March 20 to June 20, 2018. Patients’ specific data was collected using structured data collection tool directly from patients and their medical chart and from clinicians through self-administered questionnaire. Data was analyzed using SPSS version 21.0. Bivariate and multivariable logistic regression analyses were done to identify key independent variables influencing patients’ adherence. P-Values of less than 0.05 were considered as statically significant.<br>Results: From total of e 416 patients, 237(57.0%) were males with mean age of 56.50 ± 11.96 years. Angiotensin converting enzyme inhibitor was the most frequently prescribed, 261(63.7%) and majority of patients, 275 (66.1%) were on combination therapy. The rate of patients’ medication adherence and clinicians’ guidelines adherence were 46.6%, and 44.2%, respectively. Patient with occupation of Merchant (P=0.020), physical inactivity (P=0.033) and diabetes mellitus co-morbidity (P=0.008) were significantly associated with medication non-adherence.<br>Conclusion: The rate of medication adherence among hypertensive patients was poor and. physicians were not adhering to standard treatment guidelines. Angiotensin converting enzyme inhibitor was the most frequently prescribed class of drugs.<br>Effective education for patients to improve medication adherence and regular training for prescriber on practical treatment guidelines is recommended to keep them up-to-date with current trends of hypertension treatment and for better treatment outcome |
| Order of Authors: | Bekalu Kebede simegn, MSc |
| | Bekalu Dessie Alamirew |
| | Legese chelkeba |
| Additional Information: | |
| Question | Response |
| **Financial Disclosure**<br><br>Enter a financial disclosure statement that describes the sources of funding for the work included in this submission. Review | The funders had no role in study design, data collection and analysis, decision to publish, or preparation of the manuscript. |

Powered by Editorial Manager® and ProduXion Manager® from Aries Systems Corporation

| | |
|---|---|
| the submission guidelines for detailed requirements. View published research articles from *PLOS ONE* for specific examples.<br><br>This statement is required for submission and **will appear in the published article** if the submission is accepted. Please make sure it is accurate.<br><br>**Unfunded studies**<br>Enter: *The author(s) received no specific funding for this work.*<br><br>**Funded studies**<br>Enter a statement with the following details:<br>• Initials of the authors who received each award<br>• Grant numbers awarded to each author<br>• The full name of each funder<br>• URL of each funder website<br>• Did the sponsors or funders play any role in the study design, data collection and analysis, decision to publish, or preparation of the manuscript?<br>• **NO** - Include this sentence at the end of your statement: *The funders had no role in study design, data collection and analysis, decision to publish, or preparation of the manuscript.*<br>• **YES** - Specify the role(s) played.<br><br>* typeset | |
| **Competing Interests**<br><br>Use the instructions below to enter a competing interest statement for this submission. On behalf of all authors, disclose any competing interests that could be perceived to bias this work—acknowledging all financial support and any other relevant financial or non-financial competing interests.<br><br>This statement **will appear in the published article** if the submission is accepted. Please make sure it is accurate. View published research articles from *PLOS ONE* for specific examples. | The authors declared that no competing interests exist. |

*Powered by Editorial Manager® and ProduXion Manager® from Aries Systems Corporation*

| | |
|---|---|
| **NO authors have competing interests**<br><br>Enter: *The authors have declared that no competing interests exist.*<br><br>**Authors with competing interests**<br><br>Enter competing interest details beginning with this statement:<br><br>*I have read the journal's policy and the authors of this manuscript have the following competing interests: [insert competing interests here]*<br><br>* typeset | |
| **Ethics Statement**<br><br>Enter an ethics statement for this submission. This statement is required if the study involved:<br><br>• Human participants<br>• Human specimens or tissue<br>• Vertebrate animals or cephalopods<br>• Vertebrate embryos or tissues<br>• Field research<br><br>Write "N/A" if the submission does not require an ethics statement.<br><br>General guidance is provided below. Consult the submission guidelines for detailed instructions. **Make sure that all information entered here is included in the Methods section of the manuscript.** | The protocol for this study was approved by the Institutional Review Board (IRB)(IHRPGD/203/18), Institute of Health, and Jimma University. Oral consent was obtained from participants before being involved in the study. Confidentiality of data was assured for participants. |

Powered by Editorial Manager® and ProduXion Manager® from Aries Systems Corporation

| | |
|---|---|
| **Format for specific study types**<br><br>**Human Subject Research (involving human participants and/or tissue)**<br>• Give the name of the institutional review board or ethics committee that approved the study<br>• Include the approval number and/or a statement indicating approval of this research<br>• Indicate the form of consent obtained (written/oral) or the reason that consent was not obtained (e.g. the data were analyzed anonymously)<br><br>**Animal Research (involving vertebrate animals, embryos or tissues)**<br>• Provide the name of the Institutional Animal Care and Use Committee (IACUC) or other relevant ethics board that reviewed the study protocol, and indicate whether they approved this research or granted a formal waiver of ethical approval<br>• Include an approval number if one was obtained<br>• If the study involved *non-human primates*, add *additional details* about animal welfare and steps taken to ameliorate suffering<br>• If anesthesia, euthanasia, or any kind of animal sacrifice is part of the study, include briefly which substances and/or methods were applied<br><br>**Field Research**<br><br>Include the following details if this study involves the collection of plant, animal, or other materials from a natural setting:<br>• Field permit number<br>• Name of the institution or relevant body that granted permission | |
| **Data Availability**<br><br>Authors are required to make all data underlying the findings described fully available, without restriction, and from the time of publication. PLOS allows rare exceptions to address legal and ethical concerns. See the PLOS Data Policy and FAQ for detailed information. | No - some restrictions will apply |

Powered by Editorial Manager® and ProduXion Manager® from Aries Systems Corporation

| | |
|---|---|
| A Data Availability Statement describing where the data can be found is required at submission. Your answers to this question constitute the Data Availability Statement and **will be published in the article**, if accepted.<br><br>**Important:** Stating 'data available on request from the author' is not sufficient. If your data are only available upon request, select 'No' for the first question and explain your exceptional situation in the text box.<br><br>Do the authors confirm that all data underlying the findings described in their manuscript are fully available without restriction? | |
| **Describe where the data may be found in full sentences. If you are copying our sample text, replace any instances of XXX with the appropriate details.**<br><br>• If the data are **held or will be held in a public repository**, include URLs, accession numbers or DOIs. If this information will only be available after acceptance, indicate this by ticking the box below. For example: *All XXX files are available from the XXX database (accession number(s) XXX, XXX.).*<br>• If the data are all contained **within the manuscript and/or Supporting Information files**, enter the following: *All relevant data are within the manuscript and its Supporting Information files.*<br>• If neither of these applies but you are able to provide **details of access elsewhere**, with or without limitations, please do so. For example:<br><br>*Data cannot be shared publicly because of [XXX]. Data are available from the XXX Institutional Data Access / Ethics Committee (contact via XXX) for researchers who meet the criteria for access to confidential data.*<br><br>*The data underlying the results presented in the study are available from (include the name of the third party* | All relevant data are within the manuscript and its Supporting Information files |

Powered by Editorial Manager® and ProduXion Manager® from Aries Systems Corporation

| | |
|---|---|
| *and contact information or URL).*<br>• This text is appropriate if the data are owned by a third party and authors do not have permission to share the data.<br><br>* typeset | |
| Additional data availability information: | Tick here if the URLs/accession numbers/DOIs will be available only after acceptance of the manuscript for publication so that we can ensure their inclusion before publication. |

Powered by Editorial Manager® and ProduXion Manager® from Aries Systems Corporation

Click here to access/download;Manuscript;Manuscript.docx

# Clinician's Prescribing Pattern, Rate of Patients' Medication Adherence and Its Determinants Among Adult Hypertensive Patients in Ethiopia: Prospective Cohort Study

Bekalu Kebede[1] MSc, Legese Chelkeba [2] PhD, Bekalu Dessi[illegible]

[1] College of Medicine and Health Sciences, Debre Markos University, Debre Markos, Ethiopia

[2]Department of Clinical Pharmacy, College Health Sciences, Jimma University, Jimma, Ethiopia, email

**Corresponding author: Bekalu Kebede**

Clinical Pharmacist, College of Health, Department of pharmacy, Debre Markos University, Debre Markos, Ethiopia

Phone: +251-921-286-140

Email: bekalukebede19@gmail.com

## Abstract

**Background:** Despite the goals for management of hypertension are well defined, effective therapies are widely available, control of hypertension is poor and hypertension related cardiovascular diseases are still high in most health care setting. Therefore, this study aimed to determine clinician's prescribing pattern, Patients' Medication Adherence and Its Determinants among ambulatory hypertensive patients at Jimma University Medical Center.

**Method:** General prospective cohort study was conducted among hypertensive patients who had regular follow up at Jimma university ambulatory cardiac clinic from March 20 to June 20, 2018. Patients' specific data was collected using structured data collection tool directly from patients and their medical chart and from clinicians through self-administered questionnaire. Data was analyzed using SPSS version 21.0. Bivariate and multivariable logistic regression analyses were done to identify key independent variables influencing patients' adherence. P-Values of less than 0.05 were considered as statically significant.

**Results:** From total of 416 patients, 237(57.0%) were males with mean age of 56.50 ± 11.96 years. Angiotensin converting enzyme inhibitor was the most frequently prescribed, 261(63.7%) and majority of patients, 275 (66.1%) were on combination therapy. The rate of patients' medication adherence and clinicians' guidelines adherence were 46.6%, and 44.2%, respectively. Patient with occupation of Merchant (P=0.020), physical inactivity (P=0.033) and diabetes mellitus co-morbidity (P=0.008) were significantly associated with medication non-adherence.

**Conclusion:** The rate of medication adherence among hypertensive patients was poor a[illegible] physicians were not adhering to standard treatment guidelines. Angiotensin converting enzyme inhibitor was the most frequently prescribed class of drugs. Effective education for patients to improve medication adherence and regular training for prescriber on practical treatment guidelines is recommended to keep them up-to-date with current trends of hypertension treatment and for better treatment outcome.

Keywords: Prescribing Pattern, Hypertension, Blood Pressure Control, Guidelines, Adherence

## Background

Hypertension is an important worldwide public health challenge because of its high frequency and concomitant risks of cardiovascular and kidney disease [1]. The World Health Organization (WHO) 2015 has estimated that high blood pressure (BP) is a major public health issue and causes one in every eight deaths, and being the third leading silent killer in the world[2].

Almost three-quarters of people with hypertension (639 million people) live in developing countries with limited health resources [1, 2]. In Sub- Saharan Africa, it is major, independent risk factor for heart failure, stroke and kidney failure due to insufficient diagnosis of hypertension, suboptimal BP control in the diagnosed patients and increases morbidity and mortality with an increased burden to health care resources [3]. In Ethiopia, Systematic meta-analysis in 2015 showed that the prevalence of hypertension among Ethiopian population was estimated to be 19.6 % [4]. Other systemic meta-analysis from January, 2000 to April, 2015, report that the prevalence of hypertension in Ethiopia between 20% and 30% [5].

Although hypertension is a preventable and modifiable risk factor of cardiovascular diseases (CVD), the prevention and control of hypertension has not yet received attention in many developing countries [6,7]. It is responsible for at least 40.6% of deaths due to all coronary heart disease, 38.5% of deaths due to stroke [8] and second leading assigned cause of end stage renal disease behind diabetes mellitus (DM), and accounted for 34% of incident ESRD [9].

Guideline adherence is defined as a condition in which the prescribed treatment conforms to treatment recommended in the identified practice guidelines [10-12]. Hypertension guidelines, in large part, are evidence based and are usually dictated by randomized controlled trial data and observational studies. Published guidelines help in clinical decision making, decrease practice variations, guide correctness, and measure the quality of health care [13-14]. One of most commonly used guideline which considered the "gold standard" consensus guideline for the management of hypertension was the Eighth Joint National Committee (JNC-8) on detection, evaluation, and treatment of BP [15]. This guideline was used as reference standard for this study

because of awareness and accessibility of guideline were initial criteria to evaluate status of clinician's adherence to hypertension treatment guidelines. In addition, Ethiopia has not its own hypertension treatment guideline and on the time being this guideline widely using by both clinicians and clinical pharmacist [16]. The overall principles common to this guideline are to implement life style modifications in addition to pharmacotherapy to control BP in patients with hypertension. However, clinical practice guidelines do not consistently change clinicians' behavior, control of high blood pressure remain suboptimal, so need to evaluate it' s use and its impact to BP control [10].

Many studies conducted in the past to explore the causes of suboptimal BP control focus on patient's factors such as socio-demographic, medical profile and patient's treatment compliance [17-20]. However other than patient's factor, prescribing pattern and clinicians' adherence to guidelines also played an equally important role in determining the outcome of hypertension treatment [21-23]. Even though, guidelines have been written to provide a straight forward approach to managing hypertension in the community [21], clinical practice guidelines do not consistently change clinicians' behavior [24].

Several studies conducted worldwide using either prescription or drug dispensing data to evaluate prescription pattern and guidelines adherence [25-27]. But few data are available in Ethiopia. In addition, among previous studies, there was conflicting result between prescribing pattern and patient's medication adherence. For example, study conducted in Kenya concludes that combination therapy associated with poor patient adherence [22]. In contrast, according to study in Nigeria combination therapy produced more patients with controlled blood pressure than mono-therapy [16]. In other way, previous study conducted in Jimma, Ethiopia showed that the number of antihypertensive medications prescribed was not associated with blood pressure control status [18]. Besides, most of previous studies used similar (cross-sectional) study design. As a result, this general prospective cohort study was conducted to assess prescribing pattern of antihypertensive drugs, patient's medication adherence and its determinants among ambulatory hypertension patients.

## Methods

### Study period and setting

This study was conducted over three months period among hypertensive patients who had regular followed up at Jimma university ambulatory cardiac clinic from March 20 to June 20, 2018. Jimma University is the only teaching and specialized hospital in Jimma zone serving the majority of peoples living in Jimma city and its surrounding with catchment population of over 15 million. It is located in Jimma town, Oromia Regional State, Ethiopia, around 352 km southwest of the capital Addis Ababa (http://www.ju.edu.et/jimma university-specialized hospital-Jush).

**Study design:** General prospective cohort study was employed

**Study population**

Patients included in the study were all hypertensive patients with a documented diagnosis and had at least two times medical appointment in the year preceding the study. Participants of ≥ 18 year, having medical appointment every month and complete medical records were included in the study. We excluded pregnant women, patients with neurologic and psychiatric disorder

**Sample size and sampling technique:** The sample size of participants for this study was calculated according to the Cochran formula for sample size calculation for categorical variables. A previous study in Ethiopia reported that the rate of patient medication adherence was 50 % [18] which was used in our sample size calculation. The minimum sample size required for this study was 384 based on standard normal distribution (Z=1.96) with confidence interval of 95% and margin of error of 0.05. Finally, 416 patients were included after adjusting for possible non-respondent rate of 10%. All clinicians who treated hypertensive patients during study period were included and random sampling technique was used for patients.

**Data collection and tool**: questioner and data abstraction format were prepared by reviewing different literatures. To maintain the validity of data collection tool, structured questionnaire was developed translated to local languages (Amharic and Afan Oromo) and then back to English. Pre-test of data collection format was performed on 5% of the sample before conducting the study. Primary data from patients was collected by face-to-face interview and data from all clinicians who treat hypertensive patients during study period was collected by using self-administer questioner. Secondary data such as prescribed drugs, blood pressure measurements and co-morbid illness was collected from patients' chart daily during their visit. In this study hypertensive patients that fulfill the inclusion criteria were selected at the first month of data collection period and BP measurement at this first visit considered as a base line. Then only those patients visited the clinic at first month were consequently followed for the next three months.

Morisky Medication Adherence Scale-8 (MMAS-8) was used to assess the medication taking behavior of patients. Pharmacological Trends Guideline Adherence Evaluation Method was used to measure clinicians' adherence with hypertension treatment guidelines [25]. Recommendations of the JNC -8 (Annex IV) on the detection, evaluation, and treatment of high BP was used as reference guideline to evaluate prescription pattern and clinicians' adherence. process.

**Data processing and analysis:** The data was entered in to computer using Epidata version 3.1and exported to the Statistical Package for Social Science (SPSS) version 21.0. Continuous variables were presented as means (standard deviation). Categorical variables were presented as frequency and percentages. Antihypertensive medicines were categorized according to their therapeutic classes. Switch from one drug to other drug during each visit, last regimen was included in analysis unless changing was done at the last visit. Guideline adherence was measured by proportion of total number of cases (hypertensive patients) treat based on JNC-8 guideline in relation to total number participants. Finally, BP was calculated by taking average of three measurements and categorized controlled and uncontrolled based on guidelines.

Before regression was done multi-collinearity test, and adequacy of cell distribution was checked using chi-square test. Binary logistic regression was performed to determine the effect of each variable on patients' adherence and Variables with P-value less than 0.25 in the bivariate analysis

were then included in a multivariate logistic regression analysis to identify key independent variables influencing patient’s medication adherence. In multivariate analysis, variables with P-value of < 0.05 were considered as statistically significant.

**Operational def[illegible]ions**

**Prescribing pattern:** Types and number of antihypertensive medications prescribed for a given hypertensive patient

**Regular follow up**: Continued care or monitoring of a patient every month after the initial visit or examination.

**Patient adherence:** The extent to which a person’s behavior in taking medication, following a diet, and/or executing lifestyle changes, corresponds with agreed recommendations from a health care provider [16].

**Guideline adherence** is defined as a condition in which the prescribed treatment conforms to treatment recommended in the identified practice guidelines which is expressed as % = (Total number hypertensive patients treat based on JNC-8 guideline/total number of patients) × 100 [23] Based on this calculation >65% concede as complete adherence, 50 – 64.9% medium adherence, and <49.9% will be classified as low adherence [29].

**Controlled blood pressure** defined SBP measurement <140 mmHg and/or DBP of <90 mmHg for age less than 60 years, DM and CKD patients and SBP <150 mmHg and/ or DBP of <90mmHg for age greater than or equal to 60 years old based on average BP measurements, otherwise defined as uncontrolled BP [26].

**Mono therapy:** The use of one antihypertensive drug with only one active ingredient [23]

**Combination therapy:** denoted when patients taking more than one active ingredient, either in one combination or as two different, single pills [23]

**Co-morbid**: It is either the presence of one or more additional disorders (diseases) co-occurring with a primary disease or disorder [28]

**Physically active**: classified as if they reported that they exercise greater than 30 minutes greater than 5 days of the week otherwise they were classified as physically inactive [18].

**Socioeconomic status:** Level I: ETB < 1000; Level II: ETB1001- 2000; Level III: <ETB 2001-3000; Level IV: > 3000ETB; level v: without a well-defined monthly wage

## Results

### Baseline characteristics of study participants

A total of 686 hypertensive patients visited hypertension clinic during study period. Four hundred fifty-nine patients fulfilled inclusion criteria, of these, 416 participants were included in the final analyses. More than half (57.0%) of the participants were males with mean age 56.50 ± 11.96 years (Table 1).

Table 1: Socio-demographic characteristics of hypertensive patients at JUMC, 2018

| Variables | Characteristics | Frequency (%) |
|---|---|---|
| Age (year) | Mean ± SD | 56.50±11.96 |
| | <60 | 220 (52.9) |
| | ≥60 | 196 (47.1) |
| Sex | Male | 237 (57.0) |
| | Female | 179 (43.0) |
| BMI | Mean ± SD | 24.24±2.32 |
| | 18.5-24.9 | 314 (75.5) |
| | 25-29.9 | 89 (21.4) |
| | ≥30 | 13 (3.1) |
| Marital status | Single | 12 (2.9) |
| | Married | 284 (68.3) |
| | Divorced | 55 (13.2) |
| | Widowed | 65 (15.6) |
| Residency | Urban | 274 (65.9) |

| | | |
|---|---|---|
| | Rural | 142 (34.1) |
| Education level | No formal education | 114 (27.4) |
| | Primary education (1-8 grade) | 110 (26.4) |
| | Secondary education (9-12 grade) | 100 (24.0) |
| | Tertiary education (diploma & above) | 92 (22.1) |
| Current occupation | Civil servant | 69 (16.6) |
| | Merchant | 89 (21.4) |
| | Farmer | 74 (17.8) |
| | House wife | 47 (11.3) |
| | Retired | 36 (8.7) |
| | Jobless | 70 (16.8) |
| | Other | 31 (7.5) |
| Monthly income (ETB) | <1000 | 58 (13.9) |
| | 1001-2000 | 68 (16.3) |
| | 2001-3000 | 74 (17.8) |
| | >3000 | 109 (26.2) |
| | Without well-defined monthly income | 107 (25.7) |
| Living status | I live alone | 59 (14.2) |
| | I live with family | 328 (78.8) |
| | Others* | 29 (7.0) |

Others: Drivers, daily laborer, non-governmental organization and private work; others*: live with friends, prison; ETB: Ethiopian Birr

264 (63.5%), of participants have been taking salt with food. More than half, 222 (53.4%) of the participants were physically inactive and 138 (33.2%) were chat chewers. Among co-morbid condition, 104(25.0 %) of the participants had diabetes mellitus (DM), 38(9.1%) and 39(9.4%) had coronary heart disease (CHD) and dyslipidemia, respectively (Table 2).

Table 2: Life style factors and clinical characteristics of hypertensive patients at JUMC, 2018

| Factors | | Frequency (%) |
|---|---|---|

| | | |
|---|---|---|
| Add salt to food | Yes | 264 (63.5) |
| | No | 152 (36.5) |
| Alcohol use | Yes | 103 (24.8) |
| | No | 313 (75.2) |
| Chew chat | Yes | 138 (33.2) |
| | No | 278 (66.8) |
| Cigarette smoking | Never smoked | 303 (72.8) |
| | Ex-smoker | 93 (22.4) |
| | Current smoker | 20 (4.8) |
| Physical activity | Physically active | 194 (46.6) |
| | Physically inactive | 222 (53.4) |
| Drink coffee | Yes | 238 (57.2) |
| | No | 178 (42.8) |
| Use traditional medicine | Yes | 16 (3.8) |
| | No | 400 (96.2) |
| Co morbidity | Hypertension alone | 158 (38.0) |
| | DM | 104 (25.0) |
| | CKD | 16 (3.8) |
| | CHF | 31 (7.5) |
| | CHD | 38 (9.1) |
| | DM and CKD | 16 (3.8) |
| | Dyslipidemia | 39 (9.4) |
| | others | 14 (3.4) |
| Duration on treatment(years) | <1 | 67 (16.1) |
| | 1-5 | 148 (35.6) |
| | >5-10 | 144 (34.6) |
| | >10 | 57 (13.7) |

Others: - Anemia, peripheral neuropathy, lung disease, liver disease, thyroid disorder and human immunodeficiency virus infection

**Prescription patterns of antihypertensive medications**

The overall pattern of antihypertensive agents showed that angiotensin converting enzyme inhibitor (ACEI) was the most frequently prescribed class of antihypertensive drugs 261(63.7%) followed by diuretics 234 (57.1%). Among the study participants, 257 (61.7%) of them had one or more co- medication, of which 98 (23.6%) and 75(18.0%) of them had two and three drugs co- medication, respectively (table 3)

Table 3: Frequency of antihypertensive medicines prescribed for hypertensive patients at JUMC, 2018.

| Classes | Specific drugs | Frequency (%), N=410 |
|---|---|---|
| ACEIs | Enalapril | 261 (63.7) |
| ARBS | Losartan | 28 (6.8) |
| Diuretics | | 234 (57.1) |
| | Hydrochlorothiazide | 203 (49.5) |
| | Furosemide | 26 (6.4) |
| | Furosemide +spironolactone | 5 (1.2) |
| CCBs | | 144 (35.1) |
| | Amlodipine | 131 (31.9) |
| | Nifedipine | 13 (3.2) |
| BBs | | 105 (25.6) |
| | Atenolol | 45 (10.9) |
| | Metoprolol | 47 (11.5) |
| | Propranolol | 13 (3.2) |
| Co-medications | Total number of patients with co-medication | 257 (61.7) |
| | One drug | 61 (14.7) |
| | Two drugs | 98 (23.6) |
| | Three drugs | 75 (18.0) |
| | ≥ four drugs | 23 (5.5) |

ACEIs: angiotensin-converting enzyme inhibitors; ARBs: angiotensin receptor blockers; BBs: βblockers; CCBs: calcium channel blocker

From total participants, 135 (32.5%) of them were on mono-therapy and 275 (66.1%) were on combination therapy. Enalapril was the most frequently prescribed mono therapy 61(14.7%)

followed by hydrochlorothiazide 40(9.6%). Among patients who were on combination therapy, two drugs regimen was prescribed in 46.4% of the hypertensive patients. ACEI +diuretics, 75 (18.0%) was commonly used two drug combination therapy. Three drugs combination accounted for 17(17.1%) of all prescription, of which 35 (8.4%) were on BB+ diuretics+ ACEI followed by CCB+ACEI +diuretic 22(5.3%). Four drugs regimen was prescribed in 11 hypertensive patients, ACEI +diuretics +CCB+BB 7(1.7%) was most frequent prescribed (table 4).

Table 4: Regimens of antihypertensive therapy among hypertensive patients at JUMC, 2018

| Regimens | Specific regimens | Frequency (%) |
|---|---|---|
| Non-pharmacologic | | 6 (1.4) |
| Mono therapy | | 135(32.5) |
| | Enalapril | 61 (14.7) |
| | Amlodipine | 26 (6.2) |
| | Nifedipine | 4 (1.0) |
| | Hydrochlorothiazide | 40 (9.6) |
| | Losartan | 4 (1.0) |
| Dual therapy | | 193 (46.4) |
| | ACEI + Diuretic | 75 (18.0) |
| | CCB + BB | 6 (1.4) |
| | Diuretic + BB | 16 (3.8) |
| | ACEI + CCB | 30 (7.2) |
| | ACEI + BB | 24 (5.8) |
| | Diuretic +ARB | 4 (1.0) |
| | CCB + ARB | 8 (1.9) |
| | CCB+ Diuretics | 26 (6.3) |
| | ARB+BB | 4 (1.0) |

| | | |
|---|---|---|
| Triple therapy | | 71 (17.1) |
| | BB + Diuretic + ACEI | 35 (8.4) |
| | CCB + BB + Diuretic | 7 (1.7) |
| | CCB +ACEI + Diuretic | 22 (5.3) |
| | CCB + BB + ACEI | 1 (0.2) |
| | CCB + BB +ARB | 3 (0.7) |
| | Others* | 3 (0.7) |
| Quadruple | | 11 (2.6) |
| | ACEI + Diuretics + CCB + BB | 7 (1.7) |
| | ARB + BB + CCB + diuretic | 1 (0.2) |
| | Others** | 3 (0.7) |

others*: Enalapril +Furosemide +Spironolactone, Amlodipine +Furosemide + Hydrochlorothiazide; others**: Atenolol+ Enalapril+ Furosemide+ Spironolactone, Amlodipine+ Enalapril+ Furosemide+ Spironolactone

**Clinicians adherence to hypertension treatment guidelines**

Most prescribers perceived that a JNC-8 hypertension treatment guideline was evidence based and helpful in management of patients. However, the overall adherence of clinicians' to JNC-8 guidelines was low, 184 (44.2%) (Figure 1]. From total of patients not treated based on JNC-8 guidelines (N=232), 102(44.0%) patients were not received appropriate first line drugs based on compelling indication and race. In 64(27.6%) patients, combination treatment was not adjusted based on their current BP level (table 5).

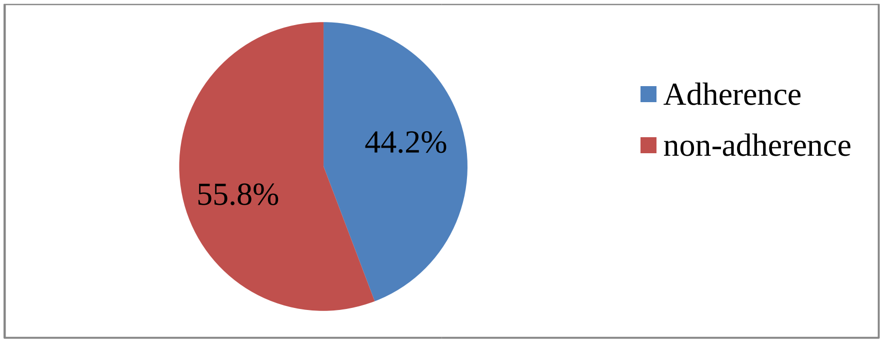

Figure 1: Proportion of clinicians' adherence to JNC-8 hypertension treatment guidelines

Table 5: Common problems observed from prescribed antihypertensive medications to hypertensive patients at JUMC with respect to recommendation of JNC-8 guideline.

| Compliance issue | Frequency N=232 | Percent |
|---|---|---|
| Patients were not received appropriate first line drugs based on compelling indication and race | 102 | 44.0 |
| Combination treatment was not adjusted based on their current blood pressure level | 64 | 27.6 |
| They were not received appropriate dose of medications | 50 | 21.6 |
| Patients were not given both appropriate first line drugs and right dose of medication | 16 | 6.9 |

**Patients adherence to hypertension treatment**

A total of 410 study participants received one or more antihypertensive. From patients' self-response to the eight-item Mo risky medication adherence Scale, Overall, prevalence of anti-hypertensive medication adherence 191 (46.6%) (Figure 2).

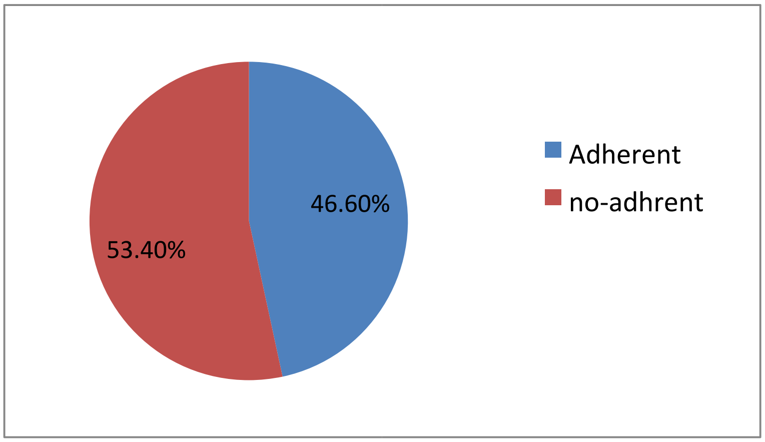


Figure 2: Prevalence of patients' medication adherence at JUMC, 2018

**Determinants of medication non-adherent among hypertensive patients at JUMC, 2018**

In bivariate logistic regression analysis; age, sex, marital status, residency, occupation, monthly income, living status, salt use, chat chewing, physical activity status, concomitant medication, co morbid condition, and duration of treatment were variables with P-value less than 0.25 hence, included in the multivariate logistic regressions. However, current occupation, physical activity status and presence of DM co morbidity were significantly associated with medication non-adherence. Accordingly, respondents who were merchant were 2.46 times (AOR=2.46, CI=1.16-5.23, P=0.020) more likely to be non-adherent than civil servants. Patients who had no regular physical activity were 1.63 times (AOR= 1.63, CI =1.04-2.55, P=0.033) more likely to be nonadherent compared to patients who were physically active. Hypertensive patients with DM comorbidity were 2.54 times (AOR= 2.54, CI=1.28-5.04, P=0.008) more likely to be non-adherent compared to clients with no co morbidity (table 6) .

Table 6: Results of logistic regression analysis for factors associated with medication nonadherence among adult hypertensive patients at JUMC, 2018

| Variables | | Medication adherence | | COR (95%CI) N=410 | P-value | AOR (95%CI) | P- value |
|---|---|---|---|---|---|---|---|
| | | Adherent | not adherent | | | | |
| Age category | <60<br>≥60 | 106<br>85 | 108<br>111 | 1.00<br>1.28(0.87-1.89) | 1.000<br>0.212 | 1.00<br>1.01(0.59-1.72) | 1.000<br>0.966 |
| Sex | Female<br>Male | 78<br>104 | 87<br>132 | 1.27(0.86-1.88)<br>1.00 | 0.234<br>1.000 | 0.92(0.56-1.51)<br>1.00 | 0.742<br>1.000 |
| BMI | 18.5-24.9 25-29.<br>>30 | 141<br>44<br>6 | 170<br>43<br>6 | 1.00<br>0.81(0.50-1.31)<br>0.83(0.26-2.63) | 1.000<br>0.387<br>0.751 | | |
| Marital status | Single<br>Married<br>Divorced | 6<br>140<br>20 | 4<br>140<br>35 | 0.42(0.11-1.62)<br>0.63(0.36- | 0.207<br>0.095<br>0.813 | 0.68(0.14-3.24)<br>0.75(0.38-1.49)<br>1.34(0.58-3.11) | 0.628<br>0.405<br>0.491 |

| | | | | | | | |
|---|---|---|---|---|---|---|---|
| | Widowed | 25 | 40 | 1.09)<br>1.09(0.52-<br>2.30)<br>1.00 | 1.000 | 1.00 | 1.000 |
| Residency | Urban<br>Rural | 134<br>57 | 136<br>83 | 1.00<br>1.44(0.95-<br>2.13) | 1.000<br>0.087 | 1.00<br>1.60(0.83-3.11) | 1.000<br>0.161 |
| Education status | No formal education<br>Primary<br>Secondary<br>Tertiary | 55<br>46<br>46<br>44 | 58<br>62<br>53<br>46 | 1.01(0.58-<br>1.76)<br>1.29(0.74-<br>2.26)<br>1.10(0.62-<br>1.95)<br>1.00 | 0.976<br>0.376<br>0.739<br>1.000 | | |
| Occupation | Civil servant<br>Merchant<br>Farmer<br>House wife<br>Retired<br>Jobless<br>Others | 40<br>30<br>29<br>24<br>12<br>38<br>18 | 27<br>58<br>43<br>22<br>24<br>32<br>13 | 1.00<br>2.86(1.48-<br>5.53)<br>2.20(1.12-<br>4.33)<br>1.36(0.64-<br>2.89)<br>2.66(1.27-<br>6.92)<br>1.25(0.63-<br>2.46)<br>1.07(0.45-<br>2.54) | 1.000<br>0.002<br>0.023<br>0.428<br>0.012<br>0.522<br>0.878 | 1.00<br>2.46(1.16-5.23)<br>1.41(0.52-3.78)<br>1.12(0.43-2.91)<br>2.04(0.66-6.26)<br>0.71(0.26-1.91)<br>0.73(0.26-2.08) | 1.000<br>0.020<br>0.501<br>0.812<br>0.215<br>0.500<br>0.560 |

| | | | | | | | |
|---|---|---|---|---|---|---|---|
| Monthly income (ETB) | <1000 | 21 | 37 | 1.55(0.80-2.98) | 0.194 | 2.01(0.83-4.87) | 0.122 |
| | 1001-2000 | 37 | 30 | 0.71(0.39-1.31) | 0.276 | 0.79(0.36-1.73) | 0.555 |
| | 2001-3000 | 31 | 42 | 1.19(0.65-2.17) | 0.573 | 0.83(0.42-1.64) | 0.593 |
| | Without a well defined | 52 | 53 | 0.89(0.52-1.53) | 0.684 | 0.97(0.43-2.19) | 0.942 |
| | >3000 | 50 | 57 | 1.00 | 1.000 | 1.00 | 1.000 |
| Living status | live alone | 31 | 26 | 1.00 | 1.000 | 1.00 | 1.000 |
| | live with family | 146 | 179 | 1.46(0.83-2.57) | 0.188 | 1.08(0.52-2.23) | 0.841 |
| | others* | 14 | 14 | 1.19(0.48-2.95) | 0.704 | 1.40(0.49-3.98) | 0.528 |
| Salt use | No | 79 | 72 | 1.00 | 1.000 | 1.00 | 1.000 |
| | Yes | 112 | 147 | 1.44(0.96-2.16) | 0.076 | 1.37(0.87-2.17) | 0.180 |
| Alcohol use | No | 145 | 162 | 1.00 | 1.000 | | |
| | Yes | 46 | 57 | 1.11(0.71-1.74) | 0.651 | | |
| Chat chewing | No | 138 | 135 | 1.00 | 1.000 | 1.00 | 1.000 |
| | Yes | 53 | 84 | 1.62(1.07-2.46) | 0.024 | 1.28(0.77-2.13) | 0.338 |
| Cigarette smoking | Never smoked | 142 | 158 | 1.00 | 1.000 | | |
| | Ex-smoker | 40 | 51 | 1.15(0.72-1.84) | 0.572 | | |
| | Current smoker | 9 | 10 | 0.99(0.40-2.53) | 0.998 | | |

| | | | | | | | |
|---|---|---|---|---|---|---|---|
| Physical activity | Inactive<br>Active | 89<br>102 | 130<br>89 | 1.67(1.13-2.48)<br>1.00 | 0.010<br>1.000 | 1.63(1.04-2.55)<br>1.00 | 0.033<br>1.000 |
| Drink coffee | No<br>Yes | 88<br>103 | 89<br>130 | 1.00<br>1.25(0.84-1.85) | 1.000<br>0.268 | | |
| Use traditional medicine | No<br>Yes | 183<br>8 | 211<br>8 | 1.00<br>0.87(0.32-2.36) | 1.000<br>0.780 | | |
| Treatment regimen | Mono therapy<br>Dual therapy<br>Triple therapy<br>Quadruple therapy | 64<br>92<br>31<br>4 | 71<br>101<br>40<br>7 | 1.00<br>0.99(0.64-1.54)<br>1.16(0.65-2.07)<br>1.58(0.44-5.64) | 1.000<br>0.963<br>0.608<br>0.483 | | |
| Drug expensive | No<br>Yes | 147<br>44 | 165<br>54 | 1.00<br>1.09(0.69-1.73) | 1.000<br>0.701 | | |
| Drug unavailable | No<br>yes | 164<br>27 | 184<br>35 | 1.00<br>1.16(0.67-1.99) | 1.000<br>0.603 | | |
| Side effects | No<br>Yes | 170<br>21 | 188<br>31 | 0.75(0.42-1.53)<br>1.00 | 0.339<br>1.000 | | |
| Comedications | One drug<br>Two drugs<br>Three drugs<br>≥four drugs | 31<br>37<br>32<br>13 | 28<br>60<br>43<br>9 | 0.89(0.49-1.62)<br>1.60(0.96-2.68) | 0.708<br>0.074<br>0.317<br>0.410 | 0.52(0.26-1.05)<br>0.91(0.48-1.70)<br>0.65(0.31-1.37)<br>0.36(0.12-1.11) | 0.069<br>0.757<br>0.257<br>0.075 |

| | | | | | | | |
|---|---|---|---|---|---|---|---|
| | No | 78 | 79 | 1.33(0.76-2.31)<br>0.68(0.28-1.69)<br>1.00 | 1.000 | 1.00 | 1.000 |
| Co-morbidities | HTN alone<br>DM<br>CKD<br>CHF<br>CHD<br>DM+CKD<br>Dyslipidemia<br>Others** | 84<br>37<br>11<br>14<br>13<br>11<br>17<br>4 | 72<br>64<br>5<br>17<br>25<br>5<br>21<br>10 | 1.00<br>2.02(1.21-3.37)<br>0.53(0.18-1.59)<br>1.42(0.65-3.01)<br>2.24(1.10-4.70)<br>0.53(0.18-1.60)<br>1.44(0.71-2.94)<br>2.92(0.88-9.69) | 1.000<br>0.007<br>0.260<br>0.378<br>0.032<br>0.260<br>0.315<br>0.081 | 1.00<br>2.54(1.28-5.04)<br>0.51(0.15-1.71)<br>1.14(0.48-2.71)<br>2.13(0.88-5.14)<br>0.51(0.15-1.79)<br>1.14(0.51-2.53)<br>2.81(0.77-10.22) | 1.000<br>0.008<br>0.272<br>0.763<br>0.093<br>0.294<br>0.751<br>0.116 |
| Duration of treatment | <1<br>1-5<br>>5-10<br>>10 | 31<br>60<br>71<br>29 | 32<br>86<br>73<br>28 | 1.07(0.52-2.19)<br>1.49(0.80-2.75)<br>1.07(0.58-1.97)<br>1.00 | 0.855<br>0.208<br>0.841<br>1.000 | 1.03(0.46-2.35)<br>1.54(0.76-3.12)<br>1.16(0.58-2.33)<br>1.00 | 0.936<br>0.231<br>0.683<br>1.000 |

COR= crude odd ratio; AOR= Adjusted odd ratio: Others: Drivers, daily laborer, non-governmental organization and private work; others*: live with friends, prison; Others**: Anemia, peripheral neuropathy, lung disease, liver disease, thyroid disorder and human immunodeficiency virus infection

## Discussion

CCBs and thiazide diuretics are the recommended first line medicines for hypertension for Africa origin as per JNC-8 guidelines. However, from this study clinicians prescribed ACEIs followed by diuretics and CCBs (63.7%, 57.1%, 35.1%), respectively. The use of ACEIs was similar with studies conducted in Serbia (60.57%) and Mexico City (63.8%) [30-31]. Studies performed in Eastern Central Region of Portugal, and rural tertiary hospital in Nigeria showed that thiazide diuretics were the most frequently prescribed class of medication (67%, and 84.9%), respectively [29, 16]. This difference might be due to studies in Portugal and Nigeria used JNC-7 as reference guideline in which is conservative toward the thiazide-type diuretics as initial therapy for most patients without compelling indication.

The result of this study showed that 135 (32.5%) patients were on mono-therapy which was lower than the studies reported from Mexico (72.1%), Canada (56.3%) and Turkey (75.7%) [31-33]. The difference might be due to better health care help to achieve target BP with single medication in these countries. On the other hand, majority of the patients, 275(66.1%) were on combination therapy. The finding was consistent with the study conducted in Kenya (60%) [34]. It was also similar with study conducted Zewditu Memorial Hospital Ethiopia (70.8%) [26]. However, the finding of this study was higher than study conducted in Malaysia (56.7%) [35]. The higher prescription rate of combination therapy in this study might be due to low rate of BP control 42.8% in this study as compared 84.6% in Malaysia.

From the total of 135 (32.5%) patients who were on mono-therapy ACEI, 61 (14.7%) was the most prescribed drug class. The result was closely similar with study conducted in Kenya (20.2%) [34]. However, it was lower than the study conducted in Turkey (30.1%) [33]. The variation might be due to using different standard reference guideline in which European guidelines recommend any class of drug as initial therapy. On contrary, the study conducted in Gondar hospital showed that thiazide diuretics was the most commonly prescribed mono-therapy (60.24%) [36]. This discrepancy might be because of the difference in level guidelines adherence (66.8% prescription based on JNC-8 guidelines) that recommended the use of diuretics for both mono and combination therapy.

Two drugs regimen was prescribed in 46.4 % of the hypertensive patients. ACEI + diuretics, 75 (18.0%) was mostly used two drug combination therapy. The result was closed similar with

study conducted in Kenya (14.5%) [34]. In India, the most frequent prescribed two drug combination was ARB +diuretics [25]. This variation might be due to cost and easily accessibility of ARB in India. On the other hand, the study conducted in Nigeria, CCB + diuretic was the most frequently used two drug combinations (36.6%) [16]. This variation might be suggesting of doctor's preference to use CCBs combination as initial therapy. In additional, in Nigeria majority of the patients were elderly (mean age was 61.5±15.1 years) hence, CCBs preferable for older patients because of isolated SBP more prevalence due to vessels stiffness.

Three drug combination antihypertensive therapies accounted for 17.1% of all prescription, of which BB+ diuretics +ACEI was most frequent. The result was similar with studies conducted in Turkey, Kenya and Gondar hospital, Ethiopia [34, 33, 36]. On the other hand, in India and Nigeria the most frequent prescribed was CCBs+ ACEIs+ Diuretics [25, 16]. This difference might be due to variation in prevalence of the types of co-morbidities. Four drugs regimen was prescribed in 11(2.6%) hypertensive patients, ACEI +diuretics +CCB+BB 7(1.7%) were most frequent prescribed. This was consistent with study conducted in Zewditu Memorial Hospital, Ethiopia [26]. In contrast, study in Nigeria the most frequent quadruple-therapy diuretics +CCBs +ACEIs+ methyldopa [16]. This discrepancy might be because of difference in inclusion criteria, in Nigeria patients with heart failure were excluded from the study; this might be the reason why use of BBs was lower as compared to this study.

The proportion of appropriate use of antihypertensive medications found in this study was 44.2%. The result was slightly higher than study conducted in Zewditu Memorial Hospital, Ethiopia (37.4%) [26] This deference might be due to small in sample size and most of the study participants (65.2%) were without co-morbid condition in Zewditu Memorial Hospital. However, it was lower than studies conducted in South Indian (65%), Malaysia (85.30%), Island (70.4%) and South Africa (51.9%) [21, 23, 37, 29]. This difference might be due to race difference, health care setting that is lack of laboratory and imaging facilities for further screening for target organ damage in our setting. In addition, it might be explained by difference in doctors' profiles since majority of doctors treating hypertensive patients in this study were general practitioner and resident year one (9 and 8 from 23 doctors, respectively).

Medication adherence is the main predictor of treatment success and an effective step in controlling BP. Present study result showed the overall incidence of patients' medication adherence 191 (46.6%). The find was almost similar with WHO report in 2011 in developing countries which was 50% [38]. It was, also closely similar with studies done in Palestinian (45.8%) and Saudi Arabia (46%) [39, 40]. However, it was lower than studies reported in Taiwan (53%), Sweden (63.1%) and china (53.4%) [41, 42, 43]. Variations in the studied populations, better health care and access of health facilities might be contributing to the adherence variation between the studies. Moreover, methods of measurement used to assess medication adherence also could be the reason of variation between the studies. For example, in Taiwan medication adherence was measured using the medication possession ratio (percentage of time that the patient had medication available to them during the follow-up period) whereas, in Sweden adherence measured by using Proportion of Days Covered (PDC) method.

The current finding also lower than the studies conducted in Gondar and Jimma university 64.6% and 61.8% of, respectively [44-45]. This variation might be due to inclusion criteria and adherence measurement scale, in Jimma university inclusion criteria was patients that had at least one year follow up and sample size (280 patients) was lower than this study. In Gondar university adherence was measured using Morisky 4-item Medication Adherence Scale. On the other hand, adherence level reported in this study was higher than the finding from in Ghana and Nigeria (30.3%) and Iran (24%) [46-47]. This difference might be explained, in Ghana and Nigeria hypertensive patients with depression were included in the study hence, psychiatric illnesses contribute for low medication adherence. Similarly, in Iran participants were selected non-random from rural area.

In this study, significant association was observed between patients' current occupation and medication non- adherence. Hypertensive patients who were merchant were 2.46 times more likely to be non-adherent than civil servants. This finding is in line with studies done in Hong Kong China and Black Loin Specialized Hospital, Ethiopia [48-49]. This might be due to forgetfulness when they travel or leave home along with their medicine. In addition, they might be too busy to come to the health facility for their pills and difficulty remembering to take all their medicine on time. Another important factor that influences patient compliance in this study was physically inactivity. Patients who had no regular physical activity were 1.63 times more

likely to be non-adherent compared to patients who were physically active which, was consistence with finding from Iran [47]. The exact mechanism was unclear but might be physical inactive hypertensive patients will have uncontrolled BP since majority of participant (57.2 %) in this study had uncontrolled BP so that clinician may be prescribed more complex treatment regimen, even though, there was no significant association between treatment regimen and patients' adherence level. In addition, due to uncontrolled blood pressure related to physical inactivity, patients might be hopeless to take their medication and adhere with their treatment plan.

In addition, the presence of DM co morbidity was significantly associated with poor antihypertensive medication adherence. Hypertensive patients with DM co morbidity were 2.54 times more likely to be non-adherent compared to with no co-morbidities. Previous study in Taiwan [41] explained that patients with high-risk conditions like DM may be more frequent contacts with medical practitioners, and thus facilitated their medication adherence. However, in this finding the reverse is true, which was similar to a study conducted in South Korea, Hong Kong china, Saudi Arabia and Gondar, Ethiopia [10,48, 40, 44]. Like that of DM co morbidity, hypertensive patient with CHD co-morbidity were almost two time more likely to be nonadherent compared to clients with no co-morbidities, even though it is not statically significant (P=0.093). Possibly this explained by patients with co-morbidities could suffer from serious complications and complex treatment regimens which were unfavorable conditions to adhere their medications. In addition, 27.4% of participants in this study had no formal education, might be difficult to adhere treatment plan and taking on time with complex treatment

## Conclusion

The adherence level of patients to antihypertensive medications was found to be poor. Moreover, being merchant, physical inactivity and presence of DM co-morbidity were factors affecting medication adherence. Prescribing patterns of antihypertensive drugs in this study were inconsistent with JNC- 8 standard treatment guidelines, where ACEIs were the most frequently prescribed class of anti-hypertensive drugs in both mono-therapy and combination therapy and majority of participants were on combination therapy.

## Abbreviation

BP: blood pressure, SBP: systolic blood pressure, DBP: Diastolic blood pressure, CHD: coronary heart disease, DM: diabetes mellitus, CKD: chronic kidney disease, CVD: cardiovascular diseases, IBM: international business machine, JNC-8: eight joint national committee, BB: beta blocker, CCB: calcium channel blocker, ARB: angiotensin receptor blocker, ACEI: angiotensin converting enzyme inhibitor, TD: thiazide diuretic, MMAS-8: Morisky medication adherence scale-8, AOR: adjusted odds ratio, COR: crude odds ratio.

## Declarations

**Acknowledgements**: We thank the participants of the study.

**Funding**: This research was funded by Jimma University, College of Health Sciences. The University has no role in designing, conducting and reporting of the study.

***Availability of data and materials:*** The data sets used and/or analyzed during the current study available from the corresponding author on reasonable request

***Authors' contributions:*** Bekalu Kebede: concept and design, data acquisition, interpretation of data, manuscript preparation; Legese Chelkeba: concept and design, interpretation of data, manuscript preparation, manuscript revision, and manuscript review. Bekalu Dessie: interpretation of data, manuscript preparation, manuscript revision, and manuscript review. All authors read and approved the final manuscript

***Ethics approval and consent to participate:*** The protocol for this study was approved by the Institutional Review Board (IRB), Institute of Health, and Jimma University. Oral consent was obtained from participants before being involved in the study. Confidentiality of data was assured for participants.

***Consent for publication:*** Not applicable.

***Competing interests:*** The authors declare that they have no competing interests

## Refe[illegible]ces

1. Catherine P. Benziger GA. Roth AE. The Global Burden of Disease Study and the Preventable Burden of NCD. Jornal of global heart.2016;11(4):393-397

2. Bromfield S, Muntner P. high blood pressure: the leading global burden of disease risk factor and the need for worldwide prevention programs. 2015;15(3):134–6.

3. Belue R, Okoror TA, Iwelunmor J, Taylor KD, Degboe AN, Agyemang C, et al. Globalization and Health An overview of cardiovascular risk factor burden in sub-Saharan African countries: a socio-cultural perspective. 2009; 12:1–12

4. Kibret KT, Mesfin YM. Prevalence of hypertension in Ethiopia: a systematic metanalysis. Public Health Review. 2015;3-10

5. Molla M. Systematic Reviews of Prevalence and Associated Factors of Hypertension in Ethiopia: Finding the Evidence. 2015;3(4):514–9.

6. Kandala N, Uthman OA. Prevalence of Hypertension in Low- and A Systematic Review and Meta-Analysis. 2015;94(50):1–16.

7. Ibrahim MM, Damasceno A. Hypertension in developing countries. Lancet [Internet]. 2012;380 (9841):611–9.

8. Grassi G, Cifkova R, Laurent S, Narkiewicz K, Redon J, Farsang C, et al. Blood pressure control and cardiovascular risk profile in hypertensive patients from central and eastern European countries. 2011; 32, 218–225

9. Schmieder RE, Mozaffarian D, Véronique L, Emelia J, Jarett D, Michael J et al. End Organ Damage in Hypertension. July 2014; 129(3):1-19.

10. Shin S, Song H, Sang-Kwon Oh, Eob KC, Kim H, Jang S. Effect of antihypertensive medication adherence on hospitalization for cardiovascular disease and mortality in hypertensive patients. Hypertension Research.2013; 36(11):1000-1005.

11. Stevens PE, Levin A. Annals of Internal Medicine Clinical Guideline Evaluation and Management of Chronic Kidney Disease: Synopsis of Kidney Disease. 2013;825–31.

12. Ettehad D, Emdin CA, Kiran A, Anderson SG, Callender T, Emberson J, et al. Blood pressure lowering for prevention of cardiovascular disease and death: a systematic review and meta-analysis. 2015;6736(15):1–11.

13. Jellinger PS, Handelsman Y. AACE 2017 Guidelines American association of clinical endocrinologists and American college of endocrinology guideline for management of dyslipidemia and prevention. 2017;1-87

14. Dennison-himmelfarb C, Handler J, Lackland DT. 2014 Evidence-Based Guideline for the Management of high blood pressure in adults report from the panel members appointed to the eighth Joint National Committee (JNC 8). 2014; 1097:1–14

15. Gebrihet TA, Mesgna KH, Gebregiorgis YS, Kahsay AB, Weldehaweria NB, Weldu G. Awareness, treatment, and control of hypertension is low among adults in Aksum town, northern Ethiopia: A sequential quantitative-qualitative study. 2017;1–16.

16. Busari OA, Oluyonbo R, Fasae AJ, Gabriel OE, Ayodele LM, Agboola SM, et al. Prescribing pattern and utilization of antihypertensive drugs and blood pressure control in adult patients with systemic hypertension in a rural tertiary hospital in Nigeria. 2014;2(6):144–9.

17. Parati G, Omboni S, Compare A, Grossi E, Callus E, Venco A, et al. Blood pressure control and treatment adherence in hypertensive patients with metabolic syndrome: protocol of a randomized controlled study based on home blood pressure telemonitoring vs. conventional management and assessment of psychological determinants of adherence. 2013;1–11.

18. Asgedom SW, Gudina EK, Desse TA. Assessment of Blood Pressure Control among Hypertensive Patients in Southwest Ethiopia. 2016;1–12.

19. Karaku[illegible]. Factors affecting medication adherence in patients with hypertension. 2012;118–26.

20. Rolnick SJ, Pawloski PA, Hedblom BD. Patient Characteristics Associated with Medication Adherence. 2013;1-33

21. Datta S. Utilization Study of Antihypertensives in a South Indian Tertiary Care Teaching Hospital and Adherence to Standard Treatment Guidelines. 2017;33–7.

22. Ernest M, Moses M, Beth M, Muiruri N, Cheptum J, Maingi T. Level of blood pressure control among hypertensive patients on follow-up in a Regional Referral Hospital in Central Kenya. 2014; 8688:1–10

23. Hassan Y, Abdul HA. Physician adherence to hypertension treatment guidelines and drug acquisition costs of antihypertensive drugs at the cardiac clinic: a pilot study. 2012;101–8.

24. Gudina EK, Asgedom SW, Desse TA. Prevalence of hypertension and its risk factors southwest Ethiopia: a hospital-based cross-sectional survey. 2013;111-117

25. Romday R, Gupta A, Bhambani P. assessment of antihypertensive drug prescription patterns and adherence to joint national committee-8 hypertension treatment guidelines among hypertensive patients attending a tertiary teaching hospital. 2016;4(12):5125-33.

26. Degarege Y. Assessment of the management of hypertension among hypertensive patients on follow-up at cardiac clinic of Zewditu Memorial Hospital , Addis Ababa , Ethiopia 2017;1-53

27. Pittrow D, Kirch W, Bramlage P, Lehnert H, Höfler M, Unger T, et al. Patterns of antihypertensive drug utilization in primary care. 2005;1-11

28. Morgado MP, Rolo SA, Pereira L, Castelo-branco M. Blood pressure control and antihypertensive pharmacotherapy patterns in a hypertensive population of Eastern Central Region of Portugal. 2010;10(1):349

29. Makgatho S. Adherence of doctors to a clinical guideline for hypertension in Bojanala district, North-West Province, South Africa. 2015; 6–11.

30. Tomas A, Tomi Z, Milijaševi B, Ban M, Horvat O, Vukmirovi S, et al. Patterns of prescription antihypertensive drug utilization and adherence to treatment guidelines in the city of Novi. 2016;73(6):531–7.

31. Carvajal A, Juan M, Alba-leonel A, Carvajal A, Fierro I. Prescription patterns of antihypertensives in a community health centre in Mexico City. 2017;1-7

32. Beaulieu M, Dufresne CL, Leblanc D. Treating hypertension Are the right drugs given to the right patients? 1998; 44:294-302

33. Abaci A, Kozan O, Oguz A. Prescribing pattern of antihypertensive drugs in primary care units in Turkey: results from the Turksaha study. 2007;397–402

34. Mbui JM, Oluka MN, Guantai EM, Sinei KA, Baker A, Jande M, et al. Expert Review of Clinical Pharmacology Prescription patterns and adequacy of blood pressure control among adult hypertensive patients in Kenya; findings and implications. 2017;10(11):1263–71.

35. Ramli AS, Uk M, Mmed MM, Medicine F, Ng KK, Family M, et al. Original Article prescribing of antihypertensive agent in public primary care clinics, is it in accordance with current evidence? 2010;5(1):36–40

36. Befekadu T. Antihypertensive drug prescription patterns and their impact on outcome of blood pressure in Ethiopia: a hospital-based cross-sectional study. 2017; 6:29-35

37. Theodorou M, Stafylas P, Kourlaba G, Kaitelidou D, Maniadakis N, Papademetriou V. Physicians' Perceptions and Adherence to Guidelines for the Management of Hypertension: A National, Multicenter, Prospective Study. 2012;1-12

38. Marie T, Jennifer K. Medication Adherence: WHO Cares? 2011;86 (4):304-314

39. Al-ramahi RÕ. Adherence to medications and associated factors: A cross-sectional study among Palestinian hypertensive patients. 2015;125–32

40. Khayyat SM, Khayyat SMS, Alhazmi RSH, Mohamed MA, Hadi MA. Predictors of medication adherence and blood pressure control among Saudi Hypertensive Patients Attending Primary Care Clinics: A Cross-Sectional Study. 2017;1–12.

41. Lee C, Huang C, Shih H, Huang K. Factors influencing antihypertensive medication compliance in Taiwan: a nationwide population-based study. 2012:1-8

42. Hedna K, Hakkarainen KM, Gyllensten H, Jönsson AK. Adherence to Antihypertensive Therapy and Elevated Blood Pressure: Should We Consider the Use Medications? 2015;1–14.

43. Li YT, Wang HHX, Liu KQL, Lee GKY, Chan WM, Griffiths SM, et al. medication Adherence and blood pressure control among hypertensive patients with coexisting long-term conditions in primary care settings. 2016;95 (20):1–10.

44. Ambaw AD, Alemie GA, Yohannes SMW, Mengesha ZB. Adherence to antihypertensive treatment and associated factors among patients on follow up at University of Gondar Hospital Northwest Ethiopia. BMC Public Health [Internet]. 2012;12(1):1.

45. Asgedom SW, Atey TM, Desse TA. Antihypertensive medication adherence and associated factors among adult hypertensive patients at Jimma University Specialized Hospital, southwest Ethiopia. 2018;1–8

46. Boima V, Ademola AD, Odusola AO, Agyekum F, Nwafor CE, Cole H, et al. Factors associated with medication nonadherence among hypertensive in Ghana and Nigeria. 2015;1-9

47. Kamran A, S SA, Biria M, Malepour A, Heydari H. Determinants of patient's adherence to hypertension medications: application of health belief model among rural patients. 2013;4(6):2-7

48. Kang CD, Tsang PPM, Li WTL, Wang HHX, Liu KQL, Grif SM, et al. Determinants of medication adherence and blood pressure control among hypertensive patients in Hong Kong: A cross-sectional study. 2015; 182:250–7

49. Habtamu A, Mesfin A, Tadese A. assessment of adherence to hypertension management and its influencing factors among hypertensive patients attending Black Lion hospital chronic follow up unit, Addis Ababa, Ethiopia. 2013;4(3):1086–95.
