# Supplementary material for: Clinicians’ prescribing pattern, rate of patients’ medication adherence and its determinants among adult hypertensive patients at Jimma University Medical Center: Prospective cohort study
Source: PLoS One. 2021 Nov 15;16(11):e0259421. doi: 10.1371/journal.pone.0259421 (PMC8592482; doi:10.1371/journal.pone.0259421)
Supplement: Attachment — Submitted filename: PONE-D-20-38133_R1 PLOS ONE Article SAO review 20.07.2021final.pdf [file pone.0259421.s006.pdf]

PLOS ONE

Clinicians' Prescribing Pattern, Rate of Patients' Medication Adherence and Its Determinants among Adult Hypertensive Patients at Jimma University Medical Center: Prospective Cohort Study

--Manuscript Draft--

Manuscript Number: PONE-D-20-38133R1

Article Type: Research Article

Full Title: Clinicians' Prescribing Pattern, Rate of Patients' Medication Adherence and Its Determinants among Adult Hypertensive Patients at Jimma University Medical Center: Prospective Cohort Study

Short Title: Clinician's Prescribing Pattern, Rate of Patients' Medication Adherence and Its Determinants

Corresponding Author: Bekalu Kebede simegn, MSc
Debremarkos Uiversity
Debre Markos, Amhara ETHIOPIA

Keywords: Prescribing Pattern, Hypertension, Blood Pressure Control, Guidelines, Adherence

Abstract:

Background: Despite the goals for management of hypertension are well defined, effective therapies are widely available, co[illegible] of hypertension is poor and hypertension related cardiovascular diseases are still high in most health care setting. Therefore, this study aimed to determine clinician's prescribing pattern, Patients' Medication Adherence and Its Determinants among ambulatory hypertensive patients at Jimma University Medical Center.

Method: General prospective cohort study was conducted among hypertensive patients who had regular follow up at Jimma university ambulatory cardiac clinic from March 20 to June 20, 2018. Patients' specific data was collected using structured data collection tool directly from patients and their medical chart and from clinicians through self-administered questionnaire. Data was analyzed using SPSS version 21.0. Bivariate and multivariable logistic regression analyses were done to identify key independent variables influencing patients' adherence. P-Values of less than 0.05 were considered as statically significant.

Results: From total of ~~e~~ 416 patients, 237(57.0%) were males with mean age of 56.50 ± 11.96 years. Angiotensin converting enzyme inhibitor was the most frequently prescribed, 261(63.7%) and majority of patients, 275 (66.1%) were on combination therapy. The rate of patients' medication adherence and clinicians' guidelines adherence were 46.6%, and 44.2%, respectively. Patient with occupation of Merchant (P=0.020), physical inactivity (P=0.033) and diabetes mellitus co-morbidity (P=0.008) were significantly associated with medication non-adherence.

Conclusion: The rate of medication adherence among hypertensive patients was poor and. physicians were not adhering to standard treatment guidelines. Angiotensin converting enzyme inhibitor was the most f[illegible]ently prescribed class of drugs. Effective education for patients to improve medication adherence and regular training for prescriber on practical treatment guidelines is recommended to keep them up-to-date with current trends of hypertension treatment and for better treatment outcome

Order of Authors:
Bekalu Kebede simegn, MSc
Bekalu Dessie Alamirew
Legese chelkeba

Response to Reviewers: First, we would like to appreciate the editor and reviewers for giving us another round of invaluable comments so as to revise our manuscript accordingly. We have gone through all the comments given by the reviewers and revised the manuscript point by point.

Additional Information:

Question	Response
Financial Disclosure	This research was funded by Jimma University, College of Health Sciences.

Powered by Editorial Manager® and ProduXion Manager® from Aries Systems Corporation

Enter a financial disclosure statement that describes the sources of funding for the work included in this submission. Review the submission guidelines for detailed requirements. View published research articles from *PLOS ONE* for specific examples.<br><br>This statement is required for submission and **will appear in the published article** if the submission is accepted. Please make sure it is accurate.<br><br>**Unfunded studies**<br>Enter: *The author(s) received no specific funding for this work.*<br><br>**Funded studies**<br>Enter a statement with the following details:<br>• Initials of the authors who received each award<br>• Grant numbers awarded to each author<br>• The full name of each funder<br>• URL of each funder website<br>• Did the sponsors or funders play any role in the study design, data collection and analysis, decision to publish, or preparation of the manuscript?<br>• **NO** - Include this sentence at the end of your statement: *The funders had no role in study design, data collection and analysis, decision to publish, or preparation of the manuscript.*<br>• **YES** - Specify the role(s) played.<br><br>* typeset	The University has no role in designing, conducting and reporting of the study.
**Competing Interests**<br><br>Use the instructions below to enter a competing interest statement for this submission. On behalf of all authors, disclose any competing interests that could be perceived to bias this work—acknowledging all financial support and any other relevant financial or non-financial competing interests.<br><br>This statement **will appear in the published article** if the submission is accepted. Please make sure it is accurate. View published research articles	The authors declared that no competing interests exist.

<table>
<tr>
<td>from PLOS ONE for specific examples.

NO authors have competing interests

Enter: The authors have declared that no competing interests exist.

Authors with competing interests

Enter competing interest details beginning with this statement:

I have read the journal's policy and the authors of this manuscript have the following competing interests: [insert competing interests here]

* typeset</td>
<td></td>
</tr>
<tr>
<td>Ethics Statement

Enter an ethics statement for this submission. This statement is required if the study involved:

• Human participants
• Human specimens or tissue
• Vertebrate animals or cephalopods
• Vertebrate embryos or tissues
• Field research

Write "N/A" if the submission does not require an ethics statement.

General guidance is provided below. Consult the submission guidelines for detailed instructions. Make sure that all information entered here is included in the Methods section of the manuscript.</td>
<td>Ethics approval and consent to participate: This study was approved by the Ethical Review Board of School of Pharmacy, Institute of Health Science Jimma University (Ref.No IHRPGD/203/18). First, each participant was asked orally whether they were voluntary or not to participate in this study after a detailed explanation of the objective of the study, procedures of selection, and assurance of confidentiality. An independent data collection supervisor acted as witness for voluntary informed decision making of participants to take part in the study. Written informed consent was waived since the study did not involve any procedure and pres[illegible] no damage to patients as approved by the ethical review board committee of the Board of School of Pharmacy, Institute of Health Science Jimma University. Their names were not registered to minimize social desirability bias and enhance anonymity. They were not forced to participate or receive any monetary incentive and it was solely voluntary based.</td>
</tr>
</table>

**Format for specific study types**<br><br>**Human Subject Research (involving human participants and/or tissue)**<br>• Give the name of the institutional review board or ethics committee that approved the study<br>• Include the approval number and/or a statement indicating approval of this research<br>• Indicate the form of consent obtained (written/oral) or the reason that consent was not obtained (e.g. the data were analyzed anonymously)<br><br>**Animal Research (involving vertebrate animals, embryos or tissues)**<br>• Provide the name of the Institutional Animal Care and Use Committee (IACUC) or other relevant ethics board that reviewed the study protocol, and indicate whether they approved this research or granted a formal waiver of ethical approval<br>• Include an approval number if one was obtained<br>• If the study involved *non-human primates*, add *additional details* about animal welfare and steps taken to ameliorate suffering<br>• If anesthesia, euthanasia, or any kind of animal sacrifice is part of the study, include briefly which substances and/or methods were applied<br><br>**Field Research**<br><br>Include the following details if this study involves the collection of plant, animal, or other materials from a natural setting:<br>• Field permit number<br>• Name of the institution or relevant body that granted permission	
**Data Availability**<br><br>Authors are required to make all data underlying the findings described fully available, without restriction, and from the time of publication. PLOS allows rare exceptions to address legal and ethical concerns. See the PLOS Data Policy and FAQ for detailed information.	No - some restrictions will apply

A Data Availability Statement describing where the data can be found is required at submission. Your answers to this question constitute the Data Availability Statement and **will be published in the article**, if accepted.<br><br>**Important:** Stating 'data available on request from the author' is not sufficient. If your data are only available upon request, select 'No' for the first question and explain your exceptional situation in the text box.<br><br>Do the authors confirm that all data underlying the findings described in their manuscript are fully available without restriction?	
**Describe where the data may be found in full sentences. If you are copying our sample text, replace any instances of XXX with the appropriate details.**<br><br>• If the data are **held or will be held in a public repository**, include URLs, accession numbers or DOIs. If this information will only be available after acceptance, indicate this by ticking the box below. For example: *All XXX files are available from the XXX database (accession number(s) XXX, XXX.*).<br>• If the data are all contained **within the manuscript and/or Supporting Information files**, enter the following: *All relevant data are within the manuscript and its Supporting Information files.*<br>• If neither of these applies but you are able to provide **details of access elsewhere**, with or without limitations, please do so. For example:<br><br>*Data cannot be shared publicly because of [XXX]. Data are available from the XXX Institutional Data Access / Ethics Committee (contact via XXX) for researchers who meet the criteria for access to confidential data.*<br><br>*The data underlying the results presented in the study are available from (include the name of the third party*	All relevant data are within the manuscript and its Supporting Information files

Powered by Editorial Manager® and ProduXion Manager® from Aries Systems Corporation

and contact information or URL).<br>• This text is appropriate if the data are owned by a third party and authors do not have permission to share the data.<br><br>* typeset	
Additional data availability information:	Tick here if the URLs/accession numbers/DOIs will be available only after acceptance of the manuscript for publication so that we can ensure their inclusion before publication.

Powered by Editorial Manager® and ProduXion Manager® from Aries Systems Corporation

Manuscript Click here to access/download;Manuscript;Manuscript.docx

Clinicians' Prescribing Pattern, Rate of Patients' Medication Adherence and Its Determinants among Adult Hypertensive Patients at Jimma University Medical Center: Prospective Cohort Study

Bekalu Kebede,MSc[1], Legese Chelkeba, PhD[2], Bekalu Dessie,MSc[1]

[1] Department of Pharmacy, College of Health Sciences, Debre Markos University, Debre Markos, Ethiopia

[2]Department of Clinical Pharmacy, School of Pharmacy, College Health Sciences, Addis Ababa University, Jimma, Ethiopia

Corresponding author: Bekalu Kebede

Clinical Pharmacist, College of Health, Department of pharmacy, Debre Markos University, Debre Markos, Ethiopia

Phone: +251-921-286-140

Email: bekalukebede19@gmail.com

Abstract

Background: Despite the goals of hypertension treatment are well defined and effective therapies are widely available, control of hypertension remains poor, and hypertension-related cardiovascular diseases are still high in most health care settings. Therefore, this study aimed to determine clinicians' prescribing patterns, patients' medication adherence, and its determinants among ambulatory hypertensive patients at Jimma University Medical Center.

Methods: A general prospective cohort study was conducted among hypertensive patients who had regular follow-up at Jimma university ambulatory cardiac clinic from March 20, 2018, to June 20, 2018. Patients' specific data was collected with a face-to-face interview and from their medical chart. Clinicians' related data were collected through a self-administered questionnaire. Data were analyzed using SPSS version 21.0. Bivariate and multivariable logistic regression analyses were done to identify key independent variables influencing patients' adherence. P-Values of less than 0.05 were considered statically significant.

Results: From the total of 416 patients, 237(57.0%) were males with a mean age of 56.50 ± 11.96 years. Angiotensin-converting enzyme inhibitors were the most frequently prescribed class of antihypertensives accounting for 261(63.7%) prescriptions. The majority of patients, 275 (66.1%) were on combination therapy. The rate of patients' medication adherence and clinicians' guidelines adherence was 46.6%, and 44.2%, respectively. Patients with merchant occupation (P=0.020), physical inactivity (P=0.033), and diabetes mellitus co-morbidity (P=0.008) were significantly associated with medication non-adherence.

Conclusion: The rate of medication adherence among hypertensive patients was poor and physicians were not adhering to standard treatment guidelines. Angiotensin-converting enzyme inhibitors were the most frequently prescribed class of drugs. Effective education for patients to improve medication adherence and regular training for prescribers on practical treatment guidelines is recommended to keep them up-to-date with current trends of hypertension treatment and for better treatment outcomes.

Keywords: Prescribing Pattern, Blood Pressure Control, Guidelines, Adherence

Introduction

Hypertension [illegible]gnificant public health challenge worldwide due to its high prevalence and concomitant risks of cardiovascular and kidney diseases [1]. The World Health Organization (WHO) has estimated that high blood pressure (BP) is a major public health issue and causes one in every eight deaths, and is the third leading silent killer in the world [2].

Almost three-quarters of people with hypertension (639 million people) live in countries with limited health resources [1, 2]. In Sub- Saharan Africa, it is a major independent risk factor for heart failure, stroke, and kidney failure. These complications result due to a low rate of hypertension diagnosis, suboptimal BP control, high morbidity and mortality, and low resources in health care settings [3]. A systematic review and meta-analysis study done in Ethiopia in 2015 showed that the prevalence of hypertension was estimated to be 19.6 % [4]. Another systemic review and meta-analysis conducted in Ethiopia in 2015 reported that the prevalence of hypertension was between 20% and 30% [5].

Although hypertension is a preventable and modifiable risk factor for cardiovascular diseases (CVD), the prevention and control of hypertension have not yet received attention in many developing countries [6,7]. It accounts for 40.6% of deaths due to all coronary heart disease and 38.5% of deaths due to stroke [8]. It is also the second-leading cause of end-stage renal disease (ESRD) behind diabetes mellitus (DM [9].

Guideline adherence is defined as a condition in which the prescribed treatment obeys treatments recommended in the identified practice guidelines [10-12]. Hypertension guidelines are evidence-based and are usually dictated by randomized controlled trial data and observational studies. Published guidelines aid in clinical decision making, decrease practice variations, guide correctness, and measure the quality of health care [13-14]. The Eighth Joint National Committee (JNC-8) on detection, evaluation, and treatment of BP is most the commonly used guideline and it is considered as a "gold standard" consensus guideline for the management of hypertension [15]. This guideline was used as a reference standard for this study because awareness and accessibility of guidelines were initial criteria to evaluate the status of clinicians' adherence to hypertension treatment guidelines. Besides, Ethiopia has not its hypertension treatment guideline.

Therefore, JNC-8 guideline is widely used by both clinicians and clinical pharmacists in Ethiopia [16]. However, clinical practice guidelines do not consistently change clinicians' behavior, control of high blood pressure remains suboptimal, so creating the need to evaluate adherence to guidelines and its impact on BP control [10].

Many studies conducted in the past to explore the causes of suboptimal BP control focused on patient factors such as socio-demographic, medical profile, and patient's treatment compliance [17-20]. However, prescribing patterns and clinicians' adherence to guidelines are also important factors affecting hypertension treatment outcomes [21-24].

Several studies were conducted worldwide using either prescription or drug dispensing data to evaluate prescription patterns and clinicians 'adherence to hypertension treatment guidelines [25-27]. But few data are available in Ethiopia. Also, among previous studies, there were conflicting results between prescribing pattern and patient's medication adherence. For example, a study conducted in Kenya concludes that combination therapies were associated with poor patient adherence [22]. In contrast, according to a study conducted in Nigeria combination therapies were associated with good blood pressure controlled than mono-therapy [16]. A previous study conducted at Jimma University, Ethiopia showed that the number of antihypertensive medications prescribed was not associated with blood pressure control status [18]. Besides, most previous studies used a similar study design(cross-sectional). As a result, a general prospective cohort study was conducted among ambulatory hypertension patients to determine prescribing patterns of antihypertensive drugs, patient's medication adherence, and its determina[illegible].

Methods

Study period and setting

This study was conducted over a three-month period among hypertensive patients who had regular followed up every month at Jimma university ambulatory cardiac clinic from March 20, 2018, to June 20, 2018. Jimma University is the only teaching and specialized hospital in Jimma zone. It provides service for over 15 million peoples living in Jimma city and its surroundings It

is located in Jimma town, Oromia Regional State, Ethiopia, 352 km southwest of Addis Ababa, the capital city of Ethiopia (18).

Study design: a general prospective cohort follow-up study was employed

Study population; All hypertensive patients with age ≥ 18 years, with a documented diagnosis, had at least two times medical appointments before the study period, having a medical appointment every month and complete medical records were included in the study. We excluded pregnant women, patients with confirmed neurologic and psychiatric disorders with their respective specialists since it was challenging to assess medication adherence statuses in acutely ill patients with similar tools like that of other study participants.

Sample size and sampling technique: The sample size for this study was calculated using Cochran single proportion formula. A previous study conducted in Ethiopia reported that the rate of patient medication adherence was 50 % [18]. Based on this formula minimum 384 sample size was calculated using a standard normal distribution (Z=1.96) with a confidence interval of 95% and a margin of error of 0.05. After adjusting 10% non-respondent rate, 416 samples were used in this study. All clinicians who treated hypertensive patients during the study period were included and a random sampling technique was used for patients.

Data collection and tool: questionnaire and data abstraction format were prepared by reviewing different kinds of literature. To maintain the validity of the data collection tool, a structured questionnaire was developed translated to local languages (Amharic and Afan Oromo) by native speakers of the respective languages and then back translated to the English version. Pre-test of data collection format was performed on 5% of the sample. Primary data from patients were collected by face-to-face interview and data from all clinicians who treat hypertensive patients during the study period was collected by using a self-administer questionnaire. Secondary data such as prescribed drugs, blood pressure measurements, and co-morbid illness was collected from patients' chart daily during their visit. At Jimma University Medical Center, initial and subsequent BP typically measured using the cuff sphygmomanometer and digital sphygmomanometer on the left arm, at the level of the heart, in a sitting position, and at rest for at least 5 minutes. In this study hypertensive patients that fulfilled the inclusion criteria were selected at the first month of the data collection period and BP measurement at this first visit was

considered as a baseline. Then only those patients selected at the first month were consequently followed every month for the next three months.

Morisky Medication Adherence Scale-8 (MMAS-8) was used to assess the patients' medication adherence status. The MMAS-8 total score was calculated by summing the values from all 8 items, with reverse coding when necessary. A score of <2 (out of a full range of 8) was considered as good medication adherence, otherwise considered as poor adherence [19].

Pharmacological Trends Guideline Adherence Evaluation Method was used to measure clinicians' adherence with hypertension treatment guidelines [25]. A recommendation of the JNC-8 on the detection, evaluation, and treatment of high BP was used as a reference guideline to evaluate prescription patterns and clinicians' adherence to the guidelines.

Data processing and analysis: The data were entered using Epidata version 3.1 and exported to the Statistical Package for Social Science (SPSS) version 21.0. Continuous variables were presented as means (standard deviation). Categorical variables were presented as frequency and percentages. Antihypertensive medicines were categorized according to their therapeutic classes. Switch from one drug to another drug during each visit, the last regimen was included in the analysis unless changing was done at the last visit.

Clinicians' guideline adherence was measured by the proportion of the total number of cases (hypertensive patients) treat based on the JNC-8 guideline with the total number of participants. Guideline adherence was expressed as % = (Total number hypertensive patients treat based on JNC-8 guideline divide by the total number of patients) × 100 [23]. Based on this calculation >65% conceder as complete adherence, 50 – 64.9% medium adherence, and <49.9% will be classified as low adherence [29]. Finally, BP was calculated by taking an average of three measurements and categorized controlled and uncontrolled based on guidelines.

Before regression was done a multi-collinearity test, and adequacy of cell distribution was checked using chi-square test. Binary logistic regression was performed to determine the effect of each variable on patients' adherence and variables with a P-value less than 0.25 in the bivariate analysis were then included in a multivariate logistic regression analysis to identify key independent variables influencing patient's medication adherence. In multivariate analysis, variables with P- a value of < 0.05 were considered statistically significant.

Ethics approval and consent to participate: This study was approved by the Ethical Review Board of School of Pharmacy, Institute of Health Science Jimma University (Ref.No IHRPGD/203/18). First, each participant was asked orally whether they were voluntary or not to participate in this study after a detailed explanation of the objective of the study, procedures of selection, and assurance of confidentiality. An independent data collection supervisor acted as witness for voluntary informed decision making of participants to take part in the study. Written informed consent was waived since the study did not involve any procedure and present no damage to patients as approved by the ethical review board committee of the Board of School of Pharmacy, Institute of Health Science Jimma University. Their names were not registered to minimize social desirability bias and enhance anonymity. They were not forced to participate or receive any monetary incentive and it was solely voluntary based.

Results

Baseline characteristics of study participants

A total of 686 hypertensive patients visited the hypertension clinic during the study period. Four hundred fifty-nine patients fulfilled inclusion criteria, of these, 416 participants were included in the final analyses (Fig 1). More than half (57.0%) of the participants were males with mean age of 56.50 ± 11.96 years (Table 1).

Figure 1: Hypertension patient selection flow chart at Jimma University Medical Center from March 20, 2018, to June 20, 2018

Table 1: Socio-demographic characteristics of hypertensive patients at JUMC, 2018

**Variables**	**Characteristics**	**Frequency (%)**
**Age (year)**	Mean ± SD	56.50±11.96
	<60	220(52.9)
	≥60	196(47.1)
**Sex**	Male	237 (57.0)
	Female	179 (43.0)

**BMI**	Mean ± SD	24.24±2.32
	18.5-24.9	314 (75.5)
	25-29.9	89 (21.4)
	≥30	13 (3.1)
**Marital status**	Single	12 (2.9)
	Married	284 (68.3)
	Divorced	55 (13.2)
	Widowed	65 (15.6)
**Residency**	Urban	274 (65.9)
	Rural	142 (34.1)
**Education level**	No formal education	114 (27.4)
	Primary education (1-8 grade)	110 (26.4)
	Secondary education (9-12 grade)	100 (24.0)
	Tertiary education (diploma & above)	92 (22.1)
**Current occupation**	Civil servant	69 (16.6)
	Merchant	89 (21.4)
	Farmer	74 (17.8)
	Housewife	47 (11.3)
	Retired	36 (8.7)
	Jobless	70 (16.8)
	Other	31 (7.5)
**Monthly income (ETB)**	<1000	58 (13.9)
	1001-2000	68 (16.3)
	2001-3000	74 (17.8)
	>3000	109 (26.2)
	Without unknown monthly income	107 (25.7)
**Living status**	I live alone	59 (14.2)
	I live with family	328 (78.8)
	Others*	29 (7.0)

Others: Drivers, daily laborer, non-governmental organization and private work; others*: live with friends, prison; ETB: Ethiopian Birr

Two hundred sixty-four (63.5%) of participants have been taking salt with food. More than half (53.4%) of the participants were physically inactive and 138 (33.2%) patients were khat chewers. Among co-morbid conditions, 104(25.0 %) of the participants had diabetes mellitus (DM), 38(9.1%) and 39(9.4%) had coronary heart disease (CHD) and dyslipidemia, respectively (Table 2).

Table 2: Lifestyle factors and clinical characteristics of hypertensive patients at JUMC, 2018

**Factors**	**Categories**	**Frequency (%)**
**Add salt to food**	Yes	264 (63.5)
	No	152 (36.5)
**Alcohol use**	Yes	103 (24.8)
	No	313 (75.2)
**Chew khat**	Yes	138 (33.2)
	No	278 (66.8)
**Cigarette smoking**	Never smoked	303 (72.8)
	Ex-smoker	93 (22.4)
	Current smoker	20 (4.8)
**Physical activity**	Physically active	194 (46.6)
	Physically inactive	222 (53.4)
**Drink coffee**	Yes	238 (57.2)
	No	178 (42.8)
**Use traditional medicine**	Yes	16 (3.8)
	No	400 (96.2)
**Co morbidity**	Hypertension alone	158 (38.0)
	DM	104 (25.0)
	CKD	16 (3.8)
	CHF	31 (7.5)
	CHD	38 (9.1)
	DM and CKD	16 (3.8)
	Dyslipidemia	39 (9.4)

	others	14 (3.4)
**Duration on treatment(years)**	<1	67 (16.1)
	1-5	148 (35.6)
	>5-10	144 (34.6)
	>10	57 (13.7)

Others:- Anemia, peripheral neuropathy, lung disease, liver disease, thyroid disorder, and human immunodeficiency virus infection

Prescription patterns of antihypertensive medications

The overall pattern of antihypertensive agents showed that angiotensin-converting enzyme inhibitor (ACEI) was the most frequently prescribed class of antihypertensive drugs 261(63.7%) followed by diuretics 234 (57.1%). Among the study participants, 257 (61.7%) of them had one or more concomitant medications, of which 98 (23.6%) and 75(18.0%) of them had two and three drugs concomitant medications, respectively (table 3)

Table 3: Frequency of antihypertensive medicines prescribed for hypertensive patients at JUMC, 2018.

Classes	Specific drugs	Frequency (%), N=410
**ACEIs**	Enalapril	261 (63.7)
**ARBS**	Losartan	28 (6.8)
**Diuretics**		234 (57.1)
	Hydrochlorothiazide	203 (49.5)
	Furosemide	26 (6.4)
	Furosemide +spironolactone	5 (1.2)
**CCBs**		144 (35.1)
	Amlodipine	131 (31.9)
	Nifedipine	13 (3.2)
**BBs**		105 (25.6)
	Atenolol	45 (10.9)
	Metoprolol	47 (11.5)
	Propranolol	13 (3.2)

**Co-medications**	Total number of patients with co-medication	257 (61.7)
	One drug<br>Two drugs<br>Three drugs<br>≥ Four drugs	61 (14.7)<br>98 (23.6)<br>75 (18.0)<br>23 (5.5)

ACEIs: Angiotensin-converting enzyme inhibitors; ARBs: angiotensin receptor blockers; BBs: βblockers; CCBs: calcium channel blocker

From the total participants, 135 (32.5%) of them were on mono-therapy and 275 (66.1%) were on combination therapy. Enalapril was the most frequently prescribed monotherapy 61(14.7%) followed by hydrochlorothiazide 40(9.6%). Among patients who were on combination therapy, two-drug regimens were prescribed in 46.4% of the hypertensive patients. ACEI +diuretics, 75 (18.0%) was commonly used two-drug combination therapy. Three drugs combination accounted for 17(17.1%) of all prescriptions, of which 35 (8.4%) were on BB+ diuretics+ ACEI followed by CCB+ACEI +diuretic 22(5.3%). Four drugs regimen was prescribed in 11 hypertensive patients, ACEI +diuretics +CCB+BB 7(1.7%) was most frequent prescribed (Table 4).

Table 4: Regimens of antihypertensive therapy among hypertensive patients at JUMC, 2018

Regimens	Specific regimens	Frequency (%)
Non-pharmacologic		6 (1.4)
Monotherapy		135(32.5)
	Enalapril	61 (14.7)
	Amlodipine	26 (6.2)
	Nifedipine	4 (1.0)
	Hydrochlorothiazide	40 (9.6)
	Losartan	4 (1.0)
Dual therapy		193 (46.4)
	ACEI + Diuretic	75 (18.0)
	CCB + BB	6 (1.4)
	Diuretic + BB	16 (3.8)
	ACEI + CCB	30 (7.2)
	ACEI + BB	24 (5.8)
	Diuretic +ARB	4 (1.0)
	CCB + ARB	8 (1.9)
	CCB+ Diuretics	26 (6.3)
	ARB+BB	4 (1.0)

Triple therapy	 BB + Diuretic + ACEI<br>CCB + BB + Diuretic<br>CCB +ACEI + Diuretic<br>CCB + BB + ACEI<br>CCB + BB +ARB<br>Others*	71 (17.1)<br>35 (8.4)<br>7 (1.7)<br>22 (5.3)<br>1 (0.2)<br>3 (0.7)<br>3 (0.7)
Quadruple	 ACEI + Diuretics + CCB + BB<br>ARB + BB + CCB + diuretic<br>Others**	11 (2.6)<br>7 (1.7)<br>1 (0.2)<br>3 (0.7)

others*: Enalapril +Furosemide +Spironolactone, Amlodipine +Furosemide + Hydrochlorothiazide; others**: Atenolol+ Enalapril+ Furosemide+ Spironolactone, Amlodipine+ Enalapril+ Furosemide+ Spironolactone

Clinicians' adherence to hypertension treatment guidelines

Twenty-five full-time physicians treat hypertensive patients during the study period. There were more males (21) than females (4). The mean age of prescribers was 29.22 ± 4.51 with range 24-39 years and the majority (17) of them were below 30 years of age. Six of them were general practitioners (GPs), 4 residency year 2 (R2), 8 of them are residency year 3(R3) and 7 of them were internist. Nearly half of clinicians had experience 1-5 years and most of them treating more than 20 hypertensive patients per day. Most prescribers perceived that a JNC-8 hypertension treatment guideline was evidence-based and helpful in the management of patients. However, clinicians' overall adherence to JNC-8 guidelines was low, with only 184 (44.2%) of prescribers following them. From a total of 232 patients not treated based on the JNC-8 guideline, 102(44.0%) patients were not received first-line drugs based on compelling indication and race. In 64(27.6%) patients, combination treatment was not adjusted based on their current BP level (Table 5).

Table 5: Common problems observed from prescribed antihypertensive medications to hypertensive patients at JUMC concerning the recommendation of JNC-8 guideline, N=232.

**Compliance issue**	**Frequency**	**Percent**
Patients did not receive first-line drugs based on compelling indication and race	102	44.0

recommendation		
Combination treatment was not adjusted based on their current blood pressure level	64	27.6
Patients received inappropriate dose of medications	50	21.6
Patients were not on first-line drugs and the right dose of medication	16	6.9

Patients' Adherence to Hypertension Treatment

A total of 410 study participants received one or more antihypertensive. From patients' response to the eight-item Morisky medication adherence Scale, Overall, the prevalence of antihypertensive medication adherence 191 (46.6%)

Determinants of medication non-adherent among hypertensive patients at JUMC, 2018

In bivariate logistic regression analysis; age, sex, marital status, residency, occupation, monthly income, living status, salt use, chat chewing, physical activity status, concomitant medication, comorbid condition, and duration of treatment were variables with P-value less than 0.25 hence, included in the multivariate logistic regressions. However, current occupation, physical activity status, and presence of DM comorbidity were significantly associated with medication non-adherence. Accordingly, merchants were 2.46 times (AOR=2.46, CI=1.16-5.23, P=0.020) more likely to be non-adherent than civil servants. Patients who had no regular physical activity were 1.63 times (AOR= 1.63, CI =1.04-2.55, P=0.033) more likely to be nonadherent compared to physically active patients. Hypertensive patients with DM comorbidity were 2.54 times (AOR= 2.54, CI=1.28-5.04, P=0.008) more likely to be non-adherent compared to clients with no comorbidity (Table 6).

Table 6: Results of logistic regression analysis for factors associated with medication nonadherence among adult hypertensive patients at JUMC, 2018

Variables		Medication adherence		COR (95%CI) N=410	P-value	AOR (95%CI)	P- value
		Adherent	Not Adherent				
**Occupation**	Civil servant	40	27	1.00	1.000	1.00	1.000
	Merchant	30	58	2.86(1.48-5.53)	0.002	2.46(1.16-5.23)	0.020
	Farmer	29	43	2.20(1.12-4.33)	0.023	1.41(0.52-3.78)	0.501
	House wife	24	22	1.36(0.64-2.89)	0.428	1.12(0.43-2.91)	0.812
	Retired	12	24	2.66(1.27-6.92)	0.012	2.04(0.66-6.26)	0.215
	Jobless	38	32	1.25(0.63-2.46)	0.522	0.71(0.26-1.91)	0.500
	Others	18	13	1.07(0.45-2.54)	0.878	0.73(0.26-2.08)	0.560
**Physical activity**	Inactive	89	130	1.67(1.13-2.48)	0.010	1.63(1.04-2.55)	0.033
	Active	102	89	1.00	1.000	1.00	1.000
**Comorbidities**	HTN alone	84	72	1.00	1.000	1.00	1.000
	DM	37	64	2.02(1.21-3.37)	0.007	2.54(1.28-5.04)	0.008
	CKD	11	5	0.53(0.18-1.59)	0.260	0.51(0.15-1.71)	0.272
	CHF	14	17	1.42(0.65-3.01)	0.378	1.14(0.48-2.71)	0.763
	CHD	13	25	2.24(1.10-4.70)	0.032	2.13(0.88-5.14)	0.093
	DM+CKD	11	5	0.53(0.18-1.60)	0.260	0.51(0.15-1.79)	0.294
	Dyslipidemia	17	21	1.44(0.71-2.94)	0.315	1.14(0.51-2.53)	0.751
	Others**	4	10	2.92(0.88-9.69)	0.081	2.81(0.77-10.22)	0.116

COR= crude odds ratio; AOR= Adjusted odds ratio: others**: Drivers, daily laborer, non-governmental organization and private work; others**: Anemia, peripheral neuropathy, lung disease, liver disease, thyroid disorder, and human immunodeficiency virus infection

Discussion

Medication adherence is the main predictor of treatment success and an effective step in controlling BP. The present study showed that the overall incidence of patients' medication adherence was 191 (46.6%). The finding was almost similar to the WHO report in 2011 in developing countries which was 50% [38]. It was, also closely similar to studies done in Palestinian (45.8%) and Saudi Arabia (46%) [39, 40]. However, it was lower than studies reported in Taiwan (53%), Sweden (63.1%), and China (53.4%) [41, 42, 43]. Variations in the studied populations, better health care and access to health facilities might be contributing to the adherence variation between the studies. Moreover, methods of measurement used to assess medication adherence also could be the reason for variation between the studies. For example, in Taiwan medication adherence was measured using the medication possession ratio (percentage of time that the patient had medication available to them during the follow-up period) whereas, in Sweden adherence was measured by using the Proportion of Days Covered (PDC) method.

The current finding also lower than the studies conducted in Gondar and Jimma university which reported 64.6% and 61.8%, respectively [44-45]. This variation might be due to inclusion criteria and adherence measurement scale. The study at Jimma university included patients that had at least one year follow up and the sample size (280 patients) was lower than this study. In Gondar university, adherence was measured using Morisky 4-item Medication Adherence Scale. On the other hand, the adherence level reported in this study was higher than the finding from Ghana and Nigeria (30.3%) and Iran (24%) [46-47]. This difference might be explained by studies done in Ghana and Nigeria were included hypertensive patients with depression in their study hence, psychiatric illnesses contribute to low medication adherence. Similarly, in Iran participants were selected from the rural area only and this might have contributed to medication nonadherence.

In this study, a significant association was observed between patients' current occupation and medication non-adherence. Merchant hypertensive patients were 2.46 times more likely to be non-adherent than civil servants. This finding is in line with studies done in Hong Kong China and Black Loin Specialized Hospital, Ethiopia [48-49]. This might be due to forgetfulness when they travel or leave home along with their medicine. Besides, they might be too busy to come to

the health facility for their pills and difficulty remembering to take all their medicine on time. Another important factor that influences patient compliance in this study was physical inactivity. Patients who had no regular physical activity were 1.63 times more likely to be non-adherent compared to patients who were physically active which, was consistent with finding from Iran [47]. The exact mechanism was unclear but might be that physically inactive hypertensive patients will have uncontrolled BP (57.2 % in this study) so that clinician may be prescribed a more complex treatment regimen. Also, uncontrolled blood pressure patients might be hopeless to take their medication and adhere to their treatment plan.

In addition, the presence of DM comorbidity was significantly associated with poor antihypertensive medication adherence. Hypertensive patients with DM comorbidity were 2.54 times more likely to be non-adherent compared to those with no co-morbidities. The finding was similar to a study conducted in South Korea, Hong Kong China, Saudi Arabia, and Gondar, Ethiopia [10,48, 40, 44]. Possibly this is explained by patients with co-morbidities who could suffer from serious complications and complex treatment regimens which were unfavorable conditions to adhere to their medications. Also, 27.4% of participants in this study had no formal education, might be difficult to adhere to treatment plan and taking on time with complex treatment

The proportion of clinicians' adherence to hypertension treatment guidelines found in this study was 44.2%. The result was slightly higher than the study conducted in Zewditu Memorial Hospital, Ethiopia (37.4%) [26] This difference might be due to the small sample size and most of the study participants (65.2%) were without co-morbid condition in Zewditu Memorial Hospital. However, it was lower than studies conducted in South Indian (65%), Malaysia (85.30%), Island (70.4%), and South Africa (51.9%) [21, 23, 37, 29]. This difference might be due to race difference, health care setting that is lack of laboratory and imaging facilities for further screening for target organ damage in our setting. In addition, it might be explained by the difference in doctors' profiles since the majority of doctors treating hypertensive patients in this study were general practitioners and residents. Six of them were general practitioners (GP), 4 residency year 2 (R2), 8 of them are residency year 3(R3 from the total of 25 doctors, respectively.

CCBs and thiazide diuretics are the recommended first-line medicines for hypertension of Africa origin as per JNC-8 guidelines. However, from this study clinicians prescribed ACEIs followed by diuretics and CCBs (63.7%, 57.1%, 35.1%), respectively. Among hypertensive patients with DM, CHF or CHD the most prescribed class of drugs was ACEI (66.3%, 71.0%, and 65.8% respectively), whereas in CKD most prescribed drug was diuretics. The overall use of ACEIs was similar to studies conducted in Serbia (60.57%) and Mexico City (63.8%) [30-31]. Studies performed in the Eastern Central Region of Portugal, and rural tertiary hospitals in Nigeria showed that thiazide diuretics were the most frequently prescribed class of medication (67%, and 84.9%), respectively [29, 16]. This difference might be due to studies in Portugal and Nigeria used JNC-7 as a reference guideline in which is conservative toward the thiazide-type diuretics as initial therapy for most patients without compelling indication.

The result of this study showed that 135 (32.5%) patients were on mono-therapy regardless of the presence or absence of comorbidities, which was lower than the studies reported from Mexico (72.1%), Canada (56.3%), and Turkey (75.7%) [31-33]. The difference might be due to better health care help to achieve target BP with a single medication in these countries. On the other hand, the majority of the patients, 275(66.1%) were on combination therapy. The finding was consistent with the study conducted in Kenya (60%) [34]. It was also similar to a study conducted at Zewditu Memorial Hospital Ethiopia (70.8%) [26]. However, the finding of this study was higher than the study conducted in Malaysia (56.7%) [35]. The higher prescription rate of combination therapy in this study might be due to the low rate of BP control 42.8% in this study as compared to 84.6% in Malaysia.

From the total of 135 (32.5%) patients who were on mono-therapy ACEI, 61 (14.7%) was the most prescribed drug class. The result was closely similar to a study conducted in Kenya (20.2%) [34]. However, it was lower than the study conducted in Turkey (30.1%) [33]. The variation might be due to using different standard reference guidelines in which European guidelines recommend any class of drug as initial therapy. On contrary, the study conducted in Gondar hospital showed that thiazide diuretics were the most commonly prescribed mono-therapy (60.24%) [36]. This discrepancy might be because of the difference in level guidelines adherence (66.8% prescription based on JNC-8 guidelines) that recommended the use of diuretics for both

mono and combination therapy. In addition, variation may be different in the presence of CKD co-morbidity, as only 3.8% of participants had CKD comorbidity in this study.

Two drugs regimen was prescribed in 46.4 % of the hypertensive patients. ACEI + diuretics, 75 (18.0%) was mostly used two-drug combination therapy. The finding was in line with a study conducted in Kenya (14.5%) [34]. A study conducted in India using the JNC-8 guideline showed that the most frequently prescribed two-drug combination was ARB +diuretics [25]. This variation might be due to the cost and easy accessibility of ARB in India. On the other hand, in the study conducted in Nigeria, CCB + diuretic was the most frequently used two-drug combinations (36.6%) [16]. this variation might be suggesting of doctor's preference to use CCBs combination as initial therapy. Besides, in Nigeria majority of the patients were elderly (mean age was 61.5±15.1 years) hence, CCBs preferable for older patients because of isolated SBP more prevalence due to vessel stiffness.

Three drug combination antihypertensive therapies accounted for 17.1% of all prescription, of which BB+ diuretics +ACEI was most frequent. The result was similar to studies conducted in Turkey, Kenya, and Gondar hospital, Ethiopia [34, 33, 36]. On the other hand, in India and Nigeria, the most frequently prescribed was CCBs+ ACEIs+ Diuretics [25, 16]. This difference might be due to variation in the prevalence of the types of co-morbidities. Four drugs regimen was prescribed in 11(2.6%) hypertensive patients, ACEI +diuretics +CCB+BB 7(1.7%) were most frequently prescribed. This was consistent with a study conducted at Zewditu Memorial Hospital, Ethiopia [26]. In contrast, study in Nigeria the most frequent quadruple-therapy diuretics +CCBs +ACEIs+ methyldopa [16]. This discrepancy might be because of the difference in inclusion criteria, in Nigeria patients with heart failure were excluded from the study; this might be the reason why the use of BBs was lower as compared to this study.

Limitations of the Study

There are some limitations to this study. The study was conducted in one facility, therefore; the findings may not be generalized to reflect the health care setting in Ethiopia. Only prescription and co-morbidity data were used to examine compliance to treatment guidelines, which may reliable. Finally, this study was unable to identify factors affecting clinicians' adherence to standard treatment guidelines but will be an interesting area for future research.

Conclusion

The rate of medication adherence among hypertensive patients was poor. Merchant, physical inactivity, and presence of DM co-morbidity were factors associated with poor medication adherence among hypertensive patients at Jimma University Medical center. Prescribing patterns of antihypertensive drugs were inconsistent with JNC- 8 standard hypertension treatment guideline. Angiotensin-converting enzyme inhibitors were the most frequently prescribed class of anti-hypertensive drugs in both monotherapy and combination therapy and the majority of participants were on combination therapy. Effective education for patients to improve medication adherence and regular training for prescribers on practical treatment guidelines is recommended to keep them up-to-date with current trends of hypertension treatment and for better treatment outcomes.

Abbreviation

BP: blood pressure, SBP: systolic blood pressure, DBP: Diastolic blood pressure, CHD: coronary heart disease, DM: diabetes mellitus, CKD: chronic kidney disease, CVD: cardiovascular diseases, IBM: international business machine, JNC-8: eight joint national committee, BB: beta-blocker, CCB: calcium channel blocker, ARB: angiotensin receptor blocker, ACEI: angiotensin-converting enzyme inhibitor, TD: thiazide diuretic, MMAS-8: Morisky medication adherence scale-8, AOR: adjusted odds ratio, COR: crude odds ratio.

Declarations

Acknowledgments: We thank the participants of the study.

Availability of data and materials: All relevant data are within the manuscript and and its supporting Information files.

Authors' contributions: Bekalu Kebede: concept and design, data acquisition, interpretation of data, manuscript preparation; Legese Chelkeba: concept and design, interpretation of data, manuscript preparation, manuscript revision, and manuscript review. Bekalu Dessie: interpretation of data, manuscript preparation, manuscript revision, and manuscript review. All authors read and approved the final manuscript

Consent for publication: Not applicable

Competing interests: The authors declare that they have no competing interest

References

1. Catherine P. Benziger GA. Roth AE. The Global Burden of Disease Study and the Preventable Burden of NCD. Journal of global heart.2016;11(4):393-397

2. Bromfield S, Muntner P. high blood pressure: the leading global burden of disease risk factor and the need for worldwide prevention programs. Curr Hypertens Rep. 2013;15(3):134-6.

3. Belue R, Okoror TA, Iwelunmor J, Taylor KD, Degboe AN, Agyemang C, et al. Globalization and Health An overview of cardiovascular risk factor burden in sub-Saharan African countries: a socio-cultural perspective. 2009; 12:1–12

4. Kibret KT, Mesfin YM. Prevalence of hypertension in Ethiopia: a systematic meta-analysis. Public Health Rev. 2015; 36:14.

5. Molla M. Systematic Reviews of Prevalence and Associated Factors of Hypertension in Ethiopia: Finding the Evidence. *Science Journal of Public Health*. 2015,3(4), 514-519.

6. Sarki AM, Nduka CU, Stranges S, Kandala NB, Uthman OA. Prevalence of Hypertension in Low- and Middle-Income Countries: A Systematic Review and Meta-Analysis. Medicine (Baltimore). 2015;94(50): e1959.

7. Ibrahim MM, Damasceno A. Hypertension in developing countries. Lancet. 2012;380(9841):611-9.

8. Grassi G, Cifkova R, Laurent S, Narkiewicz K, Redon J, Farsang C, et al. Blood pressure control and cardiovascular risk profile in hypertensive patients from central and eastern European countries. Eur Heart J. 2011; 32, 218–225

9. Schmieder RE, Mozaffarian D, Véronique L, Emelia J, Jarett D, Michael J et al. End Organ Damage in Hypertension. Circulation;2014; 129(3):1-19.

10. Shin S, Song H, Sang-Kwon Oh, Eob KC, Kim H, Jang S. Effect of antihypertensive medication adherence on hospitalization for cardiovascular disease and mortality in hypertensive patients. Hypertension Research.2013; 36(11):1000-1005.

11. Stevens PE, Levin A. Annals of Internal Medicine Clinical Guideline Evaluation and Management of Chronic Kidney Disease: Synopsis of Kidney Disease. 2013;825–31.

12. Ettehad D, Emdin CA, Kiran A, Anderson SG, Callender T, Emberson J, et al. Blood pressure lowering for prevention of cardiovascular disease and death: a systematic review and meta-analysis. Lancet, 2016; 387(10022), 957–967.

13. Jellinger PS, Handelsman Y, Rosenblit PD, Bloomgarden ZT, Fonseca VA, Garber AJ, Grunberger G, etal. AMERICAN ASSOCIATION OF CLINICAL ENDOCRINOLOGISTS AND AMERICAN COLLEGE OF ENDOCRINOLOGY GUIDELINES FOR MANAGEMENT OF DYSLIPIDEMIA AND PREVENTION OF CARDIOVASCULAR DISEASE. Endocr Pract. 2017 Apr;23(Suppl 2):1-87

14. James PA, Oparil S, Carter BL, Cushman WC, Dennison-Himmelfarb C, Handler J etal. 2014 Evidence-Based Guideline for the Management of high blood pressure in adults report from the panel members appointed to the eighth Joint National Committee (JNC 8). JAMA. 2014;311(5):507-20

15. Gebrihet TA, Mesgna KH, Gebregiorgis YS, Kahsay AB, Weldehaweria NB, Weldu G. Awareness, treatment, and control of hypertension is low among adults in Aksum town, northern Ethiopia: A sequential quantitative-qualitative study. PLoS One. 2017;12(5):e0176904.

16. Busari OA, Oluyombo R, Fasae AJ, Gabriel EO, Ayodele LM, Agboola SM. Prescribing pattern and utilization of antihypertensive drugs and blood pressure control in adult patients with systemic hypertension in a rural tertiary hospital in Nigeria. Am J Inter Med. 2014;2(6):144-9

17. Parati G, Omboni S, Compare A, Grossi E, Callus E, Venco A, et al. Blood pressure control and treatment adherence in hypertensive patients with metabolic syndrome: protocol of a randomized controlled study based on home blood pressure telemonitoring vs. conventional management and assessment of psychological determinants of adherence(TELEBPMET Study). trials; 2013;1–11.

18. Asgedom SW, Gudina EK, Desse TA. Assessment of Blood Pressure Control among Hypertensive Patients in Southwest Ethiopia. PLoS One. 2016;11(11):e0166432.

19. Papatya Karakurt, Mağfiret Kaşikçi, Factors affecting medication adherence in patients with hypertension, Journal of Vascular Nursing. 201230(4), 118-126,

20. Rolnick SJ, Pawloski PA, Hedblom BD, Asche SE, Bruzek RJ. Patient characteristics associated with medication adherence. Clin Med Res. 2013;11(2):54-65.

21. Datta S. Utilization Study of Antihypertensives in a South Indian Tertiary Care Teaching Hospital and Adherence to Standard Treatment Guidelines. J Basic Clin Pharm. 2016;8(1):33-37.

22. Mutua EM, Gitonga MM, Mbuthia B, Muiruri N, Cheptum JJ, Maingi T. Level of blood pressure control among hypertensive patients on follow-up in a regional referral hospital in Central Kenya. Pan Afr Med J. 2014;18:278.

23. Abdulameer SA, Sahib MN, Aziz NA, Hassan Y, Alrazzaq HA, Ismail O. Physician adherence to hypertension treatment guidelines and drug acquisition costs of antihypertensive drugs at the cardiac clinic: a pilot study. Patient Prefer Adherence. 2012;6:101-8.

24. Gudina EK, Michael Y, Assegid S. Prevalence of hypertension and its risk factors in southwest Ethiopia: a hospital-based cross-sectional survey. Integr Blood Press Control. 2013;6:111-7.

25. Romday R, Gupta A, Bhambani P. An assessment of antihypertensive drug prescription patterns and adherence to joint national committee-8 hypertension treatment guidelines among hypertensive patients attending a tertiary care teaching hospital. International Journal of Research in Medical Sciences. 2016;4(12).5125-33.

26. Yazie D, Shibeshi W, Woldu M, Berha A. Assessment of Blood Pressure Control among Hypertensive Patients in Zewditu Memorial Hospital, Addis Ababa, Ethiopia: A Cross-Sectional Study. Journal of Bioanalysis & Biomedicine. 2018;10.1-53

27. Pittrow D, Kirch W, Bramlage P, Lehnert H, Höfler M, Unger T et al. Patterns of antihypertensive drug utilization in primary care. Eur J Clin Pharmacol. 2004; 60(2):135-42.

28. Morgado MP, Rolo SA, Pereira L, Castelo-Branco M. Blood pressure control and antihypertensive pharmacotherapy patterns in a hypertensive population of Eastern Central Region of Portugal. BMC Health Serv Res. 2010;10:349.

29. Adedeji AR, Tumbo J, Govender I. Adherence of doctors to a clinical guideline for hypertension in Bojanala district, North-West Province, South Africa. Afr J Prim Health Care Fam Med. 2015;7(1):776.

30. Tomas A, Tomić Z, Milijasević B, Ban M, Horvat O, Vukmirović S et al. Patterns of prescription antihypertensive drug utilization and adherence to treatment guidelines in the city of Novi Sad. Vojnosanit Pregl. 2016;73(6):531-7.

31. Alba-Leonel A, Carvajal A, Fierro I, Castillo-Nájera F, Campos-Ramos O, Villa-Romero A et al. Prescription patterns of antihypertensives in a community health centre in Mexico City: a drug utilization study. Fundam Clin Pharmacol. 2016; 30(3):276-81.

32. Beaulieu MD, Dufresne L, LeBlanc D. Treating hypertension. Are the right drugs given to the right patients? Can Fam Physician. 1998; 44:294-8, 301-2.

33. Abaci A, Kozan O, Oguz A, Sahin M, Deger N, Senocak H et al. Prescribing pattern of antihypertensive drugs in primary care units in Turkey: results from the TURKSAHA study. Eur J Clin Pharmacol. 2007 Apr;63(4):397-402.

34. Mbui JM, Oluka MN, Guantai EM, Sinei KA, Achieng L, Baker A et al. Prescription patterns and adequacy of blood pressure control among adult hypertensive patients in Kenya; findings and implications. Expert Rev Clin Pharmacol. 2017; 10(11):1263-1271.

35. Ramli A, Miskan M, Ng K, Ambigga D, Nafiza M, Mazapuspavina M, Sajari J, Ishak R. Prescribing of Antihypertensive Agents in Public Primary Care Clinics - is it in Accordance with Current Evidence? Malays Fam Physician. 2010;5(1):36-40.

36. Abegaz TM, Tefera YG, Abebe TB. Antihypertensive drug prescription patterns and their impact on outcome of blood pressure in Ethiopia: a hospital-based cross-sectional study. Integr Pharm Res Pract. 2017; 6:29-35.

37. Theodorou M, Stafylas P, Kourlaba G, Kaitelidou D, Maniadakis N, Papademetriou V. Physicians' perceptions and adherence to guidelines for the management of hypertension: a national, multicentre, prospective study. Int J Hypertens. 2012;2012:503821.

38. Brown MT, Bussell JK. Medication adherence: WHO cares? Mayo Clin Proc. 2011; 86(4):304-14.

39. Al-Ramahi R. Adherence to medications and associated factors: A cross-sectional study among Palestinian hypertensive patients. J Epidemiol Glob Health. 2015; 5(2):125-32.

40. Khayyat SM, Khayyat SM, Hyat Alhazmi RS, Mohamed MM, Abdul Hadi M. Predictors of Medication Adherence and Blood Pressure Control among Saudi Hypertensive Patients Attending Primary Care Clinics: A Cross-Sectional Study. PLoS One. 2017;12(1):e0171255.

41. Lee CY, Huang CC, Shih HC, Huang KH. Factors influencing antihypertensive medication compliance in Taiwan: a nationwide population-based study. Eur J Prev Cardiol. 2013; 20(6):930-7.

42. Hedna K, Hakkarainen KM, Gyllensten H, Jönsson AK, Andersson Sundell K, Petzold M, Hägg S. Adherence to Antihypertensive Therapy and Elevated Blood Pressure: Should We Consider the Use of Multiple Medications? PLoS One. 2015;10(9):e0137451.

43. Li YT, Wang HHX, Liu KQL, Lee GKY, Chan WM, Griffiths SM et al. Medication Adherence and Blood Pressure Control Among Hypertensive Patients With Coexisting Long-Term Conditions in Primary Care Settings: A Cross-Sectional Analysis. Medicine (Baltimore). 2016; 95(20):e3572.

44. Ambaw AD, Alemie GA, W/Yohannes SM, Mengesha ZB. Adherence to antihypertensive treatment and associated factors among patients on follow up at University of Gondar Hospital, Northwest Ethiopia. BMC Public Health. 2012;12:282.

45. Asgedom SW, Atey TM, Desse TA. Antihypertensive medication adherence and associated factors among adult hypertensive patients at Jimma University Specialized Hospital, southwest Ethiopia. BMC Research Notes. 2018;11(1):27.

46. Boima V, Ademola AD, Odusola AO, Agyekum F, Nwafor CE, Cole H, et al. Factors Associated with Medication Nonadherence among Hypertensives in Ghana and Nigeria. Int J Hypertens. 2015;2015:205716.

47. Kamran A, Sadeghieh Ahari S, Biria M, Malepour A, Heydari H. Determinants of Patient's Adherence to Hypertension Medications: Application of Health Belief Model Among Rural Patients. Ann Med Health Sci Res. 2014; 4(6):922-7.

48. Kang CD, Tsang PP, Li WT, Wang HH, Liu KQ, Griffiths SM et al. Determinants of medication adherence and blood pressure control among hypertensive patients in Hong Kong: a cross-sectional study. Int J Cardiol. 2015 Mar 1;182:250-7.

49. Hareri HA, Abebe M and Asefaw T: Assessments of adherence to Hypertension managements and its influencing factors among Hypertensive patients attending Black Lion Hospital chronic follow up unit, Addis Ababa, Ethiopia-a cross-sectional study. *Int J Pharm Sci Res* 2013; 4(3); 1086-1095.

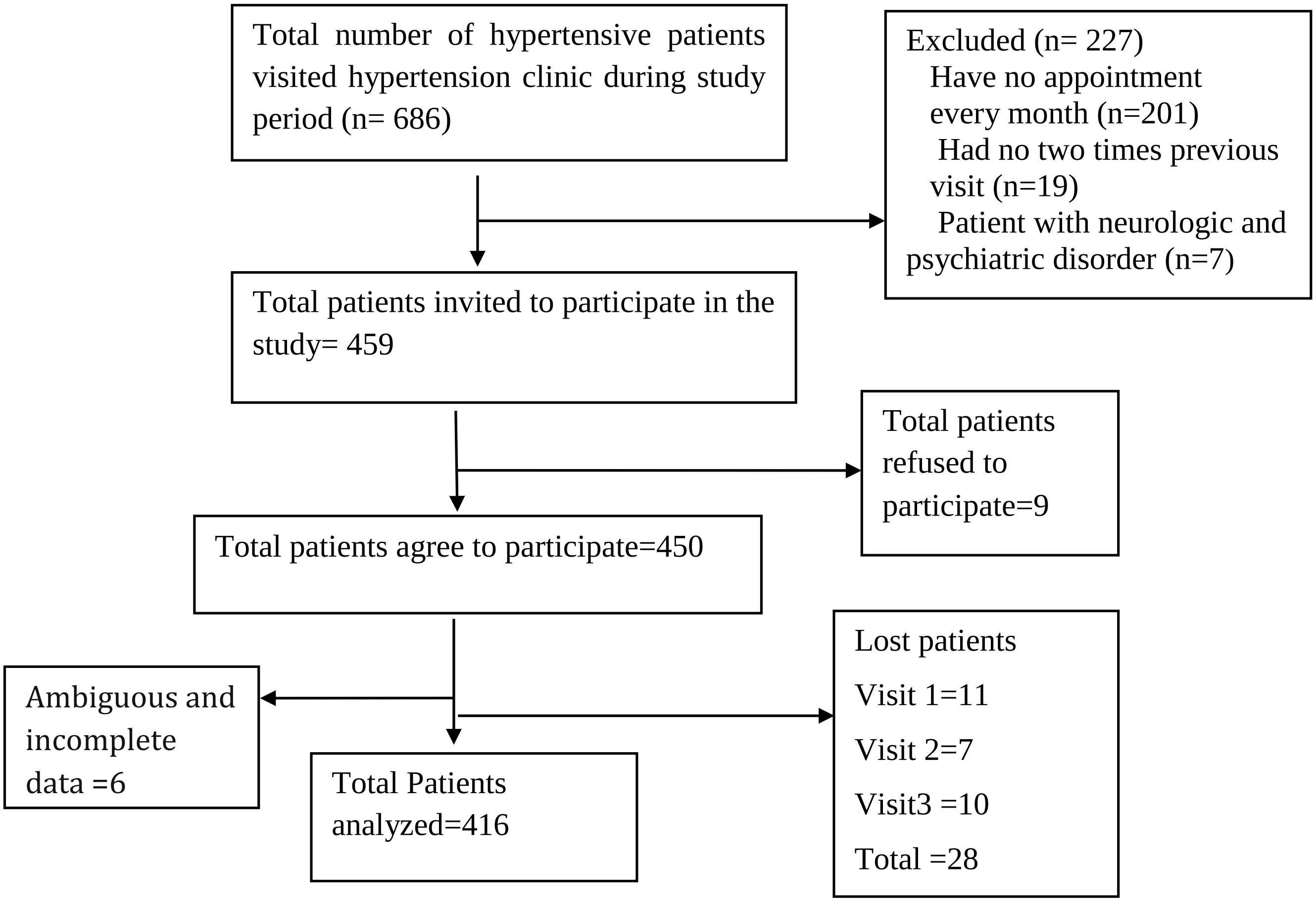
Total number of hypertensive patients visited hypertension clinic during study period (n= 686)
Excluded (n= 227)
Have no appointment every month (n=201)
Had no two times previous visit (n=19)
Patient with neurologic and psychiatric disorder (n=7)
Total patients invited to participate in the study= 459
Total patients refused to participate=9
Total patients agree to participate=450
Ambiguous and incomplete data =6
Total Patients analyzed=416
Lost patients
Visit 1=11
Visit 2=7
Visit3 =10
Total =28

Click here to access/download
Supporting Information
Questionnaire.docx

Clinicians' Prescribing Pattern, Rate of Patients' Medication Adherence and Its Determinants among Adult Hypertensive Patients at Jimma University Medical Center: Prospective Cohort Study

Bekalu Kebede,MSc[1], Legese Chelkeba, PhD[2], Bekalu Dessie,MSc[1]

[1] Department of Pharmacy, College of Health Sciences, Debre Markos University, Debre Markos, Ethiopia

[2]Department of Clinical Pharmacy, School of Pharmacy, College Health Sciences, Addis Ababa University, Jimma, Ethiopia

Corresponding author: Bekalu Kebede

Clinical Pharmacist, College of Health, Department of pharmacy, Debre Markos University, Debre Markos, Ethiopia

Phone: +251-921-286-140

Email:bekalukebede19@gmail.com

Abstract

Background: Despite the goals of hypertension treatment are well defined and effective therapies are widely available, control of hypertension remains poor, and hypertension-related cardiovascular diseases are still high in most health care settings. Therefore, this study aimed to determine clinicians' prescribing patterns, patients' medication adherence, and its determinants among ambulatory hypertensive patients at Jimma University Medical Center.

Methods: A general prospective cohort study was conducted among hypertensive patients who had regular follow-up at Jimma university ambulatory cardiac clinic from March 20, 2018, to June 20, 2018. Patients' specific data was collected with a face-to-face interview and from their medical chart. Clinicians' related data were collected through a self-administered questionnaire. Data were analyzed using SPSS version 21.0. Bivariate and multivariable logistic regression analyses were done to identify key independent variables influencing patients' adherence. P-Values of less than 0.05 were considered statically significant.

Results: From the total of 416 patients, 237(57.0%) were males with a mean age of 56.50 ± 11.96 years. Angiotensin-converting enzyme inhibitors were the most frequently prescribed class of antihypertensives accounting for 261(63.7%) prescriptions. The majority of patients, 275 (66.1%) were on combination therapy. The rate of patients' medication adherence and clinicians' guidelines adherence was 46.6%, and 44.2%, respectively. Patients with merchant occupation (P=0.020), physical inactivity (P=0.033), and diabetes mellitus co-morbidity (P=0.008) were significantly associated with medication non-adherence.

Conclusion: The rate of medication adherence among hypertensive patients was poor and physicians were not adhering to standard treatment guidelines. Angiotensin-converting enzyme inhibitors were the most frequently prescribed class of drugs. Effective education for patients to improve medication adherence and regular training for prescribers on practical treatment guidelines is recommended to keep them up-to-date with current trends of hypertension treatment and for better treatment outcomes.

Keywords: Prescribing Pattern, Blood Pressure Control, Guidelines, Adherence

Introduction

Hypertension a significant public health challenge worldwide due to its high prevalence and concomitant risks of cardiovascular and kidney diseases [1]. The World Health Organization (WHO) has estimated that high blood pressure (BP) is a major public health issue and causes one in every eight deaths, and is the third leading silent killer in the world [2].

Almost three-quarters of people with hypertension (639 million people) live in countries with limited health resources [1, 2]. In Sub- Saharan Africa, it is a major independent risk factor for heart failure, stroke, and kidney failure. These complications result due to a low rate of hypertension diagnosis, suboptimal BP control, high morbidity and mortality, and low resources in health care settings [3]. A systematic review and meta-analysis study done in Ethiopia in 2015 showed that the prevalence of hypertension was estimated to be 19.6 % [4]. Another systemic review and meta-analysis conducted in Ethiopia in 2015 reported that the prevalence of hypertension was between 20% and 30% [5].

Although hypertension is a preventable and modifiable risk factor for cardiovascular diseases (CVD), the prevention and control of hypertension have not yet received attention in many developing countries [6,7]. It accounts for 40.6% of deaths due to all coronary heart disease and 38.5% of deaths due to stroke [8]. It is also the second-leading cause of end-stage renal disease (ESRD) behind diabetes mellitus (DM [9].

Guideline adherence is defined as a condition in which the prescribed treatment obeys treatments recommended in the identified practice guidelines [10-12]. Hypertension guidelines are evidence-based and are usually dictated by randomized controlled trial data and observational studies. Published guidelines aid in clinical decision making, decrease practice variations, guide correctness, and measure the quality of health care [13-14]. The Eighth Joint National Committee (JNC-8) on detection, evaluation, and treatment of BP is most the commonly used guideline and it is considered as a "gold standard" consensus guideline for the management of hypertension [15]. This guideline was used as a reference standard for this study because awareness and accessibility of guidelines were initial criteria to evaluate the status of clinicians' adherence to hypertension treatment guidelines. Besides, Ethiopia has not its hypertension treatment guideline.

Therefore, JNC-8 guideline is widely used by both clinicians and clinical pharmacists in Ethiopia [16]. However, clinical practice guidelines do not consistently change clinicians' behavior, control of high blood pressure remains suboptimal, so creating the need to evaluate adherence to guidelines and its impact on BP control [10].

Many studies conducted in the past to explore the causes of suboptimal BP control focused on patient factors such as socio-demographic, medical profile, and patient's treatment compliance [17-20]. However, prescribing patterns and clinicians' adherence to guidelines are also important factors affecting hypertension treatment outcomes [21-24].

Several studies were conducted worldwide using either prescription or drug dispensing data to evaluate prescription patterns and clinicians 'adherence to hypertension treatment guidelines [25-27]. But few data are available in Ethiopia. Also, among previous studies, there were conflicting results between prescribing pattern and patient's medication adherence. For example, a study conducted in Kenya concludes that combination therapies were associated with poor patient adherence [22]. In contrast, according to a study conducted in Nigeria combination therapies were associated with good blood pressure controlled than mono-therapy [16]. A previous study conducted at Jimma University, Ethiopia showed that the number of antihypertensive medications prescribed was not associated with blood pressure control status [18]. Besides, most previous studies used a similar study design(cross-sectional). As a result, a general prospective cohort study was conducted among ambulatory hypertension patients to determine prescribing patterns of antihypertensive drugs, patient's medication adherence, and its determinants.

Methods

Study period and setting

This study was conducted over a three-month period among hypertensive patients who had regular followed up every month at Jimma university ambulatory cardiac clinic from March 20, 2018, to June 20, 2018. Jimma University is the only teaching and specialized hospital in Jimma zone. It provides service for over 15 million peoples living in Jimma city and its surroundings It is located in Jimma town, Oromia Regional State, Ethiopia, 352 km southwest of Addis Ababa, the capital city of Ethiopia (18).

Study design: a general prospective cohort follow-up study was employed

Study population; All hypertensive patients with age ≥ 18 years, with a documented diagnosis, had at least two times medical appointments before the study period, having a medical appointment every month and complete medical records were included in the study. We excluded pregnant women, patients with confirmed neurologic and psychiatric disorders with their respective specialists since it was challenging to assess medication adherence statuses in acutely ill patients with similar tools like that of other study participants.

Sample size and sampling technique: The sample size for this study was calculated using Cochran single proportion formula. A previous study conducted in Ethiopia reported that the rate of patient medication adherence was 50 % [18]. Based on this formula minimum 384 sample size was calculated using a standard normal distribution (Z=1.96) with a confidence interval of 95% and a margin of error of 0.05. After adjusting 10% non-respondent rate, 416 samples were used in this study. All clinicians who treated hypertensive patients during the study period were included and a random sampling technique was used for patients.

Data collection and tool: questionnaire and data abstraction format were prepared by reviewing different kinds of literature. To maintain the validity of the data collection tool, a structured questionnaire was developed translated to local languages (Amharic and Afan Oromo) by native speakers of the respective languages and then back translated to the English version. Pre-test of data collection format was performed on 5% of the sample. Primary data from patients were collected by face-to-face interview and data from all clinicians who treat hypertensive patients during the study period was collected by using a self-administer questionnaire. Secondary data such as prescribed drugs, blood pressure measurements, and co-morbid illness was collected from patients' chart daily during their visit. At Jimma University Medical Center, initial and subsequent BP typically measured using the cuff sphygmomanometer and digital sphygmomanometer on the left arm, at the level of the heart, in a sitting position, and at rest for at least 5 minutes. In this study hypertensive patients that fulfilled the inclusion criteria were selected at the first month of the data collection period and BP measurement at this first visit was considered as a baseline. Then only those patients selected at the first month were consequently followed every month for the next three months.

Morisky Medication Adherence Scale-8 (MMAS-8) was used to assess the patients' medication adherence status. The MMAS-8 total score was calculated by summing the

values from all 8 items, with reverse coding when necessary. A score of <2 (out of a full range of 8) was considered as good medication adherence, otherwise considered as poor adherence [19].

Pharmacological Trends Guideline Adherence Evaluation Method was used to measure clinicians' adherence with hypertension treatment guidelines [25]. A recommendation of the JNC-8 on the detection, evaluation, and treatment of high BP was used as a reference guideline to evaluate prescription patterns and clinicians' adherence to the guidelines.

Data processing and analysis: The data were entered using Epidata version 3.1 and exported to the Statistical Package for Social Science (SPSS) version 21.0. Continuous variables were presented as means (standard deviation). Categorical variables were presented as frequency and percentages. Antihypertensive medicines were categorized according to their therapeutic classes. Switch from one drug to another drug during each visit, the last regimen was included in the analysis unless changing was done at the last visit.

Clinicians' guideline adherence was measured by the proportion of the total number of cases (hypertensive patients) treat based on the JNC-8 guideline with the total number of participants. Guideline adherence was expressed as % = (Total number hypertensive patients treat based on JNC-8 guideline divide by the total number of patients) × 100 [23]. Based on this calculation >65% conceder as complete adherence, 50 – 64.9% medium adherence, and <49.9% will be classified as low adherence [29]. Finally, BP was calculated by taking an average of three measurements and categorized controlled and uncontrolled based on guidelines.

Before regression was done a multi-collinearity test, and adequacy of cell distribution was checked using chi-square test. Binary logistic regression was performed to determine the effect of each variable on patients' adherence and variables with a P-value less than 0.25 in the bivariate analysis were then included in a multivariate logistic regression analysis to identify key independent variables influencing patient's medication adherence. In multivariate analysis, variables with P- a value of < 0.05 were considered statistically significant.

Ethics approval and consent to participate: This study was approved by the Ethical Review Board of School of Pharmacy, Institute of Health Science Jimma University (Ref.No IHRPGD/203/18). First, each participant was asked orally whether they were voluntary or not to participate in this study after a detailed explanation of the objective of the study, procedures of selection, and assurance of confidentiality. An independent data collection supervisor acted as

witness for voluntary informed decision making of participants to take part in the study. Written informed consent was waived since the study did not involve any procedure and present no damage to patients as approved by the ethical review board committee of the Board of School of Pharmacy, Institute of Health Science Jimma University. Their names were not registered to minimize social desirability bias and enhance anonymity. They were not forced to participate or receive any monetary incentive and it was solely voluntary

Results

Baseline characteristics of study participants

A total of 686 hypertensive patients visited the hypertension clinic during the study period. Four hundred fifty-nine patients fulfilled inclusion criteria, of these, 416 participants were included in the final analyses (Fig 1). More than half (57.0%) of the participants were males with mean age of 56.50 ± 11.96 years (Table 1).

Table 1: Socio-demographic characteristics of hypertensive patients at JUMC, 2018

**Variables**	**Characteristics**	**Frequency (%)**
**Age (year)**	Mean ± SD	56.50±11.96
	<60	220(52.9)
	≥60	196(47.1)
**Sex**	Male	237 (57.0)
	Female	179 (43.0)
**BMI**	Mean ± SD	24.24±2.32
	18.5-24.9	314 (75.5)
	25-29.9	89 (21.4)
	≥30	13 (3.1)
**Marital status**	Single	12 (2.9)
	Married	284 (68.3)
	Divorced	55 (13.2)

	Widowed	65 (15.6)
**Residency**	Urban	274 (65.9)
	Rural	142 (34.1)
**Education level**	No formal education	114 (27.4)
	Primary education (1-8 grade)	110 (26.4)
	Secondary education (9-12 grade)	100 (24.0)
	Tertiary education (diploma & above)	92 (22.1)
**Current occupation**	Civil servant	69 (16.6)
	Merchant	89 (21.4)
	Farmer	74 (17.8)
	Housewife	47 (11.3)
	Retired	36 (8.7)
	Jobless	70 (16.8)
	Other	31 (7.5)
**Monthly income (ETB)**	<1000	58 (13.9)
	1001-2000	68 (16.3)
	2001-3000	74 (17.8)
	>3000	109 (26.2)
	Without unknown monthly income	107 (25.7)
**Living status**	I live alone	59 (14.2)
	I live with family	328 (78.8)
	Others*	29 (7.0)

Others: Drivers, daily laborer, non-governmental organization and private work; others*: live with friends, prison; ETB: Ethiopian Birr

Two hundred sixty-four (63.5%) of participants have been taking salt with food. More than half (53.4%) of the participants were physically inactive and 138 (33.2%) patients were khat chewers. Among co-morbid conditions, 104(25.0 %) of the participants had diabetes mellitus (DM), 38(9.1%) and 39(9.4%) had coronary heart disease (CHD) and dyslipidemia, respectively (Table 2).

Table 2: Lifestyle factors and clinical characteristics of hypertensive patients at JUMC, 2018

Factors	Categories	Frequency (%)
**Add salt to food**	Yes	264 (63.5)
	No	152 (36.5)
**Alcohol use**	Yes	103 (24.8)
	No	313 (75.2)
**Chew khat**	Yes	138 (33.2)
	No	278 (66.8)
**Cigarette smoking**	Never smoked	303 (72.8)
	Ex-smoker	93 (22.4)
	Current smoker	20 (4.8)
**Physical activity**	Physically active	194 (46.6)
	Physically inactive	222 (53.4)
**Drink coffee**	Yes	238 (57.2)
	No	178 (42.8)
**Use traditional medicine**	Yes	16 (3.8)
	No	400 (96.2)
**Co morbidity**	Hypertension alone	158 (38.0)
	DM	104 (25.0)
	CKD	16 (3.8)
	CHF	31 (7.5)
	CHD	38 (9.1)
	DM and CKD	16 (3.8)
	Dyslipidemia	39 (9.4)
	others	14 (3.4)
**Duration on treatment(years)**	<1	67 (16.1)
	1-5	148 (35.6)

	>5-10	144 (34.6)
	>10	57 (13.7)

Others:- Anemia, peripheral neuropathy, lung disease, liver disease, thyroid disorder, and human immunodeficiency virus infection

Prescription patterns of antihypertensive medications

The overall pattern of antihypertensive agents showed that angiotensin-converting enzyme inhibitor (ACEI) was the most frequently prescribed class of antihypertensive drugs 261(63.7%) followed by diuretics 234 (57.1%). Among the study participants, 257 (61.7%) of them had one or more concomitant medications, of which 98 (23.6%) and 75(18.0%) of them had two and three drugs concomitant medications, respectively (table 3)

Table 3: Frequency of antihypertensive medicines prescribed for hypertensive patients at JUMC, 2018.

Classes	Specific drugs	Frequency (%), N=410
**ACEIs**	Enalapril	261 (63.7)
**ARBS**	Losartan	28 (6.8)
**Diuretics**		234 (57.1)
	Hydrochlorothiazide	203 (49.5)
	Furosemide	26 (6.4)
	Furosemide +spironolactone	5 (1.2)
**CCBs**		144 (35.1)
	Amlodipine	131 (31.9)
	Nifedipine	13 (3.2)
**BBs**		105 (25.6)
	Atenolol	45 (10.9)
	Metoprolol	47 (11.5)
	Propranolol	13 (3.2)
**Co-medications**	Total number of patients with co-medication	257 (61.7)

	One drug	61 (14.7)
	Two drugs	98 (23.6)
	Three drugs	75 (18.0)
	≥ Four drugs	23 (5.5)

ACEIs: Angiotensin-converting enzyme inhibitors; ARBs: angiotensin receptor blockers; BBs: βblockers; CCBs: calcium channel blocker

From the total participants, 135 (32.5%) of them were on mono-therapy and 275 (66.1%) were on combination therapy. Enalapril was the most frequently prescribed monotherapy 61(14.7%) followed by hydrochlorothiazide 40(9.6%). Among patients who were on combination therapy, two-drug regimens were prescribed in 46.4% of the hypertensive patients. ACEI +diuretics, 75 (18.0%) was commonly used two-drug combination therapy. Three drugs combination accounted for 17(17.1%) of all prescriptions, of which 35 (8.4%) were on BB+ diuretics+ ACEI followed by CCB+ACEI +diuretic 22(5.3%). Four drugs regimen was prescribed in 11 hypertensive patients, ACEI +diuretics +CCB+BB 7(1.7%) was most frequent prescribed (Table 4).

Table 4: Regimens of antihypertensive therapy among hypertensive patients at JUMC, 2018

**Regimens**	**Specific regimens**	**Frequency (%)**
**Non-pharmacologic**		6 (1.4)
**Monotherapy**		135(32.5)
	Enalapril	61 (14.7)
	Amlodipine	26 (6.2)
	Nifedipine	4 (1.0)
	Hydrochlorothiazide	40 (9.6)
	Losartan	4 (1.0)
**Dual therapy**		193 (46.4)
	ACEI + Diuretic	75 (18.0)
	CCB + BB	6 (1.4)
	Diuretic + BB	16 (3.8)
	ACEI + CCB	30 (7.2)
	ACEI + BB	24 (5.8)
	Diuretic +ARB	4 (1.0)

	CCB + ARB<br>CCB+ Diuretics<br>ARB+BB	8 (1.9)<br>26 (6.3)<br>4 (1.0)
**Triple therapy**	 BB + Diuretic + ACEI<br>CCB + BB + Diuretic<br>CCB +ACEI + Diuretic<br>CCB + BB + ACEI<br>CCB + BB +ARB<br>Others*	71 (17.1)<br>35 (8.4)<br>7 (1.7)<br>22 (5.3)<br>1 (0.2)<br>3 (0.7)<br>3 (0.7)
**Quadruple**	 ACEI + Diuretics + CCB + BB<br>ARB + BB + CCB + diuretic<br>Others**	11 (2.6)<br>7 (1.7)<br>1 (0.2)<br>3 (0.7)

others*: Enalapril +Furosemide +Spironolactone, Amlodipine +Furosemide + Hydrochlorothiazide; others**: Atenolol+ Enalapril+ Furosemide+ Spironolactone, Amlodipine+ Enalapril+ Furosemide+ Spironolactone

Clinicians' adherence to hypertension treatment guidelines

Twenty-five full-time physicians treat hypertensive patients during the study period. There were more males (21) than females (4). The mean age of prescribers was 29.22 ± 4.51 with range 24-39 years and the majority (17) of them were below 30 years of age. Six of them were general practitioners (GPs), 4 residency year 2 (R2), 8 of them are residency year 3(R3) and 7 of them were internist. Nearly half of clinicians had experience 1-5 years and most of them treating more than 20 hypertensive patients per day. Most prescribers perceived that a JNC-8 hypertension treatment guideline was evidence-based and helpful in the management of patients. However, clinicians' overall adherence to JNC-8 guidelines was low, with only 184 (44.2%) of prescribers following them. From a total of 232 patients not treated based on the JNC-8 guideline,

102(44.0%) patients were not received first-line drugs based on compelling indication and race. In 64(27.6%) patients, combination treatment was not adjusted based on their current BP level (Table 5).

Table 5: Common problems observed from prescribed antihypertensive medications to hypertensive patients at JUMC concerning the recommendation of JNC-8 guideline, N=232.

**Compliance issue**	**Frequency**	**Percent**
Patients did not receive first-line drugs based on compelling indication and race recommendation	102	44.0
Combination treatment was not adjusted based on their current blood pressure level	64	27.6
Patients received inappropriate dose of medications	50	21.6
Patients were not on first-line drugs and the right dose of medication	16	6.9

Patients' Adherence to Hypertension Treatment

A total of 410 study participants received one or more antihypertensive. From patients' response to the eight-item Morisky medication adherence Scale, Overall, the prevalence of antihypertensive medication adherence 191 (46.6%)

Determinants of medication non-adherent among hypertensive patients at JUMC, 2018

In bivariate logistic regression analysis; age, sex, marital status, residency, occupation, monthly income, living status, salt use, chat chewing, physical activity status, concomitant medication, comorbid condition, and duration of treatment were variables with P-value less than 0.25 hence, included in the multivariate logistic regressions. However, current occupation, physical activity status, and presence of DM comorbidity were significantly associated with medication non-adherence. Accordingly, merchants were 2.46 times (AOR=2.46, CI=1.16-5.23, P=0.020) more likely to be non-adherent than civil servants. Patients who had no regular physical activity were 1.63 times (AOR= 1.63, CI =1.04-2.55, P=0.033) more likely to be nonadherent compared to physically active patients. Hypertensive patients with DM comorbidity were 2.54 times (AOR=

2.54, CI=1.28-5.04, P=0.008) more likely to be non-adherent compared to clients with no comorbidity (Table 6).

Table 6: Results of logistic regression analysis for factors associated with medication nonadherence among adult hypertensive patients at JUMC, 2018

**Variables**		**Medication adherence**		**COR (95%CI) N=410**	**P-value**	**AOR (95%CI)**	**P- value**
		Adherent	Not Adherent				
**Occupation**	Civil servant	40	27	1.00	1.000	1.00	1.000
	Merchant	30	58	2.86(1.48-5.53)	0.002	2.46(1.16-5.23)	0.020
	Farmer	29	43	2.20(1.12-4.33)	0.023	1.41(0.52-3.78)	0.501
	House wife	24	22	1.36(0.64-2.89)	0.428	1.12(0.43-2.91)	0.812
	Retired	12	24	2.66(1.27-6.92)	0.012	2.04(0.66-6.26)	0.215
	Jobless	38	32	1.25(0.63-2.46)	0.522	0.71(0.26-1.91)	0.500
	Others	18	13	1.07(0.45-2.54)	0.878	0.73(0.26-2.08)	0.560
**Physical activity**	Inactive	89	130	1.67(1.13-2.48)	0.010	1.63(1.04-2.55)	0.033
	Active	102	89	1.00	1.000	1.00	1.000
**Comorbidities**	HTN alone	84	72	1.00	1.000	1.00	1.000
	DM	37	64	2.02(1.21-3.37)	0.007	2.54(1.28-5.04)	0.008
	CKD	11	5	0.53(0.18-1.59)	0.260	0.51(0.15-1.71)	0.272
	CHF	14	17	1.42(0.65-3.01)	0.378	1.14(0.48-2.71)	0.763
	CHD	13	25	2.24(1.10-4.70)	0.032	2.13(0.88-5.14)	0.093
	DM+CKD	11	5	0.53(0.18-1.60)	0.260	0.51(0.15-1.79)	0.294
	Dyslipidemia	17	21	1.44(0.71-2.94)	0.315	1.14(0.51-2.53)	0.751
	Others**	4	10	2.92(0.88-9.69)	0.081	2.81(0.77-10.22)	0.116

COR= crude odds ratio; AOR= Adjusted odds ratio: others**: Drivers, daily laborer, non-governmental organization and private work; others**: Anemia, peripheral neuropathy, lung disease, liver disease, thyroid disorder, and human immunodeficiency virus infection

Discussion

Medication adherence is the main predictor of treatment success and an effective step in controlling BP. The present study showed that the overall incidence of patients' medication adherence was 191 (46.6%). The finding was almost similar to the WHO report in 2011 in developing countries which was 50% [38]. It was, also closely similar to studies done in Palestinian (45.8%) and Saudi Arabia (46%) [39, 40]. However, it was lower than studies reported in Taiwan (53%), Sweden (63.1%), and China (53.4%) [41, 42, 43]. Variations in the studied populations, better health care and access to health facilities might be contributing to the adherence variation between the studies. Moreover, methods of measurement used to assess medication adherence also could be the reason for variation between the studies. For example, in Taiwan medication adherence was measured using the medication possession ratio (percentage of time that the patient had medication available to them during the follow-up period) whereas, in Sweden adherence was measured by using the Proportion of Days Covered (PDC) method.

The current finding also lower than the studies conducted in Gondar and Jimma university which reported 64.6% and 61.8%, respectively [44-45]. This variation might be due to inclusion criteria and adherence measurement scale. The study at Jimma university included patients that had at least one year follow up and the sample size (280 patients) was lower than this study. In Gondar university, adherence was measured using Morisky 4-item Medication Adherence Scale. On the other hand, the adherence level reported in this study was higher than the finding from Ghana and Nigeria (30.3%) and Iran (24%) [46-47]. This difference might be explained by studies done in Ghana and Nigeria were included hypertensive patients with depression in their study hence, psychiatric illnesses contribute to low medication adherence. Similarly, in Iran participants were selected from the rural area only and this might have contributed to medication nonadherence.

In this study, a significant association was observed between patients' current occupation and medication non-adherence. Merchant hypertensive patients were 2.46 times more likely to be non-adherent than civil servants. This finding is in line with studies done in Hong Kong China and Black Loin Specialized Hospital, Ethiopia [48-49]. This might be due to forgetfulness when they travel or leave home along with their medicine. Besides, they might be too busy to come to

the health facility for their pills and difficulty remembering to take all their medicine on time. Another important factor that influences patient compliance in this study was physical inactivity. Patients who had no regular physical activity were 1.63 times more likely to be non-adherent compared to patients who were physically active which, was consistent with finding from Iran [47]. The exact mechanism was unclear but might be that physically inactive hypertensive patients will have uncontrolled BP (57.2 % in this study) so that clinician may be prescribed a more complex treatment regimen. Also, uncontrolled blood pressure patients might be hopeless to take their medication and adhere to their treatment plan.

In addition, the presence of DM comorbidity was significantly associated with poor antihypertensive medication adherence. Hypertensive patients with DM comorbidity were 2.54 times more likely to be non-adherent compared to those with no co-morbidities. The finding was similar to a study conducted in South Korea, Hong Kong China, Saudi Arabia, and Gondar, Ethiopia [10,48, 40, 44]. Possibly this is explained by patients with co-morbidities who could suffer from serious complications and complex treatment regimens which were unfavorable conditions to adhere to their medications. Also, 27.4% of participants in this study had no formal education, might be difficult to adhere to treatment plan and taking on time with complex treatment

The proportion of clinicians' adherence to hypertension treatment guidelines found in this study was 44.2%. The result was slightly higher than the study conducted in Zewditu Memorial Hospital, Ethiopia (37.4%) [26] This difference might be due to the small sample size and most of the study participants (65.2%) were without co-morbid condition in Zewditu Memorial Hospital. However, it was lower than studies conducted in South Indian (65%), Malaysia (85.30%), Island (70.4%), and South Africa (51.9%) [21, 23, 37, 29]. This difference might be due to race difference, health care setting that is lack of laboratory and imaging facilities for further screening for target organ damage in our setting. In addition, it might be explained by the difference in doctors' profiles since the majority of doctors treating hypertensive patients in this study were general practitioners and residents. Six of them were general practitioners (GP), 4 residency year 2 (R2), 8 of them are residency year 3(R3 from the total of 25 doctors, respectively.

CCBs and thiazide diuretics are the recommended first-line medicines for hypertension of Africa origin as per JNC-8 guidelines. However, from this study clinicians prescribed ACEIs followed

by diuretics and CCBs (63.7%, 57.1%, 35.1%), respectively. Among hypertensive patients with DM, CHF or CHD the most prescribed class of drugs was ACEI (66.3%, 71.0%, and 65.8% respectively), whereas in CKD most prescribed drug was diuretics. The overall use of ACEIs was similar to studies conducted in Serbia (60.57%) and Mexico City (63.8%) [30-31]. Studies performed in the Eastern Central Region of Portugal, and rural tertiary hospitals in Nigeria showed that thiazide diuretics were the most frequently prescribed class of medication (67%, and 84.9%), respectively [29, 16]. This difference might be due to studies in Portugal and Nigeria used JNC-7 as a reference guideline in which is conservative toward the thiazide-type diuretics as initial therapy for most patients without compelling indication.

The result of this study showed that 135 (32.5%) patients were on mono-therapy regardless of the presence or absence of comorbidities, which was lower than the studies reported from Mexico (72.1%), Canada (56.3%), and Turkey (75.7%) [31-33]. The difference might be due to better health care help to achieve target BP with a single medication in these countries. On the other hand, the majority of the patients, 275(66.1%) were on combination therapy. The finding was consistent with the study conducted in Kenya (60%) [34]. It was also similar to a study conducted at Zewditu Memorial Hospital Ethiopia (70.8%) [26]. However, the finding of this study was higher than the study conducted in Malaysia (56.7%) [35]. The higher prescription rate of combination therapy in this study might be due to the low rate of BP control 42.8% in this study as compared to 84.6% in Malaysia.

From the total of 135 (32.5%) patients who were on mono-therapy ACEI, 61 (14.7%) was the most prescribed drug class. The result was closely similar to a study conducted in Kenya (20.2%) [34]. However, it was lower than the study conducted in Turkey (30.1%) [33]. The variation might be due to using different standard reference guidelines in which European guidelines recommend any class of drug as initial therapy. On contrary, the study conducted in Gondar hospital showed that thiazide diuretics were the most commonly prescribed mono-therapy (60.24%) [36]. This discrepancy might be because of the difference in level guidelines adherence (66.8% prescription based on JNC-8 guidelines) that recommended the use of diuretics for both mono and combination therapy. In addition, variation may be different in the presence of CKD co-morbidity, as only 3.8% of participants had CKD comorbidity in this study.

Two drugs regimen was prescribed in 46.4 % of the hypertensive patients. ACEI + diuretics, 75 (18.0%) was mostly used two-drug combination therapy. The finding was in line with a study conducted in Kenya (14.5%) [34]. A study conducted in India using the JNC-8 guideline showed that the most frequently prescribed two-drug combination was ARB +diuretics [25]. This variation might be due to the cost and easy accessibility of ARB in India. On the other hand, in the study conducted in Nigeria, CCB + diuretic was the most frequently used two-drug combinations (36.6%) [16]. this variation might be suggesting of doctor's preference to use CCBs combination as initial therapy. Besides, in Nigeria majority of the patients were elderly (mean age was 61.5±15.1 years) hence, CCBs preferable for older patients because of isolated SBP more prevalence due to vessel stiffness.

Three drug combination antihypertensive therapies accounted for 17.1% of all prescription, of which BB+ diuretics +ACEI was most frequent. The result was similar to studies conducted in Turkey, Kenya, and Gondar hospital, Ethiopia [34, 33, 36]. On the other hand, in India and Nigeria, the most frequently prescribed was CCBs+ ACEIs+ Diuretics [25, 16]. This difference might be due to variation in the prevalence of the types of co-morbidities. Four drugs regimen was prescribed in 11(2.6%) hypertensive patients, ACEI +diuretics +CCB+BB 7(1.7%) were most frequently prescribed. This was consistent with a study conducted at Zewditu Memorial Hospital, Ethiopia [26]. In contrast, study in Nigeria the most frequent quadruple-therapy diuretics +CCBs +ACEIs+ methyldopa [16]. This discrepancy might be because of the difference in inclusion criteria, in Nigeria patients with heart failure were excluded from the study; this might be the reason why the use of BBs was lower as compared to this study.

Limitations of the Study

There are some limitations to this study. The study was conducted in one facility, therefore; the findings may not be generalized to reflect the health care setting in Ethiopia. Only prescription and co-morbidity data were used to examine compliance to treatment guidelines, which may reliable. Finally, this study was unable to identify factors affecting clinicians' adherence to standard treatment guidelines but will be an interesting area for future research.

Conclusion

The rate of medication adherence among hypertensive patients was poor. Merchant, physical inactivity, and presence of DM co-morbidity were factors associated with poor medication adherence among hypertensive patients at Jimma University Medical center. Prescribing patterns of antihypertensive drugs were inconsistent with JNC- 8 standard hypertension treatment guideline. Angiotensin-converting enzyme inhibitors were the most frequently prescribed class of anti-hypertensive drugs in both monotherapy and combination therapy and the majority of participants were on combination therapy. Effective education for patients to improve medication adherence and regular training for prescribers on practical treatment guidelines is recommended to keep them up-to-date with current trends of hypertension treatment and for better treatment outcomes.

Abbreviation

BP: blood pressure, SBP: systolic blood pressure, DBP: Diastolic blood pressure, CHD: coronary heart disease, DM: diabetes mellitus, CKD: chronic kidney disease, CVD: cardiovascular diseases, IBM: international business machine, JNC-8: eight joint national committee, BB: beta-blocker, CCB: calcium channel blocker, ARB: angiotensin receptor blocker, ACEI: angiotensin-converting enzyme inhibitor, TD: thiazide diuretic, MMAS-8: Morisky medication adherence scale-8, AOR: adjusted odds ratio, COR: crude odds ratio.

Declarations

Acknowledgments: We thank the participants of the study.

Availability of data and materials: All relevant data are within the manuscript and and its supporting Information files.

Authors' contributions: Bekalu Kebede: concept and design, data acquisition, interpretation of data, manuscript preparation; Legese Chelkeba: concept and design, interpretation of data, manuscript preparation, manuscript revision, and manuscript review. Bekalu Dessie: interpretation of data, manuscript preparation, manuscript revision, and manuscript review. All authors read and approved the final manuscript

Consent for publication: Not applicable.

Competing interests: The authors declare that they have no competing interests

References

1. Catherine P. Benziger GA. Roth AE. The Global Burden of Disease Study and the Preventable Burden of NCD. Journal of global heart.2016;11(4):393-397
2. Bromfield S, Muntner P. high blood pressure: the leading global burden of disease risk factor and the need for worldwide prevention programs. Curr Hypertens Rep. 2013;15(3):134-6.
3. Belue R, Okoror TA, Iwelunmor J, Taylor KD, Degboe AN, Agyemang C, et al. Globalization and Health An overview of cardiovascular risk factor burden in sub-Saharan African countries: a socio-cultural perspective. 2009; 12:1–12
4. Kibret KT, Mesfin YM. Prevalence of hypertension in Ethiopia: a systematic meta-analysis. Public Health Rev. 2015; 36:14.
5. Molla M. Systematic Reviews of Prevalence and Associated Factors of Hypertension in Ethiopia: Finding the Evidence. *Science Journal of Public Health*. 2015,3(4), 514-519.
6. Sarki AM, Nduka CU, Stranges S, Kandala NB, Uthman OA. Prevalence of Hypertension in Low- and Middle-Income Countries: A Systematic Review and Meta-Analysis. Medicine (Baltimore). 2015;94(50): e1959.
7. Ibrahim MM, Damasceno A. Hypertension in developing countries. Lancet. 2012;380(9841):611-9.
8. Grassi G, Cifkova R, Laurent S, Narkiewicz K, Redon J, Farsang C, et al. Blood pressure control and cardiovascular risk profile in hypertensive patients from central and eastern European countries. Eur Heart J. 2011; 32, 218–225
9. Schmieder RE, Mozaffarian D, Véronique L, Emelia J, Jarett D, Michael J et al. End Organ Damage in Hypertension. Circulation;2014; 129(3):1-19.
10. Shin S, Song H, Sang-Kwon Oh, Eob KC, Kim H, Jang S. Effect of antihypertensive medication adherence on hospitalization for cardiovascular disease and mortality in hypertensive patients. Hypertension Research.2013; 36(11):1000-1005.

11. Stevens PE, Levin A. Annals of Internal Medicine Clinical Guideline Evaluation and Management of Chronic Kidney Disease: Synopsis of Kidney Disease. 2013;825–31.

12. Ettehad D, Emdin CA, Kiran A, Anderson SG, Callender T, Emberson J, et al. Blood pressure lowering for prevention of cardiovascular disease and death: a systematic review and meta-analysis. Lancet, 2016; 387(10022), 957–967.

13. Jellinger PS, Handelsman Y, Rosenblit PD, Bloomgarden ZT, Fonseca VA, Garber AJ, Grunberger G, etal. AMERICAN ASSOCIATION OF CLINICAL ENDOCRINOLOGISTS AND AMERICAN COLLEGE OF ENDOCRINOLOGY GUIDELINES FOR MANAGEMENT OF DYSLIPIDEMIA AND PREVENTION OF CARDIOVASCULAR DISEASE. Endocr Pract. 2017 Apr;23(Suppl 2):1-87

14. James PA, Oparil S, Carter BL, Cushman WC, Dennison-Himmelfarb C, Handler J etal. 2014 Evidence-Based Guideline for the Management of high blood pressure in adults report from the panel members appointed to the eighth Joint National Committee (JNC 8). JAMA. 2014;311(5):507-20

15. Gebrihet TA, Mesgna KH, Gebregiorgis YS, Kahsay AB, Weldehaweria NB, Weldu G. Awareness, treatment, and control of hypertension is low among adults in Aksum town, northern Ethiopia: A sequential quantitative-qualitative study. PLoS One. 2017;12(5):e0176904.

16. Busari OA, Oluyombo R, Fasae AJ, Gabriel EO, Ayodele LM, Agboola SM. Prescribing pattern and utilization of antihypertensive drugs and blood pressure control in adult patients with systemic hypertension in a rural tertiary hospital in Nigeria. Am J Inter Med. 2014;2(6):144-9

17. Parati G, Omboni S, Compare A, Grossi E, Callus E, Venco A, et al. Blood pressure control and treatment adherence in hypertensive patients with metabolic syndrome: protocol of a randomized controlled study based on home blood pressure telemonitoring vs. conventional management and assessment of psychological determinants of adherence(TELEBPMET Study). trials; 2013;1–11.

18. Asgedom SW, Gudina EK, Desse TA. Assessment of Blood Pressure Control among Hypertensive Patients in Southwest Ethiopia. PLoS One. 2016;11(11):e0166432.

19. Papatya Karakurt, Mağfiret Kaşikçi, Factors affecting medication adherence in patients with hypertension, Journal of Vascular Nursing. 201230(4), 118-126,

20. Rolnick SJ, Pawloski PA, Hedblom BD, Asche SE, Bruzek RJ. Patient characteristics associated with medication adherence. Clin Med Res. 2013;11(2):54-65.

21. Datta S. Utilization Study of Antihypertensives in a South Indian Tertiary Care Teaching Hospital and Adherence to Standard Treatment Guidelines. J Basic Clin Pharm. 2016;8(1):33-37.

22. Mutua EM, Gitonga MM, Mbuthia B, Muiruri N, Cheptum JJ, Maingi T. Level of blood pressure control among hypertensive patients on follow-up in a regional referral hospital in Central Kenya. Pan Afr Med J. 2014;18:278.

23. Abdulameer SA, Sahib MN, Aziz NA, Hassan Y, Alrazzaq HA, Ismail O. Physician adherence to hypertension treatment guidelines and drug acquisition costs of antihypertensive drugs at the cardiac clinic: a pilot study. Patient Prefer Adherence. 2012;6:101-8.

24. Gudina EK, Michael Y, Assegid S. Prevalence of hypertension and its risk factors in southwest Ethiopia: a hospital-based cross-sectional survey. Integr Blood Press Control. 2013;6:111-7.

25. Romday R, Gupta A, Bhambani P. An assessment of antihypertensive drug prescription patterns and adherence to joint national committee-8 hypertension treatment guidelines among hypertensive patients attending a tertiary care teaching hospital. International Journal of Research in Medical Sciences. 2016;4(12).5125-33.

26. Yazie D, Shibeshi W, Woldu M, Berha A. Assessment of Blood Pressure Control among Hypertensive Patients in Zewditu Memorial Hospital, Addis Ababa, Ethiopia: A Cross-Sectional Study. Journal of Bioanalysis & Biomedicine. 2018;10.1-53

27. Pittrow D, Kirch W, Bramlage P, Lehnert H, Höfler M, Unger T et al. Patterns of antihypertensive drug utilization in primary care. Eur J Clin Pharmacol. 2004; 60(2):135-42.

28. Morgado MP, Rolo SA, Pereira L, Castelo-Branco M. Blood pressure control and antihypertensive pharmacotherapy patterns in a hypertensive population of Eastern Central Region of Portugal. BMC Health Serv Res. 2010;10:349.

29. Adedeji AR, Tumbo J, Govender I. Adherence of doctors to a clinical guideline for hypertension in Bojanala district, North-West Province, South Africa. Afr J Prim Health Care Fam Med. 2015;7(1):776.

30. Tomas A, Tomić Z, Milijasević B, Ban M, Horvat O, Vukmirović S et al. Patterns of prescription antihypertensive drug utilization and adherence to treatment guidelines in the city of Novi Sad. Vojnosanit Pregl. 2016;73(6):531-7.

31. Alba-Leonel A, Carvajal A, Fierro I, Castillo-Nájera F, Campos-Ramos O, Villa-Romero A et al. Prescription patterns of antihypertensives in a community health centre in Mexico City: a drug utilization study. Fundam Clin Pharmacol. 2016; 30(3):276-81.

32. Beaulieu MD, Dufresne L, LeBlanc D. Treating hypertension. Are the right drugs given to the right patients? Can Fam Physician. 1998; 44:294-8, 301-2.

33. Abaci A, Kozan O, Oguz A, Sahin M, Deger N, Senocak H et al. Prescribing pattern of antihypertensive drugs in primary care units in Turkey: results from the TURKSAHA study. Eur J Clin Pharmacol. 2007 Apr;63(4):397-402.

34. Mbui JM, Oluka MN, Guantai EM, Sinei KA, Achieng L, Baker A et al. Prescription patterns and adequacy of blood pressure control among adult hypertensive patients in Kenya; findings and implications. Expert Rev Clin Pharmacol. 2017; 10(11):1263-1271.

35. Ramli A, Miskan M, Ng K, Ambigga D, Nafiza M, Mazapuspavina M, Sajari J, Ishak R. Prescribing of Antihypertensive Agents in Public Primary Care Clinics - is it in Accordance with Current Evidence? Malays Fam Physician. 2010;5(1):36-40.

36. Abegaz TM, Tefera YG, Abebe TB. Antihypertensive drug prescription patterns and their impact on outcome of blood pressure in Ethiopia: a hospital-based cross-sectional study. Integr Pharm Res Pract. 2017; 6:29-35.

37. Theodorou M, Stafylas P, Kourlaba G, Kaitelidou D, Maniadakis N, Papademetriou V. Physicians' perceptions and adherence to guidelines for the management of hypertension: a national, multicentre, prospective study. Int J Hypertens. 2012;2012:503821.

38. Brown MT, Bussell JK. Medication adherence: WHO cares? Mayo Clin Proc. 2011; 86(4):304-14.

39. Al-Ramahi R. Adherence to medications and associated factors: A cross-sectional study among Palestinian hypertensive patients. J Epidemiol Glob Health. 2015; 5(2):125-32.

40. Khayyat SM, Khayyat SM, Hyat Alhazmi RS, Mohamed MM, Abdul Hadi M. Predictors of Medication Adherence and Blood Pressure Control among Saudi Hypertensive Patients Attending Primary Care Clinics: A Cross-Sectional Study. PLoS One. 2017;12(1):e0171255.

41. Lee CY, Huang CC, Shih HC, Huang KH. Factors influencing antihypertensive medication compliance in Taiwan: a nationwide population-based study. Eur J Prev Cardiol. 2013; 20(6):930-7.

42. Hedna K, Hakkarainen KM, Gyllensten H, Jönsson AK, Andersson Sundell K, Petzold M, Hägg S. Adherence to Antihypertensive Therapy and Elevated Blood Pressure: Should We Consider the Use of Multiple Medications? PLoS One. 2015;10(9):e0137451.

43. Li YT, Wang HHX, Liu KQL, Lee GKY, Chan WM, Griffiths SM et al. Medication Adherence and Blood Pressure Control Among Hypertensive Patients With Coexisting Long-Term Conditions in Primary Care Settings: A Cross-Sectional Analysis. Medicine (Baltimore). 2016; 95(20):e3572.

44. Ambaw AD, Alemie GA, W/Yohannes SM, Mengesha ZB. Adherence to antihypertensive treatment and associated factors among patients on follow up at University of Gondar Hospital, Northwest Ethiopia. BMC Public Health. 2012;12:282.

45. Asgedom SW, Atey TM, Desse TA. Antihypertensive medication adherence and associated factors among adult hypertensive patients at Jimma University Specialized Hospital, southwest Ethiopia. BMC Research Notes. 2018;11(1):27.

46. Boima V, Ademola AD, Odusola AO, Agyekum F, Nwafor CE, Cole H, et al. Factors Associated with Medication Nonadherence among Hypertensives in Ghana and Nigeria. Int J Hypertens. 2015;2015:205716.

47. Kamran A, Sadeghieh Ahari S, Biria M, Malepour A, Heydari H. Determinants of Patient's Adherence to Hypertension Medications: Application of Health Belief Model Among Rural Patients. Ann Med Health Sci Res. 2014; 4(6):922-7.

48. Kang CD, Tsang PP, Li WT, Wang HH, Liu KQ, Griffiths SM et al. Determinants of medication adherence and blood pressure control among hypertensive patients in Hong Kong: a cross-sectional study. Int J Cardiol. 2015 Mar 1;182:250-7.

49. Hareri HA, Abebe M and Asefaw T: Assessments of adherence to Hypertension managements and its influencing factors among Hypertensive patients attending Black Lion Hospital chronic follow up unit, Addis Ababa, Ethiopia-a cross-sectional study. *Int J Pharm Sci Res* 2013; 4(3); 1086-1095.

First, we would like to appreciate the editor and reviewers for giving us another round of invaluable comments so as to revise our manuscript accordingly. We have gone through all the comments given by the reviewers and revised the manuscript point by point.

Response to editors /editorial office

S. no	Comments/concerns	Response to comments
1	Please ensure that your manuscript meets PLOS ONE's style requirements, including those for file naming.	We tried to prepare and re-write our manuscript based on PLOSE ONE manuscript body formatting guidelines.
2	Please include additional information regarding the survey or questionnaire used in the study and ensure that you have provided sufficient details that others could replicate the analyses. For instance, if you developed a questionnaire as part of this study and it is not under a copyright more restrictive than CC-BY, please include a copy, in both the original language and English, as Supporting Information. Moreover, please include more details on how the questionnaire was pre-tested, and whether it was validated.	Questionnaire used in the study was developed by reviewing different literatures from similar studies. Pre-test of data collection format was performed on 5% (21) of the sample before conducting the study in one of local hospital found in Ethiopia which is specialized teaching hospital like that of study area. Then, the final tool was developed with some modifications after reviewing the results of the pre-test. The questionnaire was validated by an expert in the field of pharmacy practice for length, readability, and relevance.
3	Please provide additional details regarding participant consent. In the ethics statement in the Methods and online submission information, please ensure that you have specified how verbal consent was documented and witnessed.	This study was approved by the Ethical Review Board of School of Pharmacy, Institute of Health Science Jimma university (Ref.No IHRPGD/203/18). Prior to data collection, individual informed verbal consent was obtained from the study participants. Written informed consent was waived since the study did not involve any procedure and present no damage to patients as approved by the ethical review board committee of Board of School of Pharmacy, Institute of Health Science Jimma university. Patients were informed about the objective of the study; procedures of selection and assurance of

		confidentiality and their names was not registered to minimize social desirability bias and enhance anonymity. They were not forced to participate or receive any monetary incentive and it was solely voluntary based.
4	We suggest you thoroughly copyedit your manuscript for language usage, spelling, and grammar. If you do not know anyone who can help you do this, you may wish to consider employing a professional scientific editing service.	The manuscript was extensively revised by a person who is expert in the field of English language
5	Please remove any funding-related text from the manuscript	Done
6	Your ethics statement should only appear in the Methods section of your manuscript. If your ethics statement is written in any section besides the Methods, please move it to the Methods section and delete it from any other section. Please ensure that your ethics statement is included in your manuscript, as the ethics statement entered into the online submission form will not be published alongside your manuscript.	????

Response to additional comments given

S. No	Comments	Response by authors
1	Is the manuscript technically sound, and do the data support the conclusions?	Authors tried to revised the manuscript, rearrange and correct some technical issue such as editorial problem in extensive manner. In addition we attempted to present key finding with available evidence from a given data and come up with conclusion accordingly.

2	Has the statistical analysis been performed appropriately and rigorously?	Authors strongly believed that this study carried out by using appropriate statically analysis methods and the finding interpreted in scientific way.
3	Have the authors made all data underlying the findings in their manuscript fully available?	Authors attached additional figures and tables as supporting information
3	Is the manuscript presented in an intelligible fashion and written in standard English?	As we mentioned above the manuscript was revised extensively with a person who is expert in English language
4	Age classification 60 and >60 seems to be very abstract and does not talk much about the study population<br>It would be advisable to classify patients into age groups so that the reader can easily understand the patient demographic	This classification system is based on JNC-8 hypertension treatment guideline for goal of blood pressure management

Response to Reviewer 1

S. No	Comments	Response
1	Authors need to rework on the manuscript for better clarity. The language is not clear. The methodology is also not robust. The number of tables are also more withlengthy descriptions. Discussion section also requires rewriting and conclusion has to be based upon the findings and the possible recommendations	As per comment given, a whole manuscript was revised including language, methodology, tables and figures. Discussion and<br>recommendation also rewrite based upon the key finding
**Response to Reviewer 2**		
1	**Introduction**<br>There are a lot of issues here. For example, you mentioned on page 3 that "Ethiopia has not its own hypertension treatment guideline and on the time being this guideline widel yusing by both clinicians and clinical pharmacist [16]". What exactly do you mean? The sentence is confusing	That means, Ethiopia has not its own hypertension treatment guideline. Therefore, JNC-8 guideline is widely used by both clinicians and clinical pharmacist in Ethiopia [16].
2	**Method** Please what do you mean by patients were consequently followed for the next three months? Was done done daily, weekly, monthly, etc for all participants (page 5)? Your sample size was calculated based on patients' adherence to treatment; do you think that this	Only those patients selected at first month were consequently followed every month for the next three regarding their life stay modification medication adherence status and what medication prescribed for them. In this study all clinicians who treated

	will adequately cater for physicians adherence to guidelines? Page 5, please what do you mean by "questioner"? Please check your definition of Guideline adherence. I think the denominator "total number of hypertensive patients treated". I also think that the Operational Definition should beat the Appendix and referred to periodically. Page 5, please how were your BP measurements taken?	hypertensive patients during study period were included. Questioner is to mean questionnaire it is spelling error<br>At Jimma University ambulatory cardiac center, initial and subsequent BP typically measured using the cuff sphygmomanometer and digital sphygmomanometer on the left arm, at the level of the heart of the patient, who was in a sitting position and at rest for at least 5 minute. This<br>information typically recorded in the patients' notes
3	**Results**<br>Your tables, in particular table 6 should be reformatted. It is very difficult to following the distributions.<br>Table 3, please why do you put those with one medication under co-medication? Enalapril constitutes 63.7% of ACEIs, where are the rest? Same with ARBs. Why have you presented ACEIs and ARBs different from Diuretics, CCBs and BBs?<br>Table 6 is not clear at all. I could not read this well, but there are issues with it.	Table 6 re- arranged by presented only variables that were statically significant for poor medication adherence. In table 3 co-medication means adjuvant or other medication other than anti-hypertensive such as statins, anti-platelet, anti-diabetes etc.<br>Table 3 showed Frequency of all antihypertensive medicines prescribed for hypertensive patients. Medications/class of medication presented separately because of JNC-8 guideline recommendation for white and black race (Africa origin) population. Initial treatment for nonblack population thiazide type diuretics (TDs) or angiotensin converting enzyme inhibitors (ACEIs) or angiotensin receptor blockers (ARBs) or calcium channels blockers (CCBs), alone or in combination and for black population, TDs or CCBs, alone or in combination

4	**Conclusion** Because most of your information are on Table 6 and I could not read this well, I did not review your discussion..	Authors accept a given comment and correct acordingly
**Response for Reviewer 3**		
1	**Reviewer #3**: There are many grammatical errors in the manuscript that tend to lose the reader. They have been included in the reviewed manuscript uploaded. The manuscriptlooks like it has been copy pasted from a dissertation/thesis; only key information should be included and the rest uploaded. These sections have been highlighted in theattached manuscript. There are very minimal results on the clinicians' adherence to treatment guidelines and these results are not discussed, yet they seem to form a huge partof this manuscript. In the discussion section, the authors should discuss only the key findings and not every finding and include the implications of such findings. This has been scantily done. There are too many tables and figures in the manuscript- the authors should include only key findings and present the rest as attachments. The authors should review author guidelines and revise the manuscript accordingly**.**	Authors accept all comments given by reviewer ≠3 and attempted to correct accordingly.

Thank you
