# Supplementary material for: Clinicians’ prescribing pattern, rate of patients’ medication adherence and its determinants among adult hypertensive patients at Jimma University Medical Center: Prospective cohort study
Source: PLoS One. 2021 Nov 15;16(11):e0259421. doi: 10.1371/journal.pone.0259421 (PMC8592482; doi:10.1371/journal.pone.0259421)
Supplement: Attachment — Submitted filename: PONE-D-20-38133_R2.pdf [file pone.0259421.s008.pdf]

PLOS ONE

Clinicians' Prescribing Pattern, Rate of Patients' Medication Adherence and Its Determinants among Adult Hypertensive Patients at Jimma University Medical Center: Prospective Cohort Study

--Manuscript Draft--

**Manuscript Number:**	PONE-D-20-38133R2
**Article Type:**	Research Article
**Full Title:**	Clinicians' Prescribing Pattern, Rate of Patients' Medication Adherence and Its Determinants among Adult Hypertensive Patients at Jimma University Medical Center: Prospective Cohort Study
**Short Title:**	Clinician's Prescribing Pattern, Rate of Patients' Medication Adherence and Its Determinants
**Corresponding Author:**	Bekalu Kebede simegn, MSc<br>Debremarkos Uiversity<br>Debre Markos, Amhara ETHIOPIA
**Keywords:**	Prescribing Pattern, Hypertension, Blood Pressure Control, Guidelines, Adherence
**Abstract:**	Abstract<br><br>Background : Many studies conducted in the past focused on patients' sociodemographic factors and medical profiles to identify the determinants of suboptimal blood pressure control. However, prescribing patterns and clinicians' adherence to guidelines are also important factors affecting the rate of blood pressure control. Therefore, this study aimed to determine clinicians' prescribing patterns, patients' medication adherence, and its determinants among hypertensive patients at Jimma University Medical Center.<br>Methods : A general prospective cohort study was conducted among hypertensive patients who had regular follow-up at Jimma university ambulatory cardiac clinic from March 20, 2018, to June 20, 2018. Patients' specific data was collected with a face-to-face interview and from their medical charts. Clinicians' related data were collected through a self-administered questionnaire. Data were analyzed using SPSS version 21.0. Bivariate and multivariable logistic regression analyses were done to identify key independent variables influencing patients' adherence. P-Values of less than 0.05 were considered statically significant.<br>Results : From the total of 416 patients, 237(57.0%) of them were males with a mean age of 56.50 ± 11.96 years. Angiotensin-converting enzyme inhibitors were the most frequently prescribed class of antihypertensives, accounting for 261(63.7%) prescriptions. Combination therapy was used by the majority of patients, with 275 (66.1%) patients receiving two or more antihypertensive drugs. Patients' medication adherence was 46.6%, while clinicians' guideline adherence was 44.2%. Patients with merchant occupation (P=0.020), physical inactivity (P=0.033), and diabetes mellitus co-morbidity (P=0.008) were significantly associated with a higher rate of medication non-adherence.<br>Conclusion : The rate of medication adherence was poor among hypertensive patients. Physicians were not adherent to standard treatment guideline. The most commonly prescribed class of drugs were angiotensin-converting enzyme inhibitors. Effective education should be given to patients to improve medication adherence. Prescribers should be trained on treatment guidelines regularly to keep them up-to-date with current trends of hypertension treatment and for better treatment outcomes
**Order of Authors:**	Bekalu Kebede simegn, MSc
	Bekalu Dessie Alamirew
	Legese chelkeba
**Response to Reviewers:**	we have tried to address all comments given by reviewers
**Additional Information:**	
**Question**	**Response**

Powered by Editorial Manager® and ProduXion Manager® from Aries Systems Corporation

**Financial Disclosure**<br><br>Enter a financial disclosure statement that describes the sources of funding for the work included in this submission. Review the submission guidelines for detailed requirements. View published research articles from *PLOS ONE* for specific examples.<br><br>This statement is required for submission and **will appear in the published article** if the submission is accepted. Please make sure it is accurate.<br><br>**Unfunded studies**<br>Enter: *The author(s) received no specific funding for this work.*<br><br>**Funded studies**<br>Enter a statement with the following details:<br>• Initials of the authors who received each award<br>• Grant numbers awarded to each author<br>• The full name of each funder<br>• URL of each funder website<br>• Did the sponsors or funders play any role in the study design, data collection and analysis, decision to publish, or preparation of the manuscript?<br>• **NO** - Include this sentence at the end of your statement: *The funders had no role in study design, data collection and analysis, decision to publish, or preparation of the manuscript.*<br>• **YES** - Specify the role(s) played.<br><br>* typeset	This research was funded by Jimma University, College of Health Sciences. The University has no role in designing, conducting and reporting of the study.
**Competing Interests**<br><br>Use the instructions below to enter a competing interest statement for this submission. On behalf of all authors, disclose any competing interests that could be perceived to bias this work—acknowledging all financial support and any other relevant financial or non-financial competing interests.<br><br>This statement **will appear in the published article** if the submission is accepted. Please make sure it is	The authors declared that no competing interests exist.

accurate. View published research articles from *PLOS ONE* for specific examples.<br><br>**NO authors have competing interests**<br><br>Enter: *The authors have declared that no competing interests exist.*<br><br>**Authors with competing interests**<br><br>Enter competing interest details beginning with this statement:<br><br>*I have read the journal's policy and the authors of this manuscript have the following competing interests: [insert competing interests here]*<br><br>* typeset	
**Ethics Statement**<br><br>Enter an ethics statement for this submission. This statement is required if the study involved:<br><br>• Human participants<br>• Human specimens or tissue<br>• Vertebrate animals or cephalopods<br>• Vertebrate embryos or tissues<br>• Field research<br><br>Write "N/A" if the submission does not require an ethics statement.<br><br>General guidance is provided below. Consult the submission guidelines for detailed instructions. **Make sure that all information entered here is included in the Methods section of the manuscript.**	Ethics approval and consent to participate: This study was approved by the Ethical Review Board of School of Pharmacy, Institute of Health Science Jimma University (Ref.No IHRPGD/203/18). First, each participant was asked orally whether they were voluntary or not to participate in this study after a detailed explanation of the objective of the study, procedures of selection, and assurance of confidentiality. An independent data collection supervisor acted as witness for voluntary informed decision making of participants to take part in the study. Written informed consent was waived since the study did not involve any procedure and present no damage to patients as approved by the ethical review board committee of the Board of School of Pharmacy, Institute of Health Science Jimma University. Their names were not registered to minimize social desirability bias and enhance anonymity. They were not forced to participate or receive any monetary incentive and it was solely voluntary based.

**Format for specific study types**<br><br>**Human Subject Research (involving human participants and/or tissue)**<br>• Give the name of the institutional review board or ethics committee that approved the study<br>• Include the approval number and/or a statement indicating approval of this research<br>• Indicate the form of consent obtained (written/oral) or the reason that consent was not obtained (e.g. the data were analyzed anonymously)<br><br>**Animal Research (involving vertebrate animals, embryos or tissues)**<br>• Provide the name of the Institutional Animal Care and Use Committee (IACUC) or other relevant ethics board that reviewed the study protocol, and indicate whether they approved this research or granted a formal waiver of ethical approval<br>• Include an approval number if one was obtained<br>• If the study involved *non-human primates*, add *additional details* about animal welfare and steps taken to ameliorate suffering<br>• If anesthesia, euthanasia, or any kind of animal sacrifice is part of the study, include briefly which substances and/or methods were applied<br><br>**Field Research**<br><br>Include the following details if this study involves the collection of plant, animal, or other materials from a natural setting:<br>• Field permit number<br>• Name of the institution or relevant body that granted permission	
**Data Availability**<br><br>Authors are required to make all data underlying the findings described fully available, without restriction, and from the time of publication. PLOS allows rare exceptions to address legal and ethical concerns. See the PLOS Data Policy and FAQ for detailed information.	No - some restrictions will apply

A Data Availability Statement describing where the data can be found is required at submission. Your answers to this question constitute the Data Availability Statement and **will be published in the article**, if accepted.<br><br>**Important:** Stating 'data available on request from the author' is not sufficient. If your data are only available upon request, select 'No' for the first question and explain your exceptional situation in the text box.<br><br>Do the authors confirm that all data underlying the findings described in their manuscript are fully available without restriction?	
**Describe where the data may be found in full sentences. If you are copying our sample text, replace any instances of XXX with the appropriate details.**<br><br>• If the data are **held or will be held in a public repository**, include URLs, accession numbers or DOIs. If this information will only be available after acceptance, indicate this by ticking the box below. For example: *All XXX files are available from the XXX database (accession number(s) XXX, XXX.*).<br>• If the data are all contained **within the manuscript and/or Supporting Information files**, enter the following: *All relevant data are within the manuscript and its Supporting Information files.*<br>• If neither of these applies but you are able to provide **details of access elsewhere**, with or without limitations, please do so. For example:<br><br>*Data cannot be shared publicly because of [XXX]. Data are available from the XXX Institutional Data Access / Ethics Committee (contact via XXX) for researchers who meet the criteria for access to confidential data.*<br><br>*The data underlying the results presented in the study are available from (include the name of the third party*	All relevant data are within the manuscript and its Supporting Information files

and contact information or URL).<br>• This text is appropriate if the data are owned by a third party and authors do not have permission to share the data.<br><br>* typeset	
Additional data availability information:	Tick here if the URLs/accession numbers/DOIs will be available only after acceptance of the manuscript for publication so that we can ensure their inclusion before publication.

Powered by Editorial Manager® and ProduXion Manager® from Aries Systems Corporation

Manuscript Click here to access/download;Manuscript;Manuscript.docx

Clinicians' Prescribing Pattern, Rate of Patients' Medication Adherence and Its Determinants among Adult Hypertensive Patients at Jimma University Medical Center: Prospective Cohort Study

Bekalu Kebede, MSc[1*], Legese Chelkeba, PhD[2], Bekalu Dessie, MSc[1]

[1] Department of Pharmacy, College of Health Sciences, Debre Markos University, Debre Markos, Ethiopia

[2]Department of Clinical Pharmacy, School of Pharmacy, College Health Sciences, Addis Ababa University, Addis Ababa, Ethiopia

***Corresponding author: Bekalu Kebede**

Clinical Pharmacist, Department of Pharmacy, College of Health Sciences, Debre Markos University, Debre Markos, Ethiopia

Phone: +251-921-286-140

Email: bekalukebede19@gmail.com

Abstract

Background: Many studies conducted in the past focused on patients' sociodemographic factors and medical profiles to identify the determinants of suboptimal blood pressure control. However, prescribing patterns and clinicians' adherence to guidelines are also important factors affecting the rate of blood pressure control. Therefore, this study aimed to determine clinicians' prescribing patterns, patients' medication adherence, and its determinants among hypertensive patients at Jimma University Medical Center.

Methods: A general prospective cohort study was conducted among hypertensive patients who had regular follow-up at Jimma university ambulatory cardiac clinic from March 20, 2018, to June 20, 2018. Patients' specific data was collected with a face-to-face interview and from their medical charts. Clinicians' related data were collected through a self-administered questionnaire. Data were analyzed using SPSS version 21.0. Bivariate and multivariable logistic regression analyses were done to identify key independent variables influencing patients' adherence. P-Values of less than 0.05 were considered statically significant.

Results: From the total of 416 patients, 237(57.0%) of them were males with a mean age of 56.50 ± 11.96 years. Angiotensin-converting enzyme inhibitors were the most frequently prescribed class of antihypertensives, accounting for 261(63.7%) prescriptions. Combination therapy was used by the majority of patients, with 275 (66.1%) patients receiving two or more antihypertensive drugs. Patients' medication adherence was 46.6%, while clinicians' guideline adherence was 44.2%. Patients with merchant occupation (P=0.020), physical inactivity (P=0.033), and diabetes mellitus co-morbidity (P=0.008) were significantly associated with a higher rate of medication non-adherence.

Conclusion: The rate of medication adherence was poor among hypertensive patients. Physicians were not-adherent to standard treatment guideline. The most commonly prescribed class of drugs were angiotensin-converting enzyme inhibitors. Effective education should be given to patients to improve medication adherence. Prescribers should be trained on treatment guidelines regularly to keep them up-to-date with current trends of hypertension treatment and for better treatment outcomes.

Keywords: Prescribing Pattern, Blood Pressure Control, Guidelines, Adherence

Introduction

Hypertension is a significant public health challenge in the world because of its high prevalence and concomitant risks of cardiovascular and kidney diseases [1]. According to the World Health Organization (WHO), high blood pressure (BP) is a major public health problem that kills one in every eight people and is the world's third-leading silent killer [2].

Almost three-quarters of hypertensive people (639 million people) live in countries with limited health resources [1, 2]. In Sub- Saharan Africa, it is a major independent risk factor for heart failure, stroke, and kidney failure. These complications arise as a result of a low rate of hypertension diagnosis, poor BP control, high morbidity and mortality, and low resources in health care settings [3]. A systematic review and meta-analysis study conducted in Ethiopia in 2015 estimated the prevalence of hypertension to be 19.6 % [4]. Another systematic review and meta-analysis conducted in Ethiopia in 2015 found that the prevalence of hypertension ranged from 20% and 30% [5].

Despite the fact that hypertension is a preventable and modifiable risk factor for cardiovascular diseases (CVD), the prevention and control of hypertension have not received attention in many developing countries [6,7]. It accounts for 40.6% of deaths due to all coronary heart disease and 38.5% of deaths due to stroke [8]. It is also the second-leading cause of end-stage renal disease (ESRD) after diabetes mellitus (DM [9].

Guideline adherence is defined as a condition in which the prescribed treatment obeys treatments recommended in the identified practice guidelines [10-12]. Hypertension guidelines are evidence-based and are usually dictated by randomized controlled trial data and observational studies. Published guidelines aid in clinical decision making, decrease practice variations, guide correctness, and measure the quality of health care [13-14]. The Eighth Joint National Committee (JNC-8) on detection, evaluation, and treatment of BP is most the commonly used guideline and it is considered as a “gold standard” consensus guideline for the management of hypertension [15]. This guideline was used as a reference standard for this study because awareness and accessibility of guidelines were initial criteria to evaluate the status of clinicians’ adherence to hypertension treatment guidelines. Besides, Ethiopia does not have a hypertension treatment guideline. Therefore, the JNC-8 guideline is widely used by both clinicians and clinical pharmacists in Ethiopia [16]. Clinical practice guidelines do not

consistently change clinicians' behavior, control of high blood pressure remains suboptimal, so creating the need to evaluate adherence to guidelines and its impact on BP control [10].

Many studies conducted in the past to explore the causes of suboptimal BP control focused on patient factors such as socio-demographic, medical profile, and patient's treatment compliance [17-20]. However, prescribing patterns and clinicians’ adherence to guidelines are also important factors affecting hypertension treatment outcomes [21-24].

Several studies were conducted worldwide using either prescription or drug dispensing data to evaluate prescription patterns and clinicians’ adherence to hypertension treatment guidelines [25-27]. But few data are available in Ethiopia. Also, among previous studies, there were conflicting results between prescribing patterns and patients’ medication adherence. A study conducted in Kenya concluded that combination therapies were associated with poor patient adherence [22]. In contrast, in a study conducted in Nigeria combination therapies were associated with good blood pressure controlled than mono-therapy [16]. A study conducted at Jimma University, Ethiopia showed that the number of antihypertensive medications prescribed was not associated with blood pressure control status [18]. Besides, most previous studies used a similar study design(cross-sectional). As a result, a general prospective cohort study was conducted among ambulatory hypertension patients to determine prescribing patterns of antihypertensive drugs, patients’ medication adherence, and its determinants.

Methods

Study period and setting

This study was conducted over three months among hypertensive patients who were regularly followed up every month at Jimma university ambulatory cardiac clinic from March 20, 2018, to June 20, 2018. Jimma University is the only teaching and specialized hospital in the Jimma zone. It serves over 15 million people living in Jimma city and its surrounding areas. It is located in Jimma town, Oromia Regional State, Ethiopia, 352 km southwest of Addis Ababa, the capital city of Ethiopia (18).

Study design: a general prospective cohort follow-up study was employed

Study population; All hypertensive patients with age ≥ 18 years, with a documented diagnosis, had at least two times medical appointments before the study period, having a medical appointment every month and complete medical records were included in the study. We

excluded pregnant women, patients with confirmed neurologic and psychiatric disorders with their respective specialists since it was challenging to assess medication adherence statuses in acutely ill patients with similar tools like that of other study participants.

Sample size and sampling technique: The sample size for this study was calculated using Cochran single proportion formula. A previous study conducted in Ethiopia reported that the rate of patients' medication adherence was 50 % [18]. Based on this formula minimum 384 sample size was calculated using a standard normal distribution (Z=1.96) with a confidence interval of 95% and a margin of error of 0.05. After adjusting the 10% non-respondent rate, 416 hypertensive patients were included in this study. All clinicians who treated hypertensive patients during the study period were included and a random sampling technique was used to select patients for inclusion in the study.

Data collection process and data collection tool

Questionnaire and data abstraction format was prepared by reviewing different literatures. To maintain the validity of the data collection tool, a structured questionnaire was developed and translated to local languages (Amharic and Afan Oromo) by native speakers of the respective languages, and then back-translated to the English version. A pre-test of the data collection format was performed on 5% of the sample size. Face-to-face interviews were use to collect primary data from patients, and a self-administered questionnaire was used to collect data from all clinicians who treat hypertensive patients during the study period. During each visit, secondary data such as prescribed drugs, blood pressure measurements, and co-morbid illness were collected from patients' charts. Initial and subsequent BP is typically measured at Jimma University Medical Center, using the cuff sphygmomanometer and digital sphygmomanometer on the left arm, at the level of the heart, in a sitting position, and at rest for at least 5 minutes. In this study, hypertensive patients who met the inclusion criteria were selected at the first month of the data collection period and BP measurements at this first visit were considered as a baseline. Then only those patients selected at the first month were consequently followed every month for the next three months. Morisky Medication Adherence Scale-8 (MMAS-8) was used to assess the patients' medication adherence. The MMAS-8 total score was calculated by summing the values from all 8 items, with reverse coding when necessary. A score of <2 (out of a full range of 8) was considered as good medication adherence, otherwise considered as poor adherence [19].

Pharmacological Trends Guideline Adherence Evaluation Method was used to measure clinicians' adherence to hypertension treatment guidelines [25]. A recommendation of the JNC-8 on the detection, evaluation, and treatment of high BP was used as a reference guideline to evaluate prescription patterns and clinicians' adherence to the guidelines.

Data processing and analysis: The data were entered using Epidata version 3.1 and exported to the Statistical Package for Social Science (SPSS) version 21.0. Continuous variables were presented as means (standard deviation). Categorical variables were presented as frequency and percentages. Antihypertensive medicines were categorized according to their therapeutic classes. Switch from one drug to another drug during each visit, the last regimen was included in the analysis unless changing was done at the last visit.

Guideline adherence was expressed as % = (Total number hypertensive patients treat based on JNC-8 guideline divide by the total number of participants) × 100 [23]. Based on this calculation, >65% conceder as complete adherence, 50 – 64.9% medium adherence, and <49.9% will be classified as low adherence [29]. Finally, BP was calculated by taking an average of three measurements and categorized controlled and uncontrolled based on guidelines. Prior to regression alalysis, a multi-collinearity test was done , and adequacy of cell distribution was checked using the chi-square test. Binary logistic regression was performed to determine the effect of each variable on patients' adherence and variables with a P-value less than 0.25 in the bivariate analysis were then included in a multivariate logistic regression analysis to identify key independent variables influencing patient's medication adherence. In multivariate analysis, variables with P- a value of < 0.05 were considered statistically significant.

Ethics approval and consent to participates: This study was approved by the Ethical Review Board of School of Pharmacy, Institute of Health Science, Jimma University (Ref.No IHRPGD/203/18). After a detailed explanation of the objective of the study, procedures of selection, and assurance of confidentiality, each participant were asked orally whether they are voluntary or not to participate in this study. An independent data collection supervisor acted as a witness for voluntary informed decision-making of participants to take part in the study. Written informed consent was waived since the study did not involve any procedure and presented no damage to patients as approved by the ethical review board committee of the Board of School of Pharmacy, Institute of Health Science Jimma University. Their names were not registered to minimize social

desirability bias and enhance anonymity. They were not forced to participate or receive any monetary incentive and it was solely voluntary based.

Results

Baseline characteristics of study participants

A total of 686 hypertensive patients visited the hypertension clinic during the study period. Four hundred fifty-nine patients fulfilled inclusion criteria, of these, 416 participants were included in the final analyses (Fig 1). More than half (57.0%) of the participants were males with a mean age of 56.50 ± 11.96 years (Table 1).

Table 1: Socio-demographic characteristics of hypertensive patients at JUMC, 2018

Variables	Characteristics	Frequency (%)
**Age (year)**	Mean ± SD	56.50±11.96
	<60	220(52.9)
	≥60	196(47.1)
**Sex**	Male	237 (57.0)
	Female	179 (43.0)
**BMI**	Mean ± SD	24.24±2.32
	18.5-24.9	314 (75.5)
	25-29.9	89 (21.4)
	≥30	13 (3.1)
**Marital status**	Single	12 (2.9)
	Married	284 (68.3)
	Divorced	55 (13.2)
	Widowed	65 (15.6)
**Residency**	Urban	274 (65.9)
	Rural	142 (34.1)
**Education level**	No formal education	114 (27.4)
	Primary education (1-8 grade)	110 (26.4)
	Secondary education (9-12 grade)	100 (24.0)
	Tertiary education (diploma & above)	92 (22.1)
**Current occupation**	Civil servant	69 (16.6)
	Merchant	89 (21.4)
	Farmer	74 (17.8)
	Housewife	47 (11.3)
	Retired	36 (8.7)
	Jobless	70 (16.8)
	Other	31 (7.5)
**Monthly income (ETB)**	<1000	58 (13.9)
	1001-2000	68 (16.3)
	2001-3000	74 (17.8)
	>3000	109 (26.2)

	Without unknown monthly income	107 (25.7)
**Living status**	Solo	59 (14.2)
	Live with family	328 (78.8)
	Others*	29 (7.0)

Others: Drivers, daily laborer, Non-governmental organization and private work; others*: Live with friends, prison; ETB: Ethiopian Birr

Two hundred sixty-four (63.5%) of participants have been taking salt with food. More than half (53.4%) of the participants were physically inactive and 138 (33.2%) patients were khat chewers. Among co-morbid conditions, 104(25.0 %) of participants had diabetes mellitus (DM), 38(9.1%) and 39(9.4%) had coronary heart disease (CHD) and dyslipidemia, respectively (Table 2).

Table 2: Lifestyle factors and clinical characteristics of hypertensive patients at JUMC, 2018

Factors	**Categories**	**Frequency (%)**
Add salt to food	Yes	264 (63.5)
	No	152 (36.5)
Alcohol use	Yes	103 (24.8)
	No	313 (75.2)
Chew khat	Yes	138 (33.2)
	No	278 (66.8)
Cigarette smoking	Never smoked	303 (72.8)
	Ex-smoker	93 (22.4)
	Current smoker	20 (4.8)
Physical activity	Physically active	194 (46.6)
	Physically inactive	222 (53.4)
Drink coffee	Yes	238 (57.2)
	No	178 (42.8)
Use traditional medicine	Yes	16 (3.8)
	No	400 (96.2)
Co morbidity	Hypertension alone	158 (38.0)
	DM	104 (25.0)
	CKD	16 (3.8)
	CHF	31 (7.5)
	CHD	38 (9.1)
	DM and CKD	16 (3.8)
	Dyslipidemia	39 (9.4)
	others	14 (3.4)
Duration on treatment(years)	<1	67 (16.1)
	1-5	148 (35.6)
	>5-10	144 (34.6)
	>10	57 (13.7)

Others:- Anemia, peripheral neuropathy, lung disease, liver disease, thyroid disorder, and human immunodeficiency virus infection

Prescription patterns of antihypertensive medications

The overall pattern of antihypertensive agents showed that angiotensin-converting enzyme inhibitor (ACEI) was the most frequently prescribed class of antihypertensive drugs 261(63.7%), followed by diuretics 234 (57.1%). Among the study participants, 257 (61.7%) of them had one or more concomitant medications, of which 98 (23.6%) and 75(18.0%) of them had two and three drugs concomitant medications, respectively (table 3)

Table 3: Frequency of antihypertensive medicines prescribed for hypertensive patients at JUMC, 2018.

Classes	Specific drugs	Frequency(%), N=410
**ACEIs**	Enalapril	261 (63.7)
**ARBS**	Losartan	28 (6.8)
**Diuretics**		234 (57.1)
	Hydrochlorothiazide	203 (49.5)
	Furosemide	26 (6.4)
	Furosemide +spironolactone	5 (1.2)
**CCBs**		144 (35.1)
	Amlodipine	131 (31.9)
	Nifedipine	13 (3.2)
**BBs**		105 (25.6)
	Atenolol	45 (10.9)
	Metoprolol	47 (11.5)
	Propranolol	13 (3.2)
**Co-medications**	Total number of patients with co-medication	257 (61.7)
	One drug	61 (14.7)
	Two drugs	98 (23.6)
	Three drugs	75 (18.0)
	≥ Four drugs	23 (5.5)

ACEIs: Angiotensin-converting enzyme inhibitors; ARBs: angiotensin receptor blockers; BBs: βblockers; CCBs: calcium channel blockers

Of the total participants, 135 (32.5%) were on monotherapy and 275 (66.1%) were on combination therapy. Enalapril was the most frequently prescribed monotherapy 61(14.7%) followed by hydrochlorothiazide 40(9.6%). Among patients who were on combination therapy, two-drug regimens were prescribed in 46.4% of the hypertensive patients. ACEI +diuretics, 75

(18.0%) was a commonly used two-drug combination therapy. Three drugs combination accounted for 17(17.1%) of all prescriptions, of which 35 (8.4%) were on BB+ diuretics+ ACEI followed by CCB+ACEI +diuretic 22(5.3%). Four drugs regimen was prescribed in 11 hypertensive patients, ACEI +diuretics +CCB+BB 7(1.7%) was most frequent prescribed (Table 4).

Table 4: Regimens of antihypertensive therapy among hypertensive patients at JUMC, 2018

Regimens	Specific regimens	Frequency (%)
Non-pharmacologic		6 (1.4)
Monotherapy		135(32.5)
	Enalapril	61 (14.7)
	Amlodipine	26 (6.2)
	Nifedipine	4 (1.0)
	Hydrochlorothiazide	40 (9.6)
	Losartan	4 (1.0)
Dual therapy		193 (46.4)
	ACEI + Diuretic	75 (18.0)
	CCB + BB	6 (1.4)
	Diuretic + BB	16 (3.8)
	ACEI + CCB	30 (7.2)
	ACEI + BB	24 (5.8)
	Diuretic +ARB	4 (1.0)
	CCB + ARB	8 (1.9)
	CCB+ Diuretics	26 (6.3)
	ARB+BB	4 (1.0)
Triple therapy		71 (17.1)
	BB + Diuretic + ACEI	35 (8.4)
	CCB + BB + Diuretic	7 (1.7)
	CCB +ACEI + Diuretic	22 (5.3)
	CCB + BB + ACEI	1 (0.2)
	CCB + BB +ARB	3 (0.7)
	Others*	3 (0.7)
Quadruple		11 (2.6)
	ACEI + Diuretics + CCB + BB	7 (1.7)
	ARB + BB + CCB + diuretic	1 (0.2)
	Others**	3 (0.7)

others*: Enalapril +Furosemide +Spironolactone, Amlodipine +Furosemide + Hydrochlorothiazide; others**: Atenolol+ Enalapril+ Furosemide+ Spironolactone, Amlodipine+ Enalapril+ Furosemide+ Spironolactone

Clinicians' adherence to hypertension treatment guidelines

Twenty-five full-time physicians treat hypertensive patients during the study period. There were more males (21) than females (4). The mean age of prescribers was 29.22 ± 4.51 with range 24-39 years and the majority (17) of them were below 30 years of age. Six of them were general practitioners (GPs), 4 residency year 2 (R2), 8 of them are residency year 3(R3) and 7 of them were internists. Nearly half of clinicians had experience 1-5 years and most of them treating more than 20 hypertensive patients per day. Most prescribers perceived that a JNC-8 hypertension treatment guideline was evidence-based and helpful in the management of patients. However, clinicians' overall adherence to JNC-8 guidelines was low, with only 184 (44.2%) of prescribers following them. From a total of 232 patients not treated based on the JNC-8 guideline, 102(44.0%) patients were not received first-line drugs based on compelling indication and race. In 64(27.6%) patients, combination treatment was not adjusted based on their current BP level (Table 5).

Table 5: Common problems observed from prescribed antihypertensive medications to hypertensive patients at JUMC concerning the recommendation of JNC-8 guideline, N=232.

**Compliance issue**	**Frequency**	**Percent**
Patients did not receive first-line drugs based on compelling indication and race recommendation	102	44.0
Combination treatment was not adjusted based on their current blood pressure level	64	27.6
Patients received an inappropriate dose of medications	50	21.6
Patients were not on first-line drugs and the right dose of medication	16	6.9

Patients' Adherence to Hypertension Treatment

A total of 410 study participants received one or more antihypertensive medications. From patients' response to the eight-item Morisky medication adherence Scale, Overall, the prevalence of antihypertensive medication adherence 191 (46.6%)

Determinants of medication non-adherent among hypertensive patients at JUMC, 2018

In bivariate logistic regression analysis; age, sex, marital status, residency, occupation, monthly income, living status, salt use, khat chewing, physical activity status, concomitant medications, comorbid condition, and duration of treatment were variables with P-value less than 0.25 hence, included in the multivariate logistic regression. However, current occupation, physical activity status, and presence of DM comorbidity were significantly associated with medication non-adherence. Accordingly, merchants were 2.46 times (AOR=2.46, CI=1.16-5.23, P=0.020) more likely to be non-adherent than civil servants. Patients who had no regular physical activity were 1.63 times (AOR= 1.63, CI =1.04-2.55, P=0.033) more likely to be non-adherent compared to physically active patients. Hypertensive patients with DM comorbidity were 2.54 times (AOR= 2.54, CI=1.28-5.04, P=0.008) more likely to be non-adherent as compared to clients with no comorbidity (Table 6).

Table 6: Results of logistic regression analysis for factors associated with medication non-adherence among adult hypertensive patients at JUMC, 2018

**Variables**		**Medication adherence**		**COR (95%CI) N=410**	**P-value**	**AOR (95%CI)**	**P-value**
		Adherent	Non Adherent				
**Occupation**	Civil servant	40	27	1.00	1.000	1.00	1.000
	Merchant	30	58	2.86(1.48-5.53)	0.002	2.46(1.16-5.23)	0.020
	Farmer	29	43	2.20(1.12-4.33)	0.023	1.41(0.52-3.78)	0.501
	House wife	24	22	1.36(0.64-2.89)	0.428	1.12(0.43-2.91)	0.812

	Retired	12	24	2.66(1.27-6.92)	0.012	2.04(0.66-6.26)	0.215
	Jobless	38	32	1.25(0.63-2.46)	0.522	0.71(0.26-1.91)	0.500
	Others	18	13	1.07(0.45-2.54)	0.878	0.73(0.26-2.08)	0.560
**Physical activity**	Inactive	89	130	1.67(1.13-2.48)	0.010	1.63(1.04-2.55)	0.033
	Active	102	89	1.00	1.000	1.00	1.000
**Comorbidities**	HTN alone	84	72	1.00	1.000	1.00	1.000
	DM	37	64	2.02(1.21-3.37)	0.007	2.54(1.28-5.04)	0.008
	CKD	11	5	0.53(0.18-1.59)	0.260	0.51(0.15-1.71)	0.272
	CHF	14	17	1.42(0.65-3.01)	0.378	1.14(0.48-2.71)	0.763
	CHD	13	25	2.24(1.10-4.70)	0.032	2.13(0.88-5.14)	0.093
	DM+CKD	11	5	0.53(0.18-1.60)	0.260	0.51(0.15-1.79)	0.294
	Dyslipidemia	17	21	1.44(0.71-2.94)	0.315	1.14(0.51-2.53)	0.751
	Others**	4	10	2.92(0.88-9.69)	0.081	2.81(0.77-10.22)	0.116

COR= crude odds ratio; AOR= Adjusted odds ratio: others**: Drivers, daily laborer, non-governmental organization and private work; others**: Anemia, peripheral neuropathy, lung disease, liver disease, thyroid disorder, and human immunodeficiency virus infection

Discussion

Medication adherence is the main predictor of treatment success and an important step in lowering BP. The present study showed that the overall rate of patients' medication adherence was 191 (46.6%). The finding was almost similar to the WHO report in 2011 in developing countries(50%) [38]. It was also comparable to studies done in Egypt (46.12%) and Saudi Arabia (46%) [39, 40]. It was, however, lower than studies reported from Taiwan (53%), Sweden (63.1%), and China (53.4%) [41, 42, 43]. The adherence discrepancy between the studies could be due to variations in the studied populations, better health care, and access to health in the developed countries. Moreover, differences in study results could be due to differences in measurement methodologies employed to assess drug adherence. For example, in Taiwan medication adherence was measured using the medication possession ratio (percentage of time that the patient had medication available to them during the follow-up period) whereas, in Sweden adherence was measured by using the Proportion of Days Covered (PDC) method.

This finding was also lower than the studies conducted in Gondar and Jimma university (64.6% and 61.8%, respectively) [44,45]. The discrepancy might be due to the varaition in the inclusion criteria and adherence measurement scale. Participants in the Jimma University study were

hypertension patients who had been followed for at least a year, and the sample size (280 patients) was lower than in this study. The Morisky 4-item Medication Adherence Scale was used to assess adherence at Gondar University. The medication adherence level found in this study, on the other hand, was higher than the finding from Ghana and Nigeria (30.3%) and Iran (24%) [46,47]. This disparity could be explained by the study done in Ghana and Nigeria included hypertensive patients who had depression which may have contributed to the low medication adherence. Similarly, in Iran, participants were selected from the rural area only which might have contributed to medication non-adherence.

In this study, a significant association was observed between patients' current occupation and medication nonadherence. Merchant hypertensive patients were 2.46 times more likely to be nonadherent than civil servants. This finding was in line with studies done in Hong Kong China and Black Loin Specialized Hospital, Ethiopia [48,49]. This might be because they forget to take drugs with themselves when they travel or leave home. Besides, they may be too busy to come to the health facility for their pills, and they may have difficulties in remembering to take all of their medicine on time. Physical inactivity was another important factor influencing patients' adherence in this study. Patients who had no regular physical activity were 1.63 times more likely to be non-adherent than physically active patients. This was consistent with a finding from Iran [47]. The exact mechanism was unclear, but it might be that physically inactive hypertensive patients will have uncontrolled BP (57.2 % in this study), prompting clinicians to prescribe a more complex treatment regimen. Furthermore, patients with uncontrolled BP might be hopeless to take their medications and adhere to their treatment plan.

In addition, the presence of DM as comorbidity was significantly associated with poor antihypertensive medication adherence. Hypertensive patients with DM comorbidity were 2.54 times more likely to be nonadherent than with no co-morbidities. This finding was similar to studies conducted in South Korea, Hong Kong China, Saudi Arabia, and Gondar, Ethiopia [10,48, 40, 44]. This could be explained by patients with co-morbidities who may have suffered from serious complications and had complex treatment regimens making it difficult for them to be adherent to their medications. Furthermore, 27.4% of participants in this study had no formal education, making it difficult to adhere to treatment plans and take complex treatment on time. This indicated that close supervision, short time appointments and fixed-dose combinations are important to ehance medication adherence for this group of patients.

This study found that 44.2% of clinicians' were adherent to hypertension treatment guidelines. The rate of adherence was slightly higher than the study conducted in Zewditu Memorial Hospital, Ethiopia (37.4%) [26]. This difference might be due to the small sample size, and most of the study participants (65.2%) were without co-morbid conditions in Zewditu Memorial Hospital. However, it was lower than studies conducted in South Indian (65%), Malaysia (85.30%), Island (70.4%), and South Africa (51.9%) [21, 23, 37, 29]. This disparity might be due to racial differences, as well as lack of laboratory and imaging facilities in our setting for further screening of target organ damage. In addition, it might be explained by the difference in prescribers profiles since the majority of doctors treating hypertensive patients in this study were general practitioners and residents. Six of them were general practitioners (GP), 4 residency year 2 (R2), 8 of them are residency year 3(R3) from the total of 25 doctors. The common challenges to adherence to JNC-8 hypertension treatment guidelines reported by prescribers were the cost of medication, lack of medication availability, and the presence of comorbidity. Ethiopias' Ministry of Health and other concerned bodies must continue to prioritize health care accessibility and affordability. The strong commitment of the Ethiopian ministry of health and the institution for health care improvement outcomes including fidelity, accesseblity of well-trained health care professionals, laboratory and imaging facilities for further screening for target organ damage, and program effectiveness are important to enhance clinicians guideline adherence in a health care setting.

According to JNC-8 guideline, CCBs and thiazide diuretics are the recommended first-line medicines for the management of hypertension for Africa origin. However, ACEIs were prescribed first, followed by diuretics and CCBs in this study (63.7%, 57.1%, 35.1% respectively). The most commonly prescribed class of drug among hypertensive patients with DM, CHF, or CHD was ACEI (66.3 percent, 71.0 percent, and 65.8 percent, respectively), whereas diuretics were the most commonly prescribed drug in CKD. This might be because prescribers (clinicians) were not adherent to standard treatment guidelines (JNC-8). ACEIs are preferable for CKD patients due to renal protective effects. The overall use of ACEIs was comparable to studies conducted in Serbia (60.57%) and Mexico City (63.8%) [30,31]. Thiazide diuretics were the most frequently prescribed medications in studies conducted in the Eastern Central Region of Portugal and rural tertiary hospitals in Nigeria showed that (67%, and 84.9%), respectively [29, 16]. This disparity could be attributed to studies in Portugal and Nigeria that used JNC-7 as a reference guideline, which is conservative in its use of thiazide-type diuretics as first-line therapy for most patients unless there is a compelling indication.

The result of this study showed that 135 (32.5%) patients were on mono-therapy regardless of the presence or absence of comorbidities, which was lower than the studies reported from Mexico (72.1%), Canada (56.3%), and Turkey (75.7%) [31-33]. The difference might be due to better health care, availability of medications, and close monitoring in these countries which help to achieve target BP with a single medication. Of the total of patients who were on monotherapy, ACEIs, 61 (14.7%) were the most prescribed drug classes. The result was closely similar to a study conducted in Kenya (20.2%) [34]. However, it was lower than the study conducted in Turkey (30.1%) [33]. The variation might be due to using different standard reference guidelines in which European guidelines recommend any class of drug as initial therapy. On contrary, the study conducted in Gondar hospital showed that thiazide diuretics were the most commonly prescribed mono-therapy (60.24%) [36]. this discrepancy might be because of the difference in level guideline adherence (66.8% prescription based on JNC-8 guidelines) that recommended the use of diuretics for both mono and combination therapy. In addition, variation may be different in the prevalence of CKD co-morbidity, as only 3.8% of participants had CKD comorbidity in this study.

On the other hand, the majority of the patients, 275(66.1%) were on combination therapy. The finding was consistent with the study conducted in Kenya (60%) [34]. It was also similar to a study conducted at Zewditu Memorial Hospital Ethiopia (70.8%) [26]. However, the finding of this study was higher than the study conducted in Malaysia (56.7%) [35]. The higher prescription rate of combination therapy in this study might be due to the low rate of BP control 42.8% in this study as compared to 84.6% in Malaysia.

Two drugs regimen accounted for 46.4 % of the combination therapy, of this 75(18%) were ACEIS + diuretics. The finding was in line with a study conducted in Kenya (14.5%) [34]. A study conducted in India using the JNC-8 guideline showed that the most frequently prescribed two-drug combination was ARB +diuretics [25]. This variation might be due to the cost and easy accessibility of ARB in India. On the other hand, in the study conducted in Nigeria, CCB + diuretic was the most frequently used two-drug combination (36.6%) [16]. This variation might be suggesting of doctor's preference to use the CCBs combination as initial therapy. Besides, in Nigeria majority of the patients were elderly (mean age was 61.5±15.1 years) hence, CCBs are preferable for older patients because isolated SBP is more prevalent due to vessel stiffness(50).

Limitations of the Study

This study has some limitations. The study was conducted in one facility, therefore; the findings may not be generalized to reflect all health care setting in Ethiopia. Only prescription and co-morbidity data were used to examine compliance to treatment guidelines, which may not always be reliable. Finally, this study was unable to identify factors affecting clinicians' adherence to standard treatment guidelines but will be an interesting area for future research.

Conclusion

The rate of medication adherence was poor among hypertensive patients. Being merchant, physical inactivity, and the presence of DM co-morbidity were factors associated with poor medication adherence. Prescribing patterns of antihypertensive drugs were inconsistent with JNC-8 guidelines. Angiotensin-converting enzyme inhibitors were the most frequently prescribed class of anti-hypertensive drugs. The majority of participants were on combination therapy. Effective education should be given to patients to improve medication adherence. Prescribers should be trained on treatment guidelines regularly to keep them up-to-date with current trends of hypertension treatment and for better treatment outcomes.

Abbreviation

BP: blood pressure, SBP: systolic blood pressure, DBP: Diastolic blood pressure, CHD: coronary heart disease, DM: diabetes mellitus, CKD: chronic kidney disease, CVD: cardiovascular diseases, IBM: international business machine, JNC-8: eight joint national committees, BB: beta-blocker, CCB: calcium channel blocker, ARB: angiotensin receptor blocker, ACEI: angiotensin-converting enzyme inhibitor, TD: thiazide diuretic, MMAS-8: Morisky medication adherence scale-8, AOR: adjusted odds ratio, COR: crude odds ratio.

Declarations

Conflict of interest: The authors declared that they have no conflict of interest.

Acknowledgments: We thank the participants of the study.

Availability of data and materials: **All relevant data are within the manuscript and its supporting information** files.

Authors' contributions: Bekalu Kebede: concept and design, data acquisition, interpretation of data, manuscript preparation; Legese Chelkeba: concept and design, interpretation of data, manuscript preparation, manuscript revision, and manuscript review. Bekalu Dessie: interpretation of data, manuscript preparation, manuscript revision, and manuscript review. All authors read and approved the final manuscript

References

1. Catherine P. Benziger GA. Roth AE. The Global Burden of Disease Study and the Preventable Burden of NCD. Journal of global heart.2016;11(4):393-397
2. Bromfield S, Muntner P. high blood pressure: the leading global burden of disease risk factor and the need for worldwide prevention programs. Curr Hypertens Rep. 2013;15(3):134-6.
3. Belue R, Okoror TA, Iwelunmor J, Taylor KD, Degboe AN, Agyemang C, et al. Globalization and Health An overview of cardiovascular risk factor burden in sub-Saharan African countries: a socio-cultural perspective. 2009; 12:1–12
4. Kibret KT, Mesfin YM. Prevalence of hypertension in Ethiopia: a systematic meta-analysis. Public Health Rev. 2015; 36:14.

5. Molla M. Systematic Reviews of Prevalence and Associated Factors of Hypertension in Ethiopia: Finding the Evidence. *Science Journal of Public Health*. 2015,3(4), 514-519.
6. Sarki AM, Nduka CU, Stranges S, Kandala NB, Uthman OA. Prevalence of Hypertension in Low- and Middle-Income Countries: A Systematic Review and Meta-Analysis. Medicine (Baltimore). 2015;94(50): e1959.
7. Ibrahim MM, Damasceno A. Hypertension in developing countries. Lancet. 2012;380(9841):611-9.
8. Grassi G, Cifkova R, Laurent S, Narkiewicz K, Redon J, Farsang C, et al. Blood pressure control and cardiovascular risk profile in hypertensive patients from central and eastern European countries. Eur Heart J. 2011; 32, 218–225
9. Schmieder RE, Mozaffarian D, Véronique L, Emelia J, Jarett D, Michael J et al. End Organ Damage in Hypertension. Circulation;2014; 129(3):1-19.
10. Shin S, Song H, Sang-Kwon Oh, Eob KC, Kim H, Jang S. Effect of antihypertensive medication adherence on hospitalization for cardiovascular disease and mortality in hypertensive patients. Hypertension Research.2013; 36(11):1000-1005.
11. Venkataraman R, Rashid M, Shashikantha B, Soumya A, Vijayan G, Manuel GG, Islam S. Prescribing pattern of antihypertensive medication and adherence to Joint National Commission-8 guidelines in a rural tertiary care Indian teaching hospital. Journal of basic and clinical physiology and pharmacology. 2020 Jan 1;31(1).
12. Ettehad D, Emdin CA, Kiran A, Anderson SG, Callender T, Emberson J, et al. Blood pressure lowering for prevention of cardiovascular disease and death: a systematic review and meta-analysis. Lancet, 2016; 387(10022), 957–967.
13. Jellinger PS, Handelsman Y, Rosenblit PD, Bloomgarden ZT, Fonseca VA, Garber AJ, Grunberger G, etal. American association of clinical endocrinologists and American college of endocrinology guidelines for management of dyslipidemia and prevention of cardiovascular disease. Endocr pract. 2017 apr;23(suppl 2):1-87
14. James PA, Oparil S, Carter BL, Cushman WC, Dennison-Himmelfarb C, Handler J etal. 2014 Evidence-Based Guideline for the Management of high blood pressure in adults reports from the panel members appointed to the eighth Joint National Committee (JNC 8). JAMA. 2014;311(5):507-20
15. Gebrihet TA, Mesgna KH, Gebregiorgis YS, Kahsay AB, Weldehaweria NB, Weldu G. Awareness, treatment, and control of hypertension is low among adults in Aksum town, northern Ethiopia: A sequential quantitative-qualitative study. PLoS One. 2017;12(5):e0176904.
16. Busari OA, Oluyombo R, Fasae AJ, Gabriel EO, Ayodele LM, Agboola SM. Prescribing pattern and utilization of antihypertensive drugs and blood pressure control in adult patients with systemic hypertension in a rural tertiary hospital in Nigeria. Am J Inter Med. 2014;2(6):144-9

17. Parati G, Omboni S, Compare A, Grossi E, Callus E, Venco A, et al. Blood pressure control and treatment adherence in hypertensive patients with metabolic syndrome: protocol of a randomized controlled study based on home blood pressure telemonitoring vs. conventional management and assessment of psychological determinants of adherence(TELEBPMET Study). trials; 2013;1–11.
18. Asgedom SW, Gudina EK, Desse TA. Assessment of Blood Pressure Control among Hypertensive Patients in Southwest Ethiopia. PLoS One. 2016;11(11):e0166432.
19. Papatya Karakurt, Mağfiret Kaşikçi, Factors affecting medication adherence in patients with hypertension, Journal of Vascular Nursing. 201230(4), 118-126,
20. Ahmed A, Saqlain M, Tanveer M, Nawaz MS, Rehman K, Safdar A, Tayyeb M, Ali Z, Shinwari M, Baig RU. Prescribing Patterns of Antihypertensive Drugs in Patients Attending Tertiary Care Hospitals in Pakistan. SN Comprehensive Clinical Medicine. 2021 Jan;3(1):176-82.
21. Datta S. Utilization Study of Antihypertensives in a South Indian Tertiary Care Teaching Hospital and Adherence to Standard Treatment Guidelines. J Basic Clin Pharm. 2016;8(1):33-37.
22. Mutua EM, Gitonga MM, Mbuthia B, Muiruri N, Cheptum JJ, Maingi T. Level of blood pressure control among hypertensive patients on follow-up in a regional referral hospital in Central Kenya. Pan Afr Med J. 2014;18:278.
23. Abdulameer SA, Sahib MN, Aziz NA, Hassan Y, Alrazzaq HA, Ismail O. Physician adherence to hypertension treatment guidelines and drug acquisition costs of antihypertensive drugs at the cardiac clinic: a pilot study. Patient Prefer Adherence. 2012;6:101-8.
24. An J, Luong T, Qian L, Wei R, Liu R, Muntner P, Brettler J, Jaffe MG, Moran AE, Reynolds K. Treatment Patterns and Blood Pressure Control With Initiation of Combination Versus Monotherapy Antihypertensive Regimens. Hypertension. 2021 Jan;77(1):103-13..
25. Romday R, Gupta A, Bhambani P. An assessment of antihypertensive drug prescription patterns and adherence to joint national committee-8 hypertension treatment guidelines among hypertensive patients attending a tertiary care teaching hospital. International Journal of Research in Medical Sciences. 2016;4(12).5125-33.
26. Yazie D, Shibeshi W, Woldu M, Berha A. Assessment of Blood Pressure Control among Hypertensive Patients in Zewditu Memorial Hospital, Addis Ababa, Ethiopia: A Cross-Sectional Study. Journal of Bioanalysis & Biomedicine. 2018;10.1-53
27. Beger C, Unger T, Haller H, Limbourg FP. Antihypertensive prescription patterns and cardiovascular risk in patients with newly diagnosed hypertension-an analysis of statutory health insurance data in Germany. Blood Pressure. 2020 Nov 1;29(6):357-61.
28. Morgado MP, Rolo SA, Pereira L, Castelo-Branco M. Blood pressure control and antihypertensive pharmacotherapy patterns in a hypertensive population of Eastern Central Region of Portugal. BMC Health Serv Res. 2010;10:349.

29. Adedeji AR, Tumbo J, Govender I. Adherence of doctors to a clinical guideline for hypertension in Bojanala district, North-West Province, South Africa. Afr J Prim Health Care Fam Med. 2015;7(1):776.
30. Tomas A, Tomić Z, Milijasević B, Ban M, Horvat O, Vukmirović S et al. Patterns of prescription antihypertensive drug utilization and adherence to treatment guidelines in the city of Novi Sad. Vojnosanit Pregl. 2016;73(6):531-7.
31. Alba-Leonel A, Carvajal A, Fierro I, Castillo-Nájera F, Campos-Ramos O, Villa-Romero A et al. Prescription patterns of antihypertensives in a community health center in Mexico City: a drug utilization study. Fundam Clin Pharmacol. 2016; 30(3):276-81.
32. Beaulieu MD, Dufresne L, LeBlanc D. Treating hypertension. Are the right drugs given to the right patients? Can Fam Physician. 1998; 44:294-8, 301-2.
33. Abaci A, Kozan O, Oguz A, Sahin M, Deger N, Senocak H et al. Prescribing pattern of antihypertensive drugs in primary care units in Turkey: results from the TURKSAHA study. Eur J Clin Pharmacol. 2007 Apr;63(4):397-402.
34. Mbui JM, Oluka MN, Guantai EM, Sinei KA, Achieng L, Baker A et al. Prescription patterns and adequacy of blood pressure control among adult hypertensive patients in Kenya; findings and implications. Expert Rev Clin Pharmacol. 2017; 10(11):1263-1271.
35. Ramli A, Miskan M, Ng K, Ambigga D, Nafiza M, Mazapuspavina M, Sajari J, Ishak R. Prescribing of Antihypertensive Agents in Public Primary Care Clinics - is it per Current Evidence? Malays Fam Physician. 2010;5(1):36-40.
36. Abegaz TM, Tefera YG, Abebe TB. Antihypertensive drug prescription patterns and their impact on the outcome of blood pressure in Ethiopia: a hospital-based cross-sectional study. Integr Pharm Res Pract. 2017; 6:29-35.
37. Theodorou M, Stafylas P, Kourlaba G, Kaitelidou D, Maniadakis N, Papademetriou V. Physicians' perceptions and adherence to guidelines for the management of hypertension: a national, multicentre, prospective study. Int J Hypertens. 2012;2012:503821.
38. Brown MT, Bussell JK. Medication adherence: WHO cares? Mayo Clin Proc. 2011; 86(4):304-14.
39. Hussein A, Awad MS, Mahmoud HE. Patient adherence to antihypertensive medications in upper Egypt: a cross-sectional study. The Egyptian Heart Journal. 2020 Dec;72:1-8.
40. Khayyat SM, Khayyat SM, Hyat Alhazmi RS, Mohamed MM, Abdul Hadi M. Predictors of Medication Adherence and Blood Pressure Control among Saudi Hypertensive Patients Attending Primary Care Clinics: A Cross-Sectional Study. PLoS One. 2017;12(1):e0171255.
41. Lee CY, Huang CC, Shih HC, Huang KH. Factors influencing antihypertensive medication compliance in Taiwan: a nationwide population-based study. Eur J Prev Cardiol. 2013; 20(6):930-7.

42. Hedna K, Hakkarainen KM, Gyllensten H, Jönsson AK, Andersson Sundell K, Petzold M, Hägg S. Adherence to Antihypertensive Therapy and Elevated Blood Pressure: Should We Consider the Use of Multiple Medications? PLoS One. 2015;10(9):e0137451.
43. Li YT, Wang HHX, Liu KQL, Lee GKY, Chan WM, Griffiths SM et al. Medication Adherence, and Blood Pressure Control Among Hypertensive Patients With Coexisting Long-Term Conditions in Primary Care Settings: A Cross-Sectional Analysis. Medicine (Baltimore). 2016; 95(20):e3572.
44. Ambaw AD, Alemie GA, W/Yohannes SM, Mengesha ZB. Adherence to antihypertensive treatment and associated factors among patients on follow-up at University of Gondar Hospital, Northwest Ethiopia. BMC Public Health. 2012;12:282.
45. Asgedom SW, Atey TM, Desse TA. Antihypertensive medication adherence and associated factors among adult hypertensive patients at Jimma University Specialized Hospital, southwest Ethiopia. BMC Research Notes. 2018;11(1):27.
46. Boima V, Ademola AD, Odusola AO, Agyekum F, Nwafor CE, Cole H, et al. Factors Associated with Medication Nonadherence among Hypertensives in Ghana and Nigeria. Int J Hypertens. 2015;2015:205716.
47. Kamran A, Sadeghieh Ahari S, Biria M, Malepour A, Heydari H. Determinants of Patient's Adherence to Hypertension Medications: Application of Health Belief Model Among Rural Patients. Ann Med Health Sci Res. 2014; 4(6):922-7.
48. Kang CD, Tsang PP, Li WT, Wang HH, Liu KQ, Griffiths SM et al. Determinants of medication adherence and blood pressure control among hypertensive patients in Hong Kong: a cross-sectional study. Int J Cardiol. 2015 Mar 1;182:250-7.
49. Hareri HA, Abebe M and Asefaw T: Assessments of adherence to Hypertension managements and its influencing factors among Hypertensive patients attending Black Lion Hospital chronic follow up a unit, Addis Ababa, Ethiopia-a cross-sectional study. *Int J Pharm Sci Res* 2013; 4(3); 1086-1095.
50. Ogundele SO, Ajibare AO, Amisu MA, Dada AO. Prescription pattern and blood pressure control among patients on antihypertensive medications attending a tertiary hospital in Lagos: A cross-sectional study. Nigerian Journal of Basic and Clinical Sciences. 2021 Jan 1;18(1):9.

Figure Click here to access/download;Figure;Figure.docx

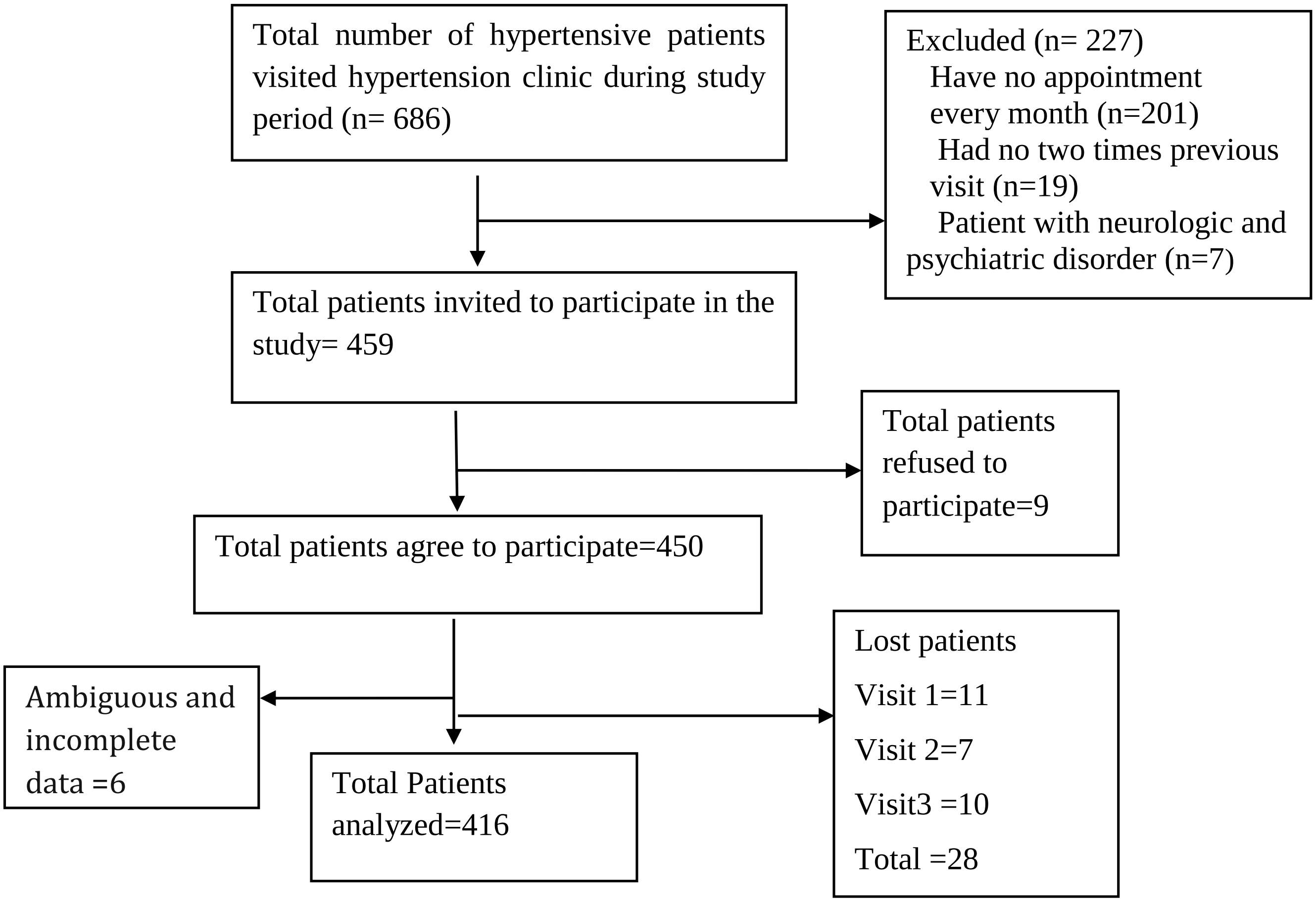

Figure1: Hypertension patients selection flow chart at Jimma University Medical Center from March 20, 2018 to June 20, 2018.

Click here to access/download
Supporting Information
Questionnaire.docx

Clinicians' Prescribing Pattern, Rate of Patients' Medication Adherence and Its Determinants among Adult Hypertensive Patients at Jimma University Medical Center: Prospective Cohort Study

Bekalu Kebede, MSc[1*], Legese Chelkeba, PhD[2], Bekalu Dessie, MSc[1]

[1] Department of Pharmacy, College of Health Sciences, Debre Markos University, Debre Markos, Ethiopia

[2]Department of Clinical Pharmacy, School of Pharmacy, College Health Sciences, Addis Ababa University, Addis Ababa, Ethiopia

***Corresponding author: Bekalu Kebede**

Clinical Pharmacist, Department of Pharmacy, College of Health Sciences, Debre Markos University, Debre Markos, Ethiopia

Phone: +251-921-286-140

Email: bekalukebede19@gmail.com

Abstract

Background: Many studies conducted in the past focused on patients' sociodemographic factors and medical profiles to identify the determinants of suboptimal blood pressure control. However, prescribing patterns and clinicians' adherence to guidelines are also important factors affecting the rate of blood pressure control. Therefore, this study aimed to determine clinicians' prescribing patterns, patients' medication adherence, and its determinants among hypertensive patients at Jimma University Medical Center.

Methods: A general prospective cohort study was conducted among hypertensive patients who had regular follow-up at Jimma university ambulatory cardiac clinic from March 20, 2018, to June 20, 2018. Patients' specific data was collected with a face-to-face interview and from their medical charts. Clinicians' related data were collected through a self-administered questionnaire. Data were analyzed using SPSS version 21.0. Bivariate and multivariable logistic regression analyses were done to identify key independent variables influencing patients' adherence. P-Values of less than 0.05 were considered statically significant.

Results: From the total of 416 patients, 237(57.0%) of them were males with a mean age of 56.50 ± 11.96 years. Angiotensin-converting enzyme inhibitors were the most frequently prescribed class of antihypertensives, accounting for 261(63.7%) prescriptions. Combination therapy was used by the majority of patients, with 275 (66.1%) patients receiving two or more antihypertensive drugs. Patients' medication adherence was 46.6%, while clinicians' guideline adherence was 44.2%. Patients with merchant occupation (P=0.020), physical inactivity (P=0.033), and diabetes mellitus co-morbidity (P=0.008) were significantly associated with a higher rate of medication non-adherence.

Conclusion: The rate of medication adherence was poor among hypertensive patients. Physicians were not-adherent to standard treatment guideline. The most commonly prescribed class of drugs were angiotensin-converting enzyme inhibitors. Effective education should be given to patients to improve medication adherence. Prescribers should be trained on treatment guidelines regularly to keep them up-to-date with current trends of hypertension treatment and for better treatment outcomes.

Keywords: Prescribing Pattern, Blood Pressure Control, Guidelines, Adherence

Introduction

Hypertension is a significant public health challenge in the world because of its high prevalence and concomitant risks of cardiovascular and kidney diseases [1]. According to the World Health Organization (WHO), high blood pressure (BP) is a major public health problem that kills one in every eight people and is the world's third-leading silent killer [2].

Almost three-quarters of hypertensive people (639 million people) live in countries with limited health resources [1, 2]. In Sub- Saharan Africa, it is a major independent risk factor for heart failure, stroke, and kidney failure. These complications arise as a result of a low rate of hypertension diagnosis, poor BP control, high morbidity and mortality, and low resources in health care settings [3]. A systematic review and meta-analysis study conducted in Ethiopia in 2015 estimated the prevalence of hypertension to be 19.6 % [4]. Another systematic review and meta-analysis conducted in Ethiopia in 2015 found that the prevalence of hypertension ranged from 20% and 30% [5].

Despite the fact that hypertension is a preventable and modifiable risk factor for cardiovascular diseases (CVD), the prevention and control of hypertension have not received attention in many developing countries [6,7]. It accounts for 40.6% of deaths due to all coronary heart disease and 38.5% of deaths due to stroke [8]. It is also the second-leading cause of end-stage renal disease (ESRD) after diabetes mellitus (DM [9].

Guideline adherence is defined as a condition in which the prescribed treatment obeys treatments recommended in the identified practice guidelines [10-12]. Hypertension guidelines are evidence-based and are usually dictated by randomized controlled trial data and observational studies. Published guidelines aid in clinical decision making, decrease practice variations, guide correctness, and measure the quality of health care [13-14]. The Eighth Joint National Committee (JNC-8) on detection, evaluation, and treatment of BP is most the commonly used guideline and it is considered as a "gold standard" consensus guideline for the management of hypertension [15]. This guideline was used as a reference standard for this study because awareness and accessibility of guidelines were initial criteria to evaluate the status of clinicians' adherence to hypertension treatment guidelines. Besides, Ethiopia does not have a hypertension treatment guideline. Therefore, the JNC-8 guideline is widely used by both clinicians and clinical pharmacists in Ethiopia [16]. Clinical practice guidelines do not

consistently change clinicians' behavior, control of high blood pressure remains suboptimal, so creating the need to evaluate adherence to guidelines and its impact on BP control [10].

Many studies conducted in the past to explore the causes of suboptimal BP control focused on patient factors such as socio-demographic, medical profile, and patient's treatment compliance [17-20]. However, prescribing patterns and clinicians' adherence to guidelines are also important factors affecting hypertension treatment outcomes [21-24].

Several studies were conducted worldwide using either prescription or drug dispensing data to evaluate prescription patterns and clinicians' adherence to hypertension treatment guidelines [25-27]. But few data are available in Ethiopia. Also, among previous studies, there were conflicting results between prescribing patterns and patients' medication adherence. A study conducted in Kenya concluded that combination therapies were associated with poor patient adherence [22]. In contrast, in a study conducted in Nigeria combination therapies were associated with good blood pressure controlled than mono-therapy [16]. A study conducted at Jimma University, Ethiopia showed that the number of antihypertensive medications prescribed was not associated with blood pressure control status [18]. Besides, most previous studies used a similar study design(cross-sectional). As a result, a general prospective cohort study was conducted among ambulatory hypertension patients to determine prescribing patterns of antihypertensive drugs, patients' medication adherence, and its determinants.

Methods

Study period and setting

This study was conducted over three months among hypertensive patients who were regularly followed up every month at Jimma university ambulatory cardiac clinic from March 20, 2018, to June 20, 2018. Jimma University is the only teaching and specialized hospital in the Jimma zone. It serves over 15 million people living in Jimma city and its surrounding areas. It is located in Jimma town, Oromia Regional State, Ethiopia, 352 km southwest of Addis Ababa, the capital city of Ethiopia (18).

Study design: a general prospective cohort follow-up study was employed

Study population; All hypertensive patients with age ≥ 18 years, with a documented diagnosis, had at least two times medical appointments before the study period, having a medical appointment every month and complete medical records were included in the study. We

excluded pregnant women, patients with confirmed neurologic and psychiatric disorders with their respective specialists since it was challenging to assess medication adherence statuses in acutely ill patients with similar tools like that of other study participants.

Sample size and sampling technique: The sample size for this study was calculated using Cochran single proportion formula. A previous study conducted in Ethiopia reported that the rate of patients' medication adherence was 50 % [18]. Based on this formula minimum 384 sample size was calculated using a standard normal distribution (Z=1.96) with a confidence interval of 95% and a margin of error of 0.05. After adjusting the 10% non-respondent rate, 416 hypertensive patients were included in this study. All clinicians who treated hypertensive patients during the study period were included and a random sampling technique was used to select patients for inclusion in the study.

Data collection process and data collection tool

Questionnaire and data abstraction format was prepared by reviewing different literatures. To maintain the validity of the data collection tool, a structured questionnaire was developed and translated to local languages (Amharic and Afan Oromo) by native speakers of the respective languages, and then back-translated to the English version. A pre-test of the data collection format was performed on 5% of the sample size. Face-to-face interviews were use to collect primary data from patients, and a self-administered questionnaire was used to collect data from all clinicians who treat hypertensive patients during the study period. During each visit, secondary data such as prescribed drugs, blood pressure measurements, and co-morbid illness were collected from patients' charts. Initial and subsequent BP is typically measured at Jimma University Medical Center, using the cuff sphygmomanometer and digital sphygmomanometer on the left arm, at the level of the heart, in a sitting position, and at rest for at least 5 minutes. In this study, hypertensive patients who met the inclusion criteria were selected at the first month of the data collection period and BP measurements at this first visit were considered as a baseline. Then only those patients selected at the first month were consequently followed every month for the next three months. Morisky Medication Adherence Scale-8 (MMAS-8) was used to assess the patients' medication adherence. The MMAS-8 total score was calculated by summing the values from all 8 items, with reverse coding when necessary. A score of <2 (out of a full range of 8) was considered as good medication adherence, otherwise considered as poor adherence [19].

Pharmacological Trends Guideline Adherence Evaluation Method was used to measure clinicians' adherence to hypertension treatment guidelines [25]. A recommendation of the JNC-8 on the detection, evaluation, and treatment of high BP was used as a reference guideline to evaluate prescription patterns and clinicians' adherence to the guidelines.

Data processing and analysis: The data were entered using Epidata version 3.1 and exported to the Statistical Package for Social Science (SPSS) version 21.0. Continuous variables were presented as means (standard deviation). Categorical variables were presented as frequency and percentages. Antihypertensive medicines were categorized according to their therapeutic classes. Switch from one drug to another drug during each visit, the last regimen was included in the analysis unless changing was done at the last visit.

Guideline adherence was expressed as % = (Total number hypertensive patients treat based on JNC-8 guideline divide by the total number of participants) × 100 [23]. Based on this calculation, >65% conceder as complete adherence, 50 – 64.9% medium adherence, and <49.9% will be classified as low adherence [29]. Finally, BP was calculated by taking an average of three measurements and categorized controlled and uncontrolled based on guidelines. Prior to regression alalysis, a multi-collinearity test was done , and adequacy of cell distribution was checked using the chi-square test. Binary logistic regression was performed to determine the effect of each variable on patients' adherence and variables with a P-value less than 0.25 in the bivariate analysis were then included in a multivariate logistic regression analysis to identify key independent variables influencing patient's medication adherence. In multivariate analysis, variables with P- a value of < 0.05 were considered statistically significant.

Ethics approval and consent to participates: This study was approved by the Ethical Review Board of School of Pharmacy, Institute of Health Science, Jimma University (Ref.No IHRPGD/203/18). After a detailed explanation of the objective of the study, procedures of selection, and assurance of confidentiality, each participant were asked orally whether they are voluntary or not to participate in this study. An independent data collection supervisor acted as a witness for voluntary informed decision-making of participants to take part in the study. Written informed consent was waived since the study did not involve any procedure and presented no damage to patients as approved by the ethical review board committee of the Board of School of Pharmacy, Institute of Health Science Jimma University. Their names were not registered to minimize social

desirability bias and enhance anonymity. They were not forced to participate or receive any monetary incentive and it was solely voluntary based.

Results

Baseline characteristics of study participants

A total of 686 hypertensive patients visited the hypertension clinic during the study period. Four hundred fifty-nine patients fulfilled inclusion criteria, of these, 416 participants were included in the final analyses (Fig 1). More than half (57.0%) of the participants were males with a mean age of 56.50 ± 11.96 years (Table 1).

Table 1: Socio-demographic characteristics of hypertensive patients at JUMC, 2018

Variables	Characteristics	Frequency (%)
**Age (year)**	Mean ± SD	56.50±11.96
	<60	220(52.9)
	≥60	196(47.1)
**Sex**	Male	237 (57.0)
	Female	179 (43.0)
**BMI**	Mean ± SD	24.24±2.32
	18.5-24.9	314 (75.5)
	25-29.9	89 (21.4)
	≥30	13 (3.1)
**Marital status**	Single	12 (2.9)
	Married	284 (68.3)
	Divorced	55 (13.2)
	Widowed	65 (15.6)
**Residency**	Urban	274 (65.9)
	Rural	142 (34.1)
**Education level**	No formal education	114 (27.4)
	Primary education (1-8 grade)	110 (26.4)
	Secondary education (9-12 grade)	100 (24.0)
	Tertiary education (diploma & above)	92 (22.1)
**Current occupation**	Civil servant	69 (16.6)
	Merchant	89 (21.4)
	Farmer	74 (17.8)
	Housewife	47 (11.3)
	Retired	36 (8.7)
	Jobless	70 (16.8)
	Other	31 (7.5)
**Monthly income (ETB)**	<1000	58 (13.9)
	1001-2000	68 (16.3)
	2001-3000	74 (17.8)
	>3000	109 (26.2)

	Without unknown monthly income	107 (25.7)
**Living status**	Solo	59 (14.2)
	Live with family	328 (78.8)
	Others*	29 (7.0)

Others: Drivers, daily laborer, Non-governmental organization and private work; others*: Live with friends, prison; ETB: Ethiopian Birr

Two hundred sixty-four (63.5%) of participants have been taking salt with food. More than half (53.4%) of the participants were physically inactive and 138 (33.2%) patients were khat chewers. Among co-morbid conditions, 104(25.0 %) of participants had diabetes mellitus (DM), 38(9.1%) and 39(9.4%) had coronary heart disease (CHD) and dyslipidemia, respectively (Table 2).

Table 2: Lifestyle factors and clinical characteristics of hypertensive patients at JUMC, 2018

Factors	**Categories**	**Frequency (%)**
Add salt to food	Yes	264 (63.5)
	No	152 (36.5)
Alcohol use	Yes	103 (24.8)
	No	313 (75.2)
Chew khat	Yes	138 (33.2)
	No	278 (66.8)
Cigarette smoking	Never smoked	303 (72.8)
	Ex-smoker	93 (22.4)
	Current smoker	20 (4.8)
Physical activity	Physically active	194 (46.6)
	Physically inactive	222 (53.4)
Drink coffee	Yes	238 (57.2)
	No	178 (42.8)
Use traditional medicine	Yes	16 (3.8)
	No	400 (96.2)
Co morbidity	Hypertension alone	158 (38.0)
	DM	104 (25.0)
	CKD	16 (3.8)
	CHF	31 (7.5)
	CHD	38 (9.1)
	DM and CKD	16 (3.8)
	Dyslipidemia	39 (9.4)
	others	14 (3.4)
Duration on treatment(years)	<1	67 (16.1)
	1-5	148 (35.6)
	>5-10	144 (34.6)
	>10	57 (13.7)

Others:- Anemia, peripheral neuropathy, lung disease, liver disease, thyroid disorder, and human immunodeficiency virus infection

Prescription patterns of antihypertensive medications

The overall pattern of antihypertensive agents showed that angiotensin-converting enzyme inhibitor (ACEI) was the most frequently prescribed class of antihypertensive drugs 261(63.7%), followed by diuretics 234 (57.1%). Among the study participants, 257 (61.7%) of them had one or more concomitant medications, of which 98 (23.6%) and 75(18.0%) of them had two and three drugs concomitant medications, respectively (table 3)

Table 3: Frequency of antihypertensive medicines prescribed for hypertensive patients at JUMC, 2018.

Classes	Specific drugs	Frequency(%), N=410
**ACEIs**	Enalapril	261 (63.7)
**ARBS**	Losartan	28 (6.8)
**Diuretics**		234 (57.1)
	Hydrochlorothiazide	203 (49.5)
	Furosemide	26 (6.4)
	Furosemide +spironolactone	5 (1.2)
**CCBs**		144 (35.1)
	Amlodipine	131 (31.9)
	Nifedipine	13 (3.2)
**BBs**		105 (25.6)
	Atenolol	45 (10.9)
	Metoprolol	47 (11.5)
	Propranolol	13 (3.2)
**Co-medications**	Total number of patients with co-medication	257 (61.7)
	One drug	61 (14.7)
	Two drugs	98 (23.6)
	Three drugs	75 (18.0)
	≥ Four drugs	23 (5.5)

ACEIs: Angiotensin-converting enzyme inhibitors; ARBs: angiotensin receptor blockers; BBs: βblockers; CCBs: calcium channel blockers

Of the total participants, 135 (32.5%) were on monotherapy and 275 (66.1%) were on combination therapy. Enalapril was the most frequently prescribed monotherapy 61(14.7%) followed by hydrochlorothiazide 40(9.6%). Among patients who were on combination therapy, two-drug regimens were prescribed in 46.4% of the hypertensive patients. ACEI +diuretics, 75

(18.0%) was a commonly used two-drug combination therapy. Three drugs combination accounted for 17(17.1%) of all prescriptions, of which 35 (8.4%) were on BB+ diuretics+ ACEI followed by CCB+ACEI +diuretic 22(5.3%). Four drugs regimen was prescribed in 11 hypertensive patients, ACEI +diuretics +CCB+BB 7(1.7%) was most frequent prescribed (Table 4).

Table 4: Regimens of antihypertensive therapy among hypertensive patients at JUMC, 2018

Regimens	Specific regimens	Frequency (%)
Non-pharmacologic		6 (1.4)
Monotherapy		135(32.5)
	Enalapril	61 (14.7)
	Amlodipine	26 (6.2)
	Nifedipine	4 (1.0)
	Hydrochlorothiazide	40 (9.6)
	Losartan	4 (1.0)
Dual therapy		193 (46.4)
	ACEI + Diuretic	75 (18.0)
	CCB + BB	6 (1.4)
	Diuretic + BB	16 (3.8)
	ACEI + CCB	30 (7.2)
	ACEI + BB	24 (5.8)
	Diuretic +ARB	4 (1.0)
	CCB + ARB	8 (1.9)
	CCB+ Diuretics	26 (6.3)
	ARB+BB	4 (1.0)
Triple therapy		71 (17.1)
	BB + Diuretic + ACEI	35 (8.4)
	CCB + BB + Diuretic	7 (1.7)
	CCB +ACEI + Diuretic	22 (5.3)
	CCB + BB + ACEI	1 (0.2)
	CCB + BB +ARB	3 (0.7)
	Others*	3 (0.7)
Quadruple		11 (2.6)
	ACEI + Diuretics + CCB + BB	7 (1.7)
	ARB + BB + CCB + diuretic	1 (0.2)
	Others**	3 (0.7)

others*: Enalapril +Furosemide +Spironolactone, Amlodipine +Furosemide + Hydrochlorothiazide; others**: Atenolol+ Enalapril+ Furosemide+ Spironolactone, Amlodipine+ Enalapril+ Furosemide+ Spironolactone

Clinicians’ adherence to hypertension treatment guidelines

Twenty-five full-time physicians treat hypertensive patients during the study period. There were more males (21) than females (4). The mean age of prescribers was 29.22 ± 4.51 with range 24-39 years and the majority (17) of them were below 30 years of age. Six of them were general practitioners (GPs), 4 residency year 2 (R2), 8 of them are residency year 3(R3) and 7 of them were internists. Nearly half of clinicians had experience 1-5 years and most of them treating more than 20 hypertensive patients per day. Most prescribers perceived that a JNC-8 hypertension treatment guideline was evidence-based and helpful in the management of patients. However, clinicians’ overall adherence to JNC-8 guidelines was low, with only 184 (44.2%) of prescribers following them. From a total of 232 patients not treated based on the JNC-8 guideline, 102(44.0%) patients were not received first-line drugs based on compelling indication and race. In 64(27.6%) patients, combination treatment was not adjusted based on their current BP level (Table 5).

Table 5: Common problems observed from prescribed antihypertensive medications to hypertensive patients at JUMC concerning the recommendation of JNC-8 guideline, N=232.

**Compliance issue**	**Frequency**	**Percent**
Patients did not receive first-line drugs based on compelling indication and race recommendation	102	44.0
Combination treatment was not adjusted based on their current blood pressure level	64	27.6
Patients received an inappropriate dose of medications	50	21.6
Patients were not on first-line drugs and the right dose of medication	16	6.9

Patients' Adherence to Hypertension Treatment

A total of 410 study participants received one or more antihypertensive medications. From patients' response to the eight-item Morisky medication adherence Scale, Overall, the prevalence of antihypertensive medication adherence 191 (46.6%)

Determinants of medication non-adherent among hypertensive patients at JUMC, 2018

In bivariate logistic regression analysis; age, sex, marital status, residency, occupation, monthly income, living status, salt use, khat chewing, physical activity status, concomitant medications, comorbid condition, and duration of treatment were variables with P-value less than 0.25 hence, included in the multivariate logistic regression. However, current occupation, physical activity status, and presence of DM comorbidity were significantly associated with medication non-adherence. Accordingly, merchants were 2.46 times (AOR=2.46, CI=1.16-5.23, P=0.020) more likely to be non-adherent than civil servants. Patients who had no regular physical activity were 1.63 times (AOR= 1.63, CI =1.04-2.55, P=0.033) more likely to be non-adherent compared to physically active patients. Hypertensive patients with DM comorbidity were 2.54 times (AOR= 2.54, CI=1.28-5.04, P=0.008) more likely to be non-adherent as compared to clients with no comorbidity (Table 6).

Table 6: Results of logistic regression analysis for factors associated with medication non-adherence among adult hypertensive patients at JUMC, 2018

**Variables**		**_Medication adherence**		**COR (95%CI) N=410**	**P-value**	**AOR (95%CI)**	**P-value**
		Adherent	Non Adherent				
**Occupation**	Civil servant	40	27	1.00	1.000	1.00	1.000
	Merchant	30	58	2.86(1.48-5.53)	0.002	2.46(1.16-5.23)	0.020
	Farmer	29	43	2.20(1.12-4.33)	0.023	1.41(0.52-3.78)	0.501
	House wife	24	22	1.36(0.64-2.89)	0.428	1.12(0.43-2.91)	0.812

	Retired	12	24	2.66(1.27-6.92)	0.012	2.04(0.66-6.26)	0.215
	Jobless	38	32	1.25(0.63-2.46)	0.522	0.71(0.26-1.91)	0.500
	Others	18	13	1.07(0.45-2.54)	0.878	0.73(0.26-2.08)	0.560
**Physical activity**	Inactive	89	130	1.67(1.13-2.48)	0.010	1.63(1.04-2.55)	0.033
	Active	102	89	1.00	1.000	1.00	1.000
**Comorbidities**	HTN alone	84	72	1.00	1.000	1.00	1.000
	DM	37	64	2.02(1.21-3.37)	0.007	2.54(1.28-5.04)	0.008
	CKD	11	5	0.53(0.18-1.59)	0.260	0.51(0.15-1.71)	0.272
	CHF	14	17	1.42(0.65-3.01)	0.378	1.14(0.48-2.71)	0.763
	CHD	13	25	2.24(1.10-4.70)	0.032	2.13(0.88-5.14)	0.093
	DM+CKD	11	5	0.53(0.18-1.60)	0.260	0.51(0.15-1.79)	0.294
	Dyslipidemia	17	21	1.44(0.71-2.94)	0.315	1.14(0.51-2.53)	0.751
	Others**	4	10	2.92(0.88-9.69)	0.081	2.81(0.77-10.22)	0.116

COR= crude odds ratio; AOR= Adjusted odds ratio: others**: Drivers, daily laborer, non-governmental organization and private work; others**: Anemia, peripheral neuropathy, lung disease, liver disease, thyroid disorder, and human immunodeficiency virus infection

Discussion

Medication adherence is the main predictor of treatment success and an important step in lowering BP. The present study showed that the overall rate of patients' medication adherence was 191 (46.6%). The finding was almost similar to the WHO report in 2011 in developing countries(50%) [38]. It was also comparable to studies done in Egypt (46.12%) and Saudi Arabia (46%) [39, 40]. It was, however, lower than studies reported from Taiwan (53%), Sweden (63.1%), and China (53.4%) [41, 42, 43]. The adherence discrepancy between the studies could be due to variations in the studied populations, better health care, and access to health in the developed countries. Moreover, differences in study results could be due to differences in measurement methodologies employed to assess drug adherence. For example, in Taiwan medication adherence was measured using the medication possession ratio (percentage of time that the patient had medication available to them during the follow-up period) whereas, in Sweden adherence was measured by using the Proportion of Days Covered (PDC) method.

This finding was also lower than the studies conducted in Gondar and Jimma university (64.6% and 61.8%, respectively) [44,45]. The discrepancy might be due to the varaition in the inclusion criteria and adherence measurement scale. Participants in the Jimma University study were

hypertension patients who had been followed for at least a year, and the sample size (280 patients) was lower than in this study. The Morisky 4-item Medication Adherence Scale was used to assess adherence at Gondar University. The medication adherence level found in this study, on the other hand, was higher than the finding from Ghana and Nigeria (30.3%) and Iran (24%) [46,47]. This disparity could be explained by the study done in Ghana and Nigeria included hypertensive patients who had depression which may have contributed to the low medication adherence. Similarly, in Iran, participants were selected from the rural area only which might have contributed to medication non-adherence.

In this study, a significant association was observed between patients' current occupation and medication nonadherence. Merchant hypertensive patients were 2.46 times more likely to be nonadherent than civil servants. This finding was in line with studies done in Hong Kong China and Black Loin Specialized Hospital, Ethiopia [48,49]. This might be because they forget to take drugs with themselves when they travel or leave home. Besides, they may be too busy to come to the health facility for their pills, and they may have difficulties in remembering to take all of their medicine on time. Physical inactivity was another important factor influencing patients' adherence in this study. Patients who had no regular physical activity were 1.63 times more likely to be non-adherent than physically active patients. This was consistent with a finding from Iran [47]. The exact mechanism was unclear, but it might be that physically inactive hypertensive patients will have uncontrolled BP (57.2 % in this study), prompting clinicians to prescribe a more complex treatment regimen. Furthermore, patients with uncontrolled BP might be hopeless to take their medications and adhere to their treatment plan.

In addition, the presence of DM as comorbidity was significantly associated with poor antihypertensive medication adherence. Hypertensive patients with DM comorbidity were 2.54 times more likely to be nonadherent than with no co-morbidities. This finding was similar to studies conducted in South Korea, Hong Kong China, Saudi Arabia, and Gondar, Ethiopia [10,48, 40, 44]. This could be explained by patients with co-morbidities who may have suffered from serious complications and had complex treatment regimens making it difficult for them to be adherent to their medications. Furthermore, 27.4% of participants in this study had no formal education, making it difficult to adhere to treatment plans and take complex treatment on time. This indicated that close supervision, short time appointments and fixed-dose combinations are important to ehance medication adherence for this group of patients.

This study found that 44.2% of clinicians' were adherent to hypertension treatment guidelines. The rate of adherence was slightly higher than the study conducted in Zewditu Memorial Hospital, Ethiopia (37.4%) [26]. This difference might be due to the small sample size, and most of the study participants (65.2%) were without co-morbid conditions in Zewditu Memorial Hospital. However, it was lower than studies conducted in South Indian (65%), Malaysia (85.30%), Island (70.4%), and South Africa (51.9%) [21, 23, 37, 29]. This disparity might be due to racial differences, as well as lack of laboratory and imaging facilities in our setting for further screening of target organ damage. In addition, it might be explained by the difference in prescribers profiles since the majority of doctors treating hypertensive patients in this study were general practitioners and residents. Six of them were general practitioners (GP), 4 residency year 2 (R2), 8 of them are residency year 3(R3) from the total of 25 doctors. The common challenges to adherence to JNC-8 hypertension treatment guidelines reported by prescribers were the cost of medication, lack of medication availability, and the presence of comorbidity. Ethiopias' Ministry of Health and other concerned bodies must continue to prioritize health care accessibility and affordability. The strong commitment of the Ethiopian ministry of health and the institution for health care improvement outcomes including fidelity, accesseblity of well-trained health care professionals, laboratory and imaging facilities for further screening for target organ damage, and program effectiveness are important to enhance clinicians guideline adherence in a health care setting.

According to JNC-8 guideline, CCBs and thiazide diuretics are the recommended first-line medicines for the management of hypertension for Africa origin. However, ACEIs were prescribed first, followed by diuretics and CCBs in this study (63.7%, 57.1%, 35.1% respectively). The most commonly prescribed class of drug among hypertensive patients with DM, CHF, or CHD was ACEI (66.3 percent, 71.0 percent, and 65.8 percent, respectively), whereas diuretics were the most commonly prescribed drug in CKD. This might be because prescribers (clinicians) were not adherent to standard treatment guidelines (JNC-8). ACEIs are preferable for CKD patients due to renal protective effects. The overall use of ACEIs was comparable to studies conducted in Serbia (60.57%) and Mexico City (63.8%) [30,31]. Thiazide diuretics were the most frequently prescribed medications in studies conducted in the Eastern Central Region of Portugal and rural tertiary hospitals in Nigeria showed that (67%, and 84.9%), respectively [29, 16]. This disparity could be attributed to studies in Portugal and Nigeria that used JNC-7 as a reference guideline, which is conservative in its use of thiazide-type diuretics as first-line therapy for most patients unless there is a compelling indication.

The result of this study showed that 135 (32.5%) patients were on mono-therapy regardless of the presence or absence of comorbidities, which was lower than the studies reported from Mexico (72.1%), Canada (56.3%), and Turkey (75.7%) [31-33]. The difference might be due to better health care, availability of medications, and close monitoring in these countries which help to achieve target BP with a single medication. Of the total of patients who were on monotherapy, ACEIs, 61 (14.7%) were the most prescribed drug classes. The result was closely similar to a study conducted in Kenya (20.2%) [34]. However, it was lower than the study conducted in Turkey (30.1%) [33]. The variation might be due to using different standard reference guidelines in which European guidelines recommend any class of drug as initial therapy. On contrary, the study conducted in Gondar hospital showed that thiazide diuretics were the most commonly prescribed mono-therapy (60.24%) [36]. this discrepancy might be because of the difference in level guideline adherence (66.8% prescription based on JNC-8 guidelines) that recommended the use of diuretics for both mono and combination therapy. In addition, variation may be different in the prevalence of CKD co-morbidity, as only 3.8% of participants had CKD comorbidity in this study.

On the other hand, the majority of the patients, 275(66.1%) were on combination therapy. The finding was consistent with the study conducted in Kenya (60%) [34]. It was also similar to a study conducted at Zewditu Memorial Hospital Ethiopia (70.8%) [26]. However, the finding of this study was higher than the study conducted in Malaysia (56.7%) [35]. The higher prescription rate of combination therapy in this study might be due to the low rate of BP control 42.8% in this study as compared to 84.6% in Malaysia.

Two drugs regimen accounted for 46.4 % of the combination therapy, of this 75(18%) were ACEIS + diuretics. The finding was in line with a study conducted in Kenya (14.5%) [34]. A study conducted in India using the JNC-8 guideline showed that the most frequently prescribed two-drug combination was ARB +diuretics [25]. This variation might be due to the cost and easy accessibility of ARB in India. On the other hand, in the study conducted in Nigeria, CCB + diuretic was the most frequently used two-drug combination (36.6%) [16]. This variation might be suggesting of doctor's preference to use the CCBs combination as initial therapy. Besides, in Nigeria majority of the patients were elderly (mean age was 61.5±15.1 years) hence, CCBs are preferable for older patients because isolated SBP is more prevalent due to vessel stiffness(50).

Limitations of the Study

This study has some limitations. The study was conducted in one facility, therefore; the findings may not be generalized to reflect all health care setting in Ethiopia. Only prescription and co-morbidity data were used to examine compliance to treatment guidelines, which may not always be reliable. Finally, this study was unable to identify factors affecting clinicians' adherence to standard treatment guidelines but will be an interesting area for future research.

Conclusion

The rate of medication adherence was poor among hypertensive patients. Being merchant, physical inactivity, and the presence of DM co-morbidity were factors associated with poor medication adherence. Prescribing patterns of antihypertensive drugs were inconsistent with JNC-8 guidelines. Angiotensin-converting enzyme inhibitors were the most frequently prescribed class of anti-hypertensive drugs. The majority of participants were on combination therapy. Effective education should be given to patients to improve medication adherence. Prescribers should be trained on treatment guidelines regularly to keep them up-to-date with current trends of hypertension treatment and for better treatment outcomes.

Abbreviation

BP: blood pressure, SBP: systolic blood pressure, DBP: Diastolic blood pressure, CHD: coronary heart disease, DM: diabetes mellitus, CKD: chronic kidney disease, CVD: cardiovascular diseases, IBM: international business machine, JNC-8: eight joint national committees, BB: beta-blocker, CCB: calcium channel blocker, ARB: angiotensin receptor blocker, ACEI: angiotensin-converting enzyme inhibitor, TD: thiazide diuretic, MMAS-8: Morisky medication adherence scale-8, AOR: adjusted odds ratio, COR: crude odds ratio.

Declarations

Conflict of interest: The authors declared that they have no conflict of interest.

Acknowledgments: We thank the participants of the study.

Availability of data and materials: **All relevant data are within the manuscript and its supporting information** files.

Authors' contributions: Bekalu Kebede: concept and design, data acquisition, interpretation of data, manuscript preparation; Legese Chelkeba: concept and design, interpretation of data, manuscript preparation, manuscript revision, and manuscript review. Bekalu Dessie: interpretation of data, manuscript preparation, manuscript revision, and manuscript review. All authors read and approved the final manuscript

References

1. Catherine P. Benziger GA. Roth AE. The Global Burden of Disease Study and the Preventable Burden of NCD. Journal of global heart.2016;11(4):393-397
2. Bromfield S, Muntner P. high blood pressure: the leading global burden of disease risk factor and the need for worldwide prevention programs. Curr Hypertens Rep. 2013;15(3):134-6.
3. Belue R, Okoror TA, Iwelunmor J, Taylor KD, Degboe AN, Agyemang C, et al. Globalization and Health An overview of cardiovascular risk factor burden in sub-Saharan African countries: a socio-cultural perspective. 2009; 12:1–12
4. Kibret KT, Mesfin YM. Prevalence of hypertension in Ethiopia: a systematic meta-analysis. Public Health Rev. 2015; 36:14.

5. Molla M. Systematic Reviews of Prevalence and Associated Factors of Hypertension in Ethiopia: Finding the Evidence. *Science Journal of Public Health*. 2015,3(4), 514-519.
6. Sarki AM, Nduka CU, Stranges S, Kandala NB, Uthman OA. Prevalence of Hypertension in Low- and Middle-Income Countries: A Systematic Review and Meta-Analysis. Medicine (Baltimore). 2015;94(50): e1959.
7. Ibrahim MM, Damasceno A. Hypertension in developing countries. Lancet. 2012;380(9841):611-9.
8. Grassi G, Cifkova R, Laurent S, Narkiewicz K, Redon J, Farsang C, et al. Blood pressure control and cardiovascular risk profile in hypertensive patients from central and eastern European countries. Eur Heart J. 2011; 32, 218–225
9. Schmieder RE, Mozaffarian D, Véronique L, Emelia J, Jarett D, Michael J et al. End Organ Damage in Hypertension. Circulation;2014; 129(3):1-19.
10. Shin S, Song H, Sang-Kwon Oh, Eob KC, Kim H, Jang S. Effect of antihypertensive medication adherence on hospitalization for cardiovascular disease and mortality in hypertensive patients. Hypertension Research.2013; 36(11):1000-1005.
11. Venkataraman R, Rashid M, Shashikantha B, Soumya A, Vijayan G, Manuel GG, Islam S. Prescribing pattern of antihypertensive medication and adherence to Joint National Commission-8 guidelines in a rural tertiary care Indian teaching hospital. Journal of basic and clinical physiology and pharmacology. 2020 Jan 1;31(1).
12. Ettehad D, Emdin CA, Kiran A, Anderson SG, Callender T, Emberson J, et al. Blood pressure lowering for prevention of cardiovascular disease and death: a systematic review and meta-analysis. Lancet, 2016; 387(10022), 957–967.
13. Jellinger PS, Handelsman Y, Rosenblit PD, Bloomgarden ZT, Fonseca VA, Garber AJ, Grunberger G, etal. American association of clinical endocrinologists and American college of endocrinology guidelines for management of dyslipidemia and prevention of cardiovascular disease. Endocr pract. 2017 apr;23(suppl 2):1-87
14. James PA, Oparil S, Carter BL, Cushman WC, Dennison-Himmelfarb C, Handler J etal. 2014 Evidence-Based Guideline for the Management of high blood pressure in adults reports from the panel members appointed to the eighth Joint National Committee (JNC 8). JAMA. 2014;311(5):507-20
15. Gebrihet TA, Mesgna KH, Gebregiorgis YS, Kahsay AB, Weldehaweria NB, Weldu G. Awareness, treatment, and control of hypertension is low among adults in Aksum town, northern Ethiopia: A sequential quantitative-qualitative study. PLoS One. 2017;12(5):e0176904.
16. Busari OA, Oluyombo R, Fasae AJ, Gabriel EO, Ayodele LM, Agboola SM. Prescribing pattern and utilization of antihypertensive drugs and blood pressure control in adult patients with systemic hypertension in a rural tertiary hospital in Nigeria. Am J Inter Med. 2014;2(6):144-9

17. Parati G, Omboni S, Compare A, Grossi E, Callus E, Venco A, et al. Blood pressure control and treatment adherence in hypertensive patients with metabolic syndrome: protocol of a randomized controlled study based on home blood pressure telemonitoring vs. conventional management and assessment of psychological determinants of adherence(TELEBPMET Study). trials; 2013;1–11.
18. Asgedom SW, Gudina EK, Desse TA. Assessment of Blood Pressure Control among Hypertensive Patients in Southwest Ethiopia. PLoS One. 2016;11(11):e0166432.
19. Papatya Karakurt, Mağfiret Kaşikçi, Factors affecting medication adherence in patients with hypertension, Journal of Vascular Nursing. 201230(4), 118-126,
20. Ahmed A, Saqlain M, Tanveer M, Nawaz MS, Rehman K, Safdar A, Tayyeb M, Ali Z, Shinwari M, Baig RU. Prescribing Patterns of Antihypertensive Drugs in Patients Attending Tertiary Care Hospitals in Pakistan. SN Comprehensive Clinical Medicine. 2021 Jan;3(1):176-82.
21. Datta S. Utilization Study of Antihypertensives in a South Indian Tertiary Care Teaching Hospital and Adherence to Standard Treatment Guidelines. J Basic Clin Pharm. 2016;8(1):33-37.
22. Mutua EM, Gitonga MM, Mbuthia B, Muiruri N, Cheptum JJ, Maingi T. Level of blood pressure control among hypertensive patients on follow-up in a regional referral hospital in Central Kenya. Pan Afr Med J. 2014;18:278.
23. Abdulameer SA, Sahib MN, Aziz NA, Hassan Y, Alrazzaq HA, Ismail O. Physician adherence to hypertension treatment guidelines and drug acquisition costs of antihypertensive drugs at the cardiac clinic: a pilot study. Patient Prefer Adherence. 2012;6:101-8.
24. An J, Luong T, Qian L, Wei R, Liu R, Muntner P, Brettler J, Jaffe MG, Moran AE, Reynolds K. Treatment Patterns and Blood Pressure Control With Initiation of Combination Versus Monotherapy Antihypertensive Regimens. Hypertension. 2021 Jan;77(1):103-13..
25. Romday R, Gupta A, Bhambani P. An assessment of antihypertensive drug prescription patterns and adherence to joint national committee-8 hypertension treatment guidelines among hypertensive patients attending a tertiary care teaching hospital. International Journal of Research in Medical Sciences. 2016;4(12).5125-33.
26. Yazie D, Shibeshi W, Woldu M, Berha A. Assessment of Blood Pressure Control among Hypertensive Patients in Zewditu Memorial Hospital, Addis Ababa, Ethiopia: A Cross-Sectional Study. Journal of Bioanalysis & Biomedicine. 2018;10.1-53
27. Beger C, Unger T, Haller H, Limbourg FP. Antihypertensive prescription patterns and cardiovascular risk in patients with newly diagnosed hypertension-an analysis of statutory health insurance data in Germany. Blood Pressure. 2020 Nov 1;29(6):357-61.
28. Morgado MP, Rolo SA, Pereira L, Castelo-Branco M. Blood pressure control and antihypertensive pharmacotherapy patterns in a hypertensive population of Eastern Central Region of Portugal. BMC Health Serv Res. 2010;10:349.

29. Adedeji AR, Tumbo J, Govender I. Adherence of doctors to a clinical guideline for hypertension in Bojanala district, North-West Province, South Africa. Afr J Prim Health Care Fam Med. 2015;7(1):776.
30. Tomas A, Tomić Z, Milijasević B, Ban M, Horvat O, Vukmirović S et al. Patterns of prescription antihypertensive drug utilization and adherence to treatment guidelines in the city of Novi Sad. Vojnosanit Pregl. 2016;73(6):531-7.
31. Alba-Leonel A, Carvajal A, Fierro I, Castillo-Nájera F, Campos-Ramos O, Villa-Romero A et al. Prescription patterns of antihypertensives in a community health center in Mexico City: a drug utilization study. Fundam Clin Pharmacol. 2016; 30(3):276-81.
32. Beaulieu MD, Dufresne L, LeBlanc D. Treating hypertension. Are the right drugs given to the right patients? Can Fam Physician. 1998; 44:294-8, 301-2.
33. Abaci A, Kozan O, Oguz A, Sahin M, Deger N, Senocak H et al. Prescribing pattern of antihypertensive drugs in primary care units in Turkey: results from the TURKSAHA study. Eur J Clin Pharmacol. 2007 Apr;63(4):397-402.
34. Mbui JM, Oluka MN, Guantai EM, Sinei KA, Achieng L, Baker A et al. Prescription patterns and adequacy of blood pressure control among adult hypertensive patients in Kenya; findings and implications. Expert Rev Clin Pharmacol. 2017; 10(11):1263-1271.
35. Ramli A, Miskan M, Ng K, Ambigga D, Nafiza M, Mazapuspavina M, Sajari J, Ishak R. Prescribing of Antihypertensive Agents in Public Primary Care Clinics - is it per Current Evidence? Malays Fam Physician. 2010;5(1):36-40.
36. Abegaz TM, Tefera YG, Abebe TB. Antihypertensive drug prescription patterns and their impact on the outcome of blood pressure in Ethiopia: a hospital-based cross-sectional study. Integr Pharm Res Pract. 2017; 6:29-35.
37. Theodorou M, Stafylas P, Kourlaba G, Kaitelidou D, Maniadakis N, Papademetriou V. Physicians' perceptions and adherence to guidelines for the management of hypertension: a national, multicentre, prospective study. Int J Hypertens. 2012;2012:503821.
38. Brown MT, Bussell JK. Medication adherence: WHO cares? Mayo Clin Proc. 2011; 86(4):304-14.
39. Hussein A, Awad MS, Mahmoud HE. Patient adherence to antihypertensive medications in upper Egypt: a cross-sectional study. The Egyptian Heart Journal. 2020 Dec;72:1-8.
40. Khayyat SM, Khayyat SM, Hyat Alhazmi RS, Mohamed MM, Abdul Hadi M. Predictors of Medication Adherence and Blood Pressure Control among Saudi Hypertensive Patients Attending Primary Care Clinics: A Cross-Sectional Study. PLoS One. 2017;12(1):e0171255.
41. Lee CY, Huang CC, Shih HC, Huang KH. Factors influencing antihypertensive medication compliance in Taiwan: a nationwide population-based study. Eur J Prev Cardiol. 2013; 20(6):930-7.

42. Hedna K, Hakkarainen KM, Gyllensten H, Jönsson AK, Andersson Sundell K, Petzold M, Hägg S. Adherence to Antihypertensive Therapy and Elevated Blood Pressure: Should We Consider the Use of Multiple Medications? PLoS One. 2015;10(9):e0137451.
43. Li YT, Wang HHX, Liu KQL, Lee GKY, Chan WM, Griffiths SM et al. Medication Adherence, and Blood Pressure Control Among Hypertensive Patients With Coexisting Long-Term Conditions in Primary Care Settings: A Cross-Sectional Analysis. Medicine (Baltimore). 2016; 95(20):e3572.
44. Ambaw AD, Alemie GA, W/Yohannes SM, Mengesha ZB. Adherence to antihypertensive treatment and associated factors among patients on follow-up at University of Gondar Hospital, Northwest Ethiopia. BMC Public Health. 2012;12:282.
45. Asgedom SW, Atey TM, Desse TA. Antihypertensive medication adherence and associated factors among adult hypertensive patients at Jimma University Specialized Hospital, southwest Ethiopia. BMC Research Notes. 2018;11(1):27.
46. Boima V, Ademola AD, Odusola AO, Agyekum F, Nwafor CE, Cole H, et al. Factors Associated with Medication Nonadherence among Hypertensives in Ghana and Nigeria. Int J Hypertens. 2015;2015:205716.
47. Kamran A, Sadeghieh Ahari S, Biria M, Malepour A, Heydari H. Determinants of Patient's Adherence to Hypertension Medications: Application of Health Belief Model Among Rural Patients. Ann Med Health Sci Res. 2014; 4(6):922-7.
48. Kang CD, Tsang PP, Li WT, Wang HH, Liu KQ, Griffiths SM et al. Determinants of medication adherence and blood pressure control among hypertensive patients in Hong Kong: a cross-sectional study. Int J Cardiol. 2015 Mar 1;182:250-7.
49. Hareri HA, Abebe M and Asefaw T: Assessments of adherence to Hypertension managements and its influencing factors among Hypertensive patients attending Black Lion Hospital chronic follow up a unit, Addis Ababa, Ethiopia-a cross-sectional study. *Int J Pharm Sci Res* 2013; 4(3); 1086-1095.
50. Ogundele SO, Ajibare AO, Amisu MA, Dada AO. Prescription pattern and blood pressure control among patients on antihypertensive medications attending a tertiary hospital in Lagos: A cross-sectional study. Nigerian Journal of Basic and Clinical Sciences. 2021 Jan 1;18(1):9.

First, we would like to appreciate the editor and reviewers for giving us another round of invaluable comments so as to revise our manuscript accordingly. We have gone through all the comments given by the reviewers and revised the manuscript point by point.

Response to editors /editorial office

S. No	Comments/ Journal Requirements:	Response by authors
1	Please review your reference list to ensure that it is complete and correct	The authors attempted to incorporate some updated references and were highlighted in manuscript with track change. Also we checked its appropriateness
2	Is the manuscript technically sound, and do the data support the conclusions?	Authors tried to revised the manuscript, rearrange and correct some technical issue such as editorial problem in extensive manner. In addition, we attempted to present key finding with available evidence from a given data and come up with conclusion accordingly.
3	Is the manuscript presented in an intelligible fashion and written in standard English?	As we mentioned above the manuscript was revised extensively with two different persons who are expert in English language

Response to Reviewer

**Response to Reviewer 2**		
1	I can still see the statements that do not sound so interesting. For example on page 2 the writers said, “Besides, Ethiopia has not its hypertension treatmentguideline”. This can be captured as “Besides, Ethiopia does not have a treatment guideline for hypertension**”.**	It was corrected as per comment given by reviewer and a whole manuscript was revised.<br>Besides, Ethiopia does not have a treatment guideline for hypertension
2	Page 18, there is nothing under Declaration. This should have a statement on conflict of interest. I strongly feel you need someone to read and correct some of the phrases in your manuscript	**Declarations**<br>**Conflict interests: The authors declared that they have no conflict of interest.**
**Response for Reviewer 3**		

1	There are still a few typographical and grammatical errors that the authors need to address. These have been highlighted in the comment section of the manuscript.	Authors accept all comments given by reviewer ≠3 including mentioned at attached file and attempted to correct accordingly.
B	The discussion section needs to be beefed up- it should include a discussion of the implications of the findings on policy and practice, and not a mere comparison of findings with other studies in literature. Suggestions have been made in the comments section of the manuscript. Only the key findings should be discussed; as opposed to what the authors have done, discussing every finding.	We tried to discuss only key findings with its implication and highlighted in main manuscript.
3	Tables and figures should be reduced- authors should see which ones to remove (reviewer has suggested one) so that only the prescribed number of tables is included , with the rest provided in the supplementary material section	Table 5 is main part of manuscript. As per this journal requirements files to be a supplementary materials if it is not main part of manuscript.

Thank you
