# Supplementary material for: Clinicians’ prescribing pattern, rate of patients’ medication adherence and its determinants among adult hypertensive patients at Jimma University Medical Center: Prospective cohort study
Source: PLoS One. 2021 Nov 15;16(11):e0259421. doi: 10.1371/journal.pone.0259421 (PMC8592482; doi:10.1371/journal.pone.0259421)
Supplement: Attachment — Submitted filename: Response to Reviewers.docx [file pone.0259421.s005.docx]

First, we would like to appreciate the editor and reviewers for giving us another round of invaluable comments so as to revise our manuscript accordingly. We have gone through all the comments given by the reviewers and revised the manuscript point by point.

**Response to editors /editorial office**

| S. no | Comments/concerns | Response to comments |
| --- | --- | --- |
| 1 | Please ensure that your manuscript meets PLOS ONE's style requirements, including those for file naming. | We tried to prepare and re-write our manuscript based on PLOSE ONE manuscript body formatting guidelines. |
| 2 | Please include additional information regarding the survey or questionnaire used in the study and ensure that you have provided sufficient details that others could replicate the analyses. For instance, if you developed a questionnaire as part of this study and it is not under a copyright more restrictive than CC-BY, please include a copy, in both the original language and English, as Supporting Information. Moreover, please include more details on how the questionnaire was pre-tested, and whether it was validated. | Questionnaire used in the study was developed by reviewing different literatures from similar studies. Pre-test of data collection format was performed on 5% (21) of the sample before conducting the study in one of local hospital found in Ethiopia which is specialized teaching hospital like that of study area. Then, the final tool was developed with some modifications after reviewing the results of the pre-test. The questionnaire was validated by an expert in the field of pharmacy practice for length, readability, and relevance. |
| 3 | Please provide additional details regarding participant consent. In the ethics statement in the Methods and online submission information, please ensure that you have specified how verbal consent was documented and witnessed. | This study was approved by the Ethical Review Board of School of Pharmacy, Institute of Health Science Jimma university (Ref.No IHRPGD/203/18). Prior to data collection, individual informed verbal consent was obtained from the study participants. Written informed consent was waived since the study did not involve any procedure and present no damage to patients as approved by the ethical review board committee of Board of School of Pharmacy, Institute of Health Science Jimma university. Patients were informed about the objective of the study; procedures of selection and assurance of confidentiality and their names was not registered to minimize social desirability bias and enhance anonymity. They were not forced to participate or receive any monetary incentive and it was solely voluntary based. |
| 4 | We suggest you thoroughly copyedit your manuscript for language usage, spelling, and grammar. If you do not know anyone who can help you do this, you may wish to consider employing a professional scientific editing service. | The manuscript was extensively revised by a person who is expert in the field of English language |
| 5 | Please remove any funding-related text from the manuscript | Done |
| 6 | Your ethics statement should only appear in the Methods section of your manuscript. If your ethics statement is written in any section besides the Methods, please move it to the Methods section and delete it from any other section. Please ensure that your ethics statement is included in your manuscript, as the ethics statement entered into the online submission form will not be published alongside your manuscript. | ???? |

**Response to additional comments given**

| S. No | Comments | Response by authors |
| --- | --- | --- |
| 1 | Is the manuscript technically sound, and do the data support the conclusions? | Authors tried to revised the manuscript, rearrange and correct some technical issue such as editorial problem in extensive manner. In addition we attempted to present key finding with available evidence from a given data and come up with conclusion accordingly. |
| 2 | Has the statistical analysis been performed appropriately and rigorously? | Authors strongly believed that this study carried out by using appropriate statically analysis methods and the finding interpreted in scientific way. |
| 3 | Have the authors made all data underlying the findings in their manuscript fully available? | Authors attached additional figures and tables as supporting information |
| 3 | Is the manuscript presented in an intelligible fashion and written in standard English? | As we mentioned above the manuscript was revised extensively with a person who is expert in English language |
| 4 | Age classification 60 and >60 seems to be very abstract and does not talk much about the study population  It would be advisable to classify patients into age groups so that the reader can easily understand the patient demographic | This classification system is based on JNC-8 hypertension treatment guideline for goal of blood pressure management |

**Response to Reviewer 1**

| S. No | Comments | Response |
| --- | --- | --- |
| 1 | Authors need to rework on the manuscript for better clarity. The language is not clear. The methodology is also not robust. The number of tables are also more withlengthy descriptions. Discussion section also requires rewriting and conclusion has to be based upon the findings and the possible recommendations | As per comment given, a whole manuscript was revised including language, methodology, tables and figures. Discussion and  recommendation also rewrite based upon the key finding |
| **Response to Reviewer 2** | | |
| 1 | **Introduction**  There are a lot of issues here. For example, you mentioned on page 3 that "Ethiopia has not its own hypertension treatment guideline and on the time being this guideline widel yusing by both clinicians and clinical pharmacist [16]". What exactly do you mean? The sentence is confusing | That means, Ethiopia has not its own hypertension treatment guideline. Therefore, JNC-8 guideline is widely used by both clinicians and clinical pharmacist in Ethiopia [16]. |
| 2 | **Method** Please what do you mean by patients were consequently followed for the next three months? Was done done daily, weekly, monthly, etc for all participants (page 5)? Your sample size was calculated based on patients' adherence to treatment; do you think that this will adequately cater for physicians adherence to guidelines? Page 5, please what do you mean by "questioner"? Please check your definition of Guideline adherence. I think the denominator "total number of hypertensive patients treated". I also think that the Operational Definition should beat the Appendix and referred to periodically. Page 5, please how were your BP measurements taken? | Only those patients selected at first month were consequently followed every month for the next three regarding their life stay modification medication adherence status and what medication prescribed for them. In this study all clinicians who treated hypertensive patients during study period were included. Questioner is to mean questionnaire it is spelling error  At Jimma University ambulatory cardiac center, initial and subsequent BP typically measured using the cuff sphygmomanometer and digital sphygmomanometer on the left arm, at the level of the heart of the patient, who was in a sitting position and at rest for at least 5 minute. This  information typically recorded in the patients’ notes |
| 3 | **Results**  Your tables, in particular table 6 should be reformatted. It is very difficult to following the distributions.  Table 3, please why do you put those with one medication under co-medication? Enalapril constitutes 63.7% of ACEIs, where are the rest? Same with ARBs. Why have you presented ACEIs and ARBs different from Diuretics, CCBs and BBs?  Table 6 is not clear at all. I could not read this well, but there are issues with it. | Table 6 re- arranged by presented only variables that were statically significant for poor medication adherence. In table 3 co-medication means adjuvant or other medication other than anti-hypertensive such as statins, anti-platelet, anti-diabetes etc.  Table 3 showed Frequency of all antihypertensive medicines prescribed for hypertensive patients. Medications/class of medication presented separately because of JNC-8 guideline recommendation for white and black race (Africa origin) population. Initial treatment for nonblack population thiazide type diuretics (TDs) or angiotensin converting enzyme inhibitors (ACEIs ) or angiotensin receptor blockers (ARBs) or calcium channels blockers (CCBs), alone or in combination and for black population, TDs or CCBs, alone or in combination |
| 4 | **Conclusion** Because most of your information are on Table 6 and I could not read this well, I did not review your discussion.. | Authors accept a given comment and correct acordingly |
| **Response for Reviewer 3** | | |
| 1 | **Reviewer #3**: There are many grammatical errors in the manuscript that tend to lose the reader. They have been included in the reviewed manuscript uploaded. The manuscriptlooks like it has been copy pasted from a dissertation/thesis; only key information should be included and the rest uploaded. These sections have been highlighted in theattached manuscript. There are very minimal results on the clinicians' adherence to treatment guidelines and these results are not discussed, yet they seem to form a huge partof this manuscript. In the discussion section, the authors should discuss only the key findings and not every finding and include the implications of such findings. This has been scantily done. There are too many tables and figures in the manuscript- the authors should include only key findings and present the rest as attachments. The authors should review author guidelines and revise the manuscript accordingly**.** | Authors accept all comments given by reviewer ≠3 and attempted to correct accordingly. |

Thank you
