# Supplementary material for: Clinicians’ prescribing pattern, rate of patients’ medication adherence and its determinants among adult hypertensive patients at Jimma University Medical Center: Prospective cohort study
Source: PLoS One. 2021 Nov 15;16(11):e0259421. doi: 10.1371/journal.pone.0259421 (PMC8592482; doi:10.1371/journal.pone.0259421)
Supplement: Attachment — Submitted filename: Response to reviewers.docx [file pone.0259421.s007.docx]

First, we would like to appreciate the editor and reviewers for giving us another round of invaluable comments so as to revise our manuscript accordingly. We have gone through all the comments given by the reviewers and revised the manuscript point by point.

**Response to editors /editorial office**

| S. No | Comments/ Journal Requirements: | Response by authors |
| --- | --- | --- |
| 1 | Please review your reference list to ensure that it is complete and correct | The authors attempted to incorporate some updated references and were highlighted in manuscript with track change. Also we checked its appropriateness |
| 2 | Is the manuscript technically sound, and do the data support the conclusions? | Authors tried to revised the manuscript, rearrange and correct some technical issue such as editorial problem in extensive manner. In addition, we attempted to present key finding with available evidence from a given data and come up with conclusion accordingly. |
| 3 | Is the manuscript presented in an intelligible fashion and written in standard English? | As we mentioned above the manuscript was revised extensively with two different persons who are expert in English language |

**Response to Reviewer**

| **Response to Reviewer 2** | | |
| --- | --- | --- |
| 1 | I can still see the statements that do not sound so interesting. For example on page 2 the writers said, “Besides, Ethiopia has not its hypertension treatmentguideline”. This can be captured as “Besides, Ethiopia does not have a treatment guideline for hypertension**”.** | It was corrected as per comment given by reviewer and a whole manuscript was revised.  Besides, Ethiopia does not have a treatment guideline for hypertension |
| 2 | Page 18, there is nothing under Declaration. This should have a statement on conflict of interest. I strongly feel you need someone to read and correct some of the phrases in your manuscript | **Declarations**  **Conflict interests: The authors declared that they have no conflict of interest.** |
| **Response for Reviewer 3** | | |
| 1 | There are still a few typographical and grammatical errors that the authors need to address. These have been highlighted in the comment section of the manuscript. | Authors accept all comments given by reviewer ≠3 including mentioned at attached file and attempted to correct accordingly. |
| B | The discussion section needs to be beefed up- it should include a discussion of the implications of the findings on policy and practice, and not a mere comparison of findings with other studies in literature. Suggestions have been made in the comments section of the manuscript. Only the key findings should be discussed; as opposed to what the authors have done, discussing every finding. | We tried to discuss only key findings with its implication and highlighted in main manuscript. |
| 3 | Tables and figures should be reduced- authors should see which ones to remove (reviewer has suggested one) so that only the prescribed number of tables is included , with the rest provided in the supplementary material section | Table 5 is main part of manuscript. As per this journal requirements files to be a supplementary materials if it is not main part of manuscript. |

Thank you
